# Clinical and economic outcomes associated with fidaxomicin in comparison to vancomycin, metronidazole, and FMT: A systematic literature review

**DOI:** 10.1097/MD.0000000000039219

**Published:** 2024-12-27

**Authors:** Qinghua Li, Engels Obi, Anne Marciniak, Rebecca Newman, Isabelle Whittle, Jason Kufakwaro

**Affiliations:** a Merck & Co., Inc., Rahway, NJ; b Adelphi Values PROVE, Bollington, UK.

**Keywords:** burden, clinical, *Clostridium difficile*, economic, effectiveness, fecal microbiota transplantation, fidaxomicin, infectious disease, metronidazole, systematic review, vancomycin

## Abstract

**Background:**

There are an estimated half a million cases of *Clostridioides difficile* infection (CDI), in the United States annually. Fidaxomicin, vancomycin, and metronidazole are commonly used for CDI treatment, with fidaxomicin recommended by clinical guidelines as the preferred treatment for initial and recurrent CDI. This systematic literature review aimed to explore clinical and economic outcomes associated with fidaxomicin use with or without comparison to vancomycin, metronidazole, or fecal microbiota transplantation (FMT).

**Methods:**

The EMBASE, Medline, EconLit, and Evidence Based Medicine Reviews databases were searched from January 1st, 2012 to December 6th, 2022, as fidaxomicin was first approved for adult use in 2011. Identified publications were assessed and extracted by 2 independent reviewers.

**Results:**

Seventy-nine publications were included. Articles reporting at least 50 patients with follow-up ≤90 days were selected to obtain comparable outcome definitions (N = 14). Sustained clinical cure rate at 30- and 60-days follow-up was higher among fidaxomicin-treated patients (70.0–75.1% and 63.2–78.9%; N = 3) than vancomycin (45.1–58.2% and 38.9–50.0%; N = 3). Lower recurrence rates were reported post-fidaxomicin treatment compared to vancomycin, however the ranges overlapped at 30-, 60-, and 90-days follow-up. Limited outcomes for comparators metronidazole and FMT were identified. Healthcare resource use data were limited, with 2 studies reporting direct costs finding that fidaxomicin use-associated savings were driven by reduced hospital admission-related costs. Fidaxomicin was cost-effective in 14 of 21 economic analyses (11 vs vancomycin). Three studies reported vancomycin or FMT as more cost-effective than fidaxomicin. Fidaxomicin was consistently cost-effective or cost-saving among patients receiving concomitant antibiotics, and patients with cancer or renal impairment. Ten publications reported that the higher acquisition cost of fidaxomicin was offset by reduced recurrence and hospital readmission costs.

**Conclusions:**

Fidaxomicin was clinically effective compared to vancomycin. Fidaxomicin is often reported as cost-effective, consistently within high-risk subpopulations.

## 1. Introduction

*Clostridioides difficile* (*C. difficile*) is the bacterium responsible for *C. difficile* infection (CDI), a gastrointestinal infection. Diarrhea is the most common symptom of CDI, often referred to as *C. difficile*-associated diarrhea (CDAD), although abdominal cramps, fever, dehydration, leukocytosis, and nausea are also common symptoms.^[1,2]^ CDI frequently requires healthcare intervention, particularly for severe complications including electrolyte imbalance, systemic inflammatory response syndrome, renal failure, hypotension, toxic megacolon, colonic perforation, and death.^[2,3]^

CDI cases are typically classified by the US Centers for Disease Control and Prevention (CDC) based on where the infection was acquired, either as healthcare-acquired (HA-CDI) or community-acquired cases (CA-CDI).^[4,5]^ Both HA-CDI and CA-CDI share common risk factors in antibiotic treatment and increased age.^[4,5]^ The most recent 2020 surveillance data reported that CA-CDI accounts for 51.2% of cases in North America, whereas HA-CDI accounted for 50.1%.^[6]^ In 2017, the total number of CA-CDI cases in the United States (US) was 226,400.^[7]^ A retrospective cohort study conducted across 43 hospitals in the US reported that CA-CDI led to an increase in hospitalizations from 17.1 per 100,000 general population in 2011 to 21.7 per 100,000 general population in 2017 (among the general population across 10 US states).^[8]^ A retrospective cohort study conducted in 2022 among Medicare beneficiaries aged ≥65 years reported an all-cause mortality rate of 31.9% among patients diagnosed with primary CDI, with CDI-associated mortality responsible for 2.7% of deaths in this cohort.^[9]^ All-cause mortality rate among this cohort of adults aged ≥65 years with recurrent *Clostridioides difficile* infection (rCDI) was 10.8%, of which CDI was responsible for 25.4%.^[9]^

Key risk factors for CDI include antibiotic use and other healthcare exposures like hospital admission, surgery, an emergency room visit, outpatient encounters and long-term healthcare facility residence.^[4,10,11]^ Populations at increased risk of CDI and more severe outcomes include older adults aged ≥65 years, and people who are immunocompromised (e.g., due to human immunodeficiency virus/acquired immunodeficiency syndrome, taking immunosuppressive drugs).^[5,12]^

Recurrent CDI is the occurrence of additional episodes of CDI posttreatment for the initial *C. difficile* infection. Following an initial episode of CDI up to 35% of patients will experience CDI recurrence, and up to 60% of patients with previous CDI recurrence will experience further recurrences.^[13]^ Recurrent CDI is the main driver of clinical and economic burden as it increases rates of healthcare resource utilization (HCRU). For example, in the US, recent estimates (2020) of both emergency department (ED) and outpatient visits increased with recurrence (mean ED visits: 1.4 for first recurrences to 4.6 visits for ≥ 3 recurrences per year; outpatient: 15.4 visits for ≤2 episodes of rCDI vs 26.3 visits for ≥3 recurrences per patient year).^[14]^ Further, patients with rCDI experience longer mean hospital stays than patients with primary CDI (9.3 vs 7.3 cumulative hospitalized days, respectively).^[14]^

Current treatment guidelines from the Infectious Diseases Society of America (IDSA) and the Society for Healthcare Epidemiology of America (SHEA) state that fidaxomicin (standard or extended-pulse) use is preferred over vancomycin use in the treatment of patients with an initial CDI or recurrent episode.^[1,2]^ Fidaxomicin use is permitted for first recurrence only if fidaxomicin was not used for the treatment of initial infection. Additionally, treatment with fecal microbiota transplantation (FMT) is preferred for subsequent recurrences after first recurrence.^[15]^

The burden of initial and rCDI persists despite the availability of therapeutic options and preventive measures such as isolation and facility antibiotic stewardship programs.^[16]^ Further, even with the latest clinical recommendation of fidaxomicin as first-line treatment for initial and recurrent CDI, uptake rate remains relatively low with higher rates of vancomycin uptake.^[1,17]^ Therefore, it is important to collectively review clinical and economic outcomes associated with fidaxomicin use to optimize medical decision-making for CDI.

The aim of this systematic literature review (SLR) was to summarize evidence on the clinical and economic outcomes associated with the use of fidaxomicin with or without comparison to vancomycin, metronidazole, or FMT.

## 2. Methods

### 2.1. Search strategy

A comprehensive search was developed within the OVID^®^ platform, and conducted across 4 electronic databases (EMBASE, Medline, EconLit, and Evidence Based Medicine Reviews). The search strategy was developed in alignment with the Cochrane Handbook for Systematic Reviews of Interventions, and used key terms specifying clinical and economic outcomes in patients with initial or recurrent CDI or CDAD who were treated with fidaxomicin, and that were published in English between January 1, 2012 and December 6, 2022.^[18]^ As fidaxomicin (DIFICID™) was first approved for adult use by the US Food and Drug Administration in 2011, this time period was appropriate to capture all relevant data generated after the initial pivotal clinical trials.^[19]^ Of note, the indication for fidaxomicin was extended to children aged 6 months and over in 2020, therefore available pediatric data would also be captured.^[19]^ The OVID^®^ database search strategy is included in Table S1, Supplemental Digital Content, http://links.lww.com/MD/N353.

To ensure a comprehensive evidence base, the database searches were supplemented by gray literature searches for literature published from January 1, 2021 to December 6, 2022. Conference proceedings from IDWeek, International Society for Pharmacoeconomics and Outcomes Research, Digestive Disease Week, European Society of Clinical Microbiology and Infectious Disease, and American College of Gastroenterology were identified. IDWeek is the joint annual meeting of the IDSA, SHEA, the Human Immunodeficiency Virus Medicine Association, the Pediatric Infectious Diseases Society, and the Society of Infectious Diseases Pharmacists.

Ethical approval was not required as this research was based on previously conducted studies and did not involve any new studies with human participants or animals.

### 2.2. Study selection and data extraction

Abstracts and full texts of identified studies were dual screened by 2 independent researchers against prespecified eligibility criteria based on population, intervention, comparator, outcomes, time, study design criteria (Table 1). Any discrepancies were resolved by involvement of a third senior reviewer.

**Table 1:** PICOTS criteria for study inclusion.

	Inclusion criteria	Exclusion criteria
Population(s)	Patients with initial or recurrent CDI or CDAD	Patients without CDI or CDAD
Interventions	– Fidaxomicin	Any intervention not mentioned in inclusion criteria
Comparators	– Vancomycin – Metronidazole (oral or intravenous administration) – FMT – Placebo – No comparator	Any intervention not mentioned in the inclusion criteria
Outcomes	Clinical: – Clinical cure – CDI-related hospitalizations – CDI-related mortality (30-day, 90-day) – CDI recurrence rates – Sustained response rates – CDI-related readmissions (30-day, 90-day) – All-cause readmissions (30-day, 90-day) – Acute care days post-discharge (30-day, 90-day) – Cases of antibiotic resistance – Antibiotic overuse Economic: – Economic analyses (e.g., cost-effectiveness, budget impact, cost minimization) – Relevant economic model inputs (e.g., disease prevalence/incidence, HCRU unit costs) and outputs (e.g., direct/indirect costs) – Direct costs – HCRU – Indirect costs – Caregiver costs	Any outcome not included in the inclusion criteria
Time	Studies published from January 1st, 2012 to December 6th, 2022	Studies published prior to 2012
Study design	RCT (randomized controlled trial), single arm, comparative, descriptive, economic modeling, systematic review, observational studies, real-world evidence	Nonsystematic reviews, commentaries, editorials, case studies, case series, letters to the editor.
Other	English language publications, human studies	Studies not published in English, nonhuman studies

CDAD = *Clostridioides difficile*-associated diarrhea, CDI = *Clostridioides difficile* infection, FMT = fecal microbiota transplantation, HCRU = healthcare resource use, PICOTS = population, intervention, comparator, outcomes, time, study design, RCT = randomized controlled trial.

Data extraction was conducted in Microsoft Excel^®^ using a customized data extraction form. Data were extracted by 1 reviewer and checked by a second reviewer, with discrepancies resolved by discussion or involvement of a third senior reviewer. From each included study, publication information, study characteristics, population characteristics, and outcomes of interest were extracted.

### 2.3. Quality assessment for study bias

A risk of bias assessment was conducted to evaluate the quality and validity of study findings, in accordance with the Critical Appraisal Tools developed by JBI Systematic Reviews.^[20]^ This was assessed independently by 2 reviewers, following the relevant checklist for the study type, including: cohort analyses, cross-sectional studies, economic evaluations, prevalence studies, randomized control trials (RCTs), and systematic reviews. Each of the criteria included in the bias assessment checklists were answered with either “yes,” “no,” “not clear,” or “not applicable (N/A),” and an overall qualitative assessment of low or high risk of bias based on these responses was recorded by the reviewer.

## 3. Results

### 3.1. Summary of results

**Figure 1.**
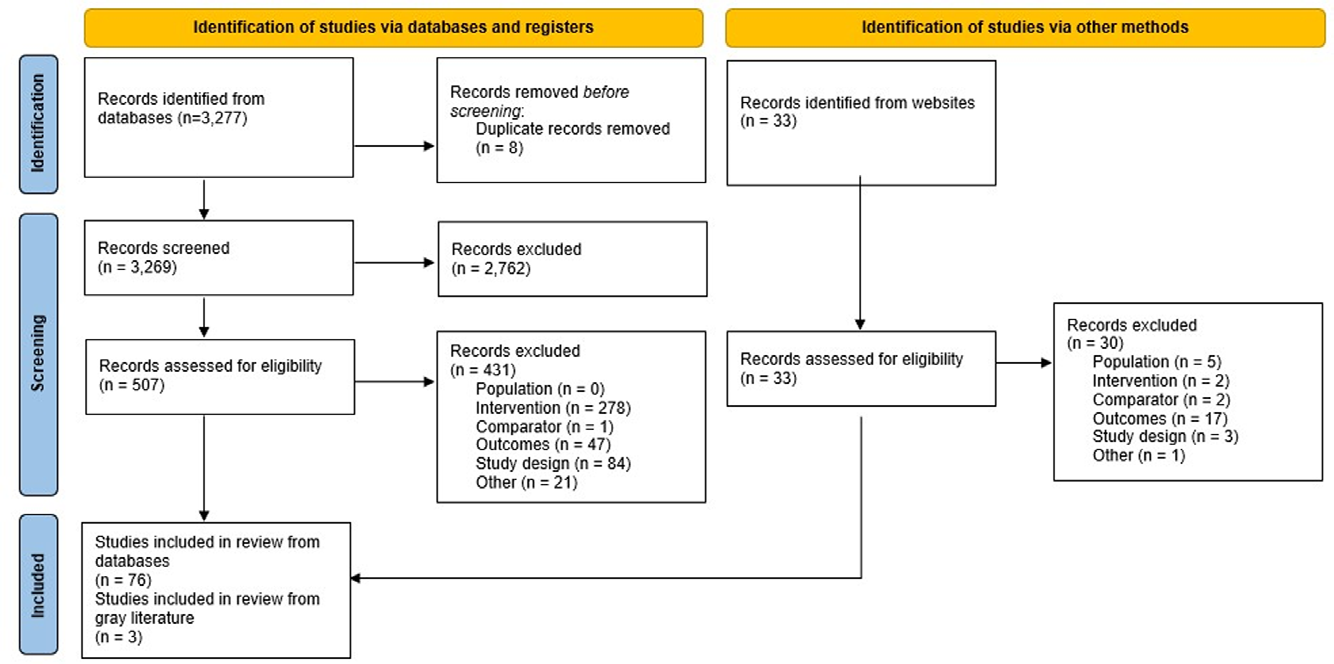
PRISMA flow of included publications.

Of 3269 publications identified in the database and supplementary searches, 79 publications were included in the final analyses, comprising 76 records identified through electronic database search and 3 through gray literature searches. A PRISMA flow of included study publications is presented in Figure 1. Studies excluded at full-text screening are summarized in Table S10, Supplemental Digital Content, http://links.lww.com/MD/N353.

#### 3.1.1. Study characteristics

Of all included studies, clinical outcomes were more frequently reported (N = 58), than economic outcomes (N = 27). Of the economic outcome studies, 7 presented economic burden outcomes and 21 were economic modeling studies. Some studies reported clinical and economic outcomes; therefore, the number of outcomes exceeds the total number of studies. Four studies reported outcomes in pediatric populations, all of which presented clinical outcomes only.

**Figure 2.**
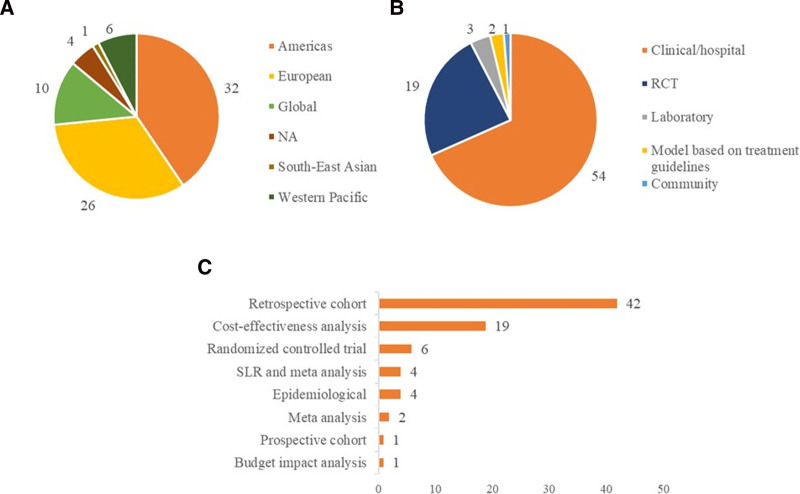
Included study characteristics by (A) WHO region, (B) study setting, and (C) study design. NA = not applicable; RCT = randomized controlled trial; SLR = systematic literature review; WHO = World Health Organization.

Clinical and economic outcomes associated with fidaxomicin were reported across multiple regions. A breakdown by World Health Organization region is presented in Figure 2. Most studies were conducted in the Americas (N = 32), followed by the European region (N = 26). A breakdown of studies by setting and design is also presented in Figure 2. Retrospective cohort (N = 42) and cost-effectiveness analysis (N = 19) were the most common study design.

A summary of included study characteristics is presented in Table S2, Supplemental Digital Content, http://links.lww.com/MD/N353.

### 3.2. Clinical outcomes

The SLR identified 58 studies that reported the clinical outcomes associated with use of fidaxomicin alone or versus vancomycin, metronidazole, or FMT for the treatment of CDI.

There was substantial heterogeneity between studies in outcome definition, the number of included patients, and assessment timepoint. Therefore, to show meaningful ranges of data across studies for the most frequently reported clinical outcomes (clinical cure, recurrence rate, and all-cause mortality), only studies reporting study populations of ≥50 patients over a follow-up period of ≤90 days posttreatment were included in the primary cross-study comparisons (N = 14; Table 2). These limits were selected as larger study populations generate more accurate mean or median values and follow-up periods across studies identified in the SLR fell within this timepoint range, except for a few extreme values (e.g., 1.5 years).

**Table 2:** Summary of studies including study populations of ≥50 patients over a follow-up period of ≤90 days posttreatment (N = 14).

Study details	Intervention	Follow-up period	Study population
Dubberke et al (2022)^[21]^	Fidaxomicin, vancomycin	4 weeks post-initial CDI episode	Elderly fee-for-service Medicare beneficiaries with initial or recurrent CDI episode (N = 380)
Eiland et al (2015)^[22]^	Fidaxomicin	30 days posttreatment initiation	Adults with a CDAD diagnosis, based on symptoms and positive PCR for toxin B (N = 60)
Escudeo-Sanzhez et al (2021)^[23]^	Fidaxomicin	≤12 weeks posttreatment initiation	Patients who had received fidaxomicin for ≥2 days for clinically confirmed CDI (N = 244)
Fehér et al (2017)^[24]^	Fidaxomicin	30-days posttreatment completion	Adult patients with microbiologically documented, symptomatic CDI (N = 72)
Gentry et al (2019)^[25]^	Fidaxomicin, vancomycin	90 days posttreatment	Patients with severe CDI (baseline white blood cell count ≥1.5 times the premorbid level) treated <72 hours of positive toxin result (N = 852)
Guery et al (2017)^[26]^	Fidaxomicin, vancomycin	90-days posttreatment	Hospitalized patients aged ≥60 years with clinically confirmed CDI (N = 356)
Hvas et al (2019)^[27]^	Fidaxomicin, vancomycin, FMT preceded by vancomycin	Week 8 posttreatment completion	Adult patients with rCDI and documented recurrence within 8 weeks after stopping anti-CDI treatment (N = 64)
Mekideche et al (2018)^[28]^	Fidaxomicin	30-days post- treatment	Adult patients with CDI (N = 50)
Mikamo et al (2018)^[29]^	Fidaxomicin, vancomycin	28-days posttreatment completion	Hospitalized patients aged ≥20 years with ≥4 episodes of unformed bowel movements within 24 hours before randomization and *C. difficile* toxin A/B confirmed in stool ≤96 hours prior to randomization (N = 215)
Patel et al (2021)^[30]^	Vancomycin	30-days from date of a toxin-positive stool sample	Adult patients hospitalized for a CDI episode with a Hines Severity Score Index ≥2, treated <5 days prior to positive CDI stool sample for ≥48 hours (N = 54)
Polivkova et al (2021)^[31]^	Fidaxomicin, vancomycin, metronidazole	60-days posttreatment completion	Patients with confirmed CDI (i.e., ≥3 unformed stools and positive laboratory test) (N = 271)
Spiceland et al (2018)^[32]^	Fidaxomicin	4 to 8 weeks posttreatment	Patients with ≥3 episodes of watery diarrhea per 24 hours and *C. difficile* detected in stool either by PCR or enzyme immune assay (N = 81)
Tieu et al (2019)^[33]^	Fidaxomicin, vancomycin	30-days from date of the index positive test for *C. difficile*	Patients aged ≥18 years who had first or second recurrent CDI episode treated with oral fidaxomicin or oral vancomycin (N = 65)
Wilcox et al (2018)^[34]^	Fidaxomicin, vancomycin	90 days posttreatment	Patients aged ≥60 years with CDI confirmed via positive *C. difficile* toxin A/B testing (N = 356)

CDAD = *Clostridioides difficile*-associated diarrhea, CDI = *Clostridioides difficile* infection, PCR = polymerase chain reaction.

### 3.3. Clinical outcomes with study population and follow-up limits applied

**Figure 3.**
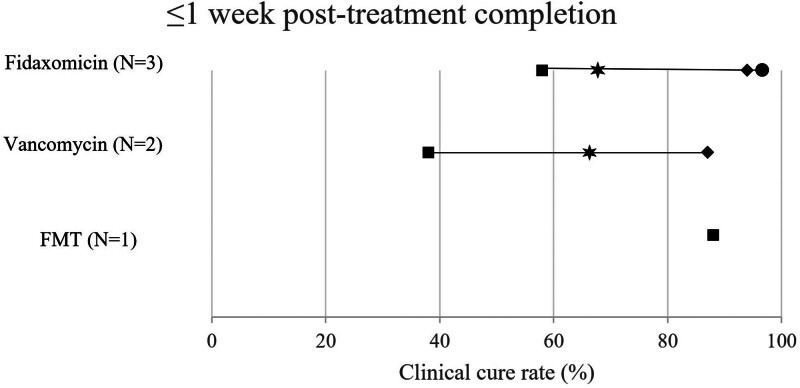
Forest plot for studies reporting clinical cure for 7 days posttreatment completion among studies including ≥50 patients and outcomes recorded at ≤90 days.

**Figure 4.**
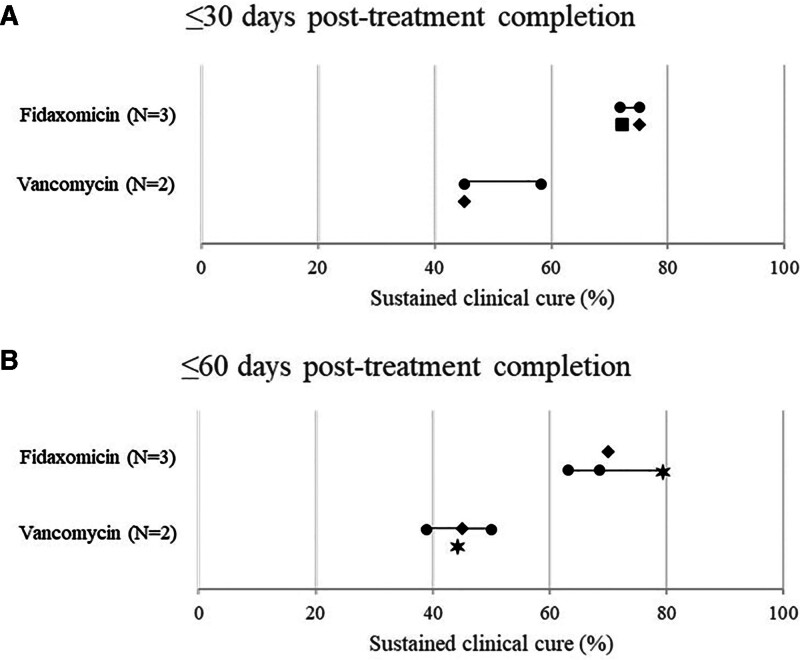
Forest plots for 30-days (A) and 60-days posttreatment completion (B) for sustained clinical cure rate among studies including ≥50 patients and outcomes recorded at ≤90 days.

**Figure 5.**
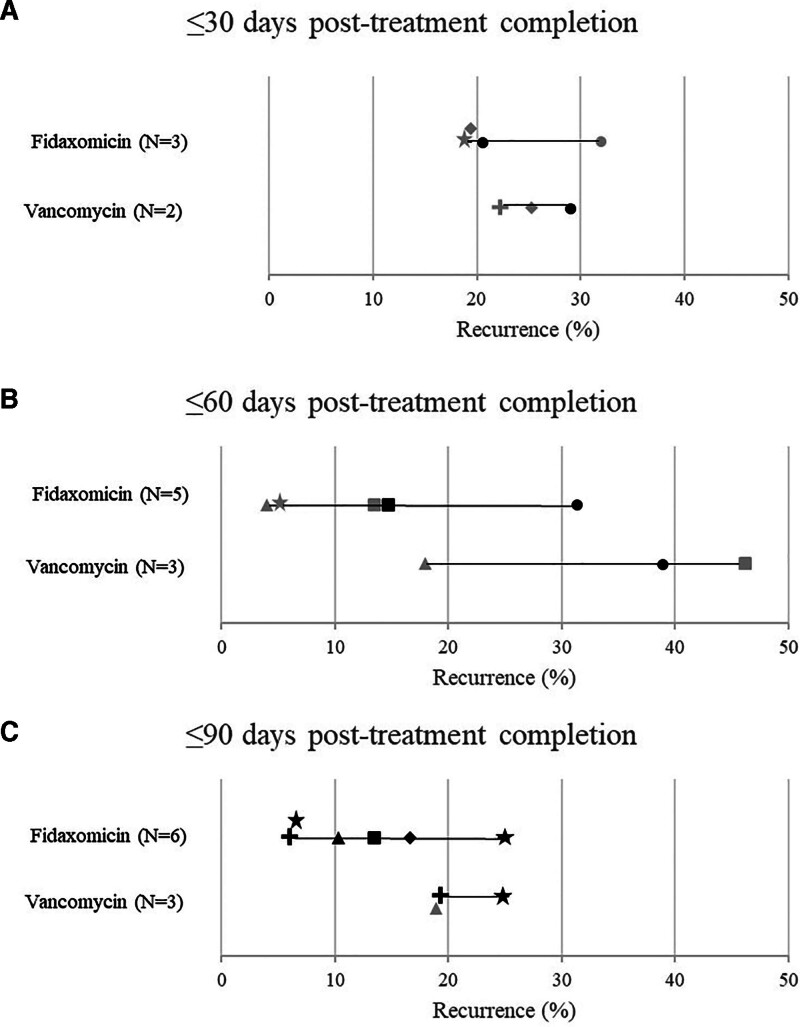
Forest plots for 30-days (A), 60-days (B), and 90 days follow-up posttreatment completion (C) for recurrence rate among studies including ≥50 patients and outcomes recorded at ≤90 days.

Only total study population was considered when selecting the studies to include in the primary analyses, although the number of patients receiving each intervention varied. The number of data points for outcomes presented in Figures 3–5 exceeds the number of studies, as multiple data points relating to total study population were included (e.g., values for full cohort at several timepoints during follow-up within the 90-day limit were each plotted).

#### 3.3.1. Clinical cure and sustained clinical cure

Clinical cure refers to an initial resolution of CDI symptoms posttreatment with no need for further treatment, while sustained clinical cure refers to initial clinical response plus survival for a follow-up period posttreatment without recurrence of CDI. The overall rates of clinical cure with cross-study selection criteria applied (i.e., studies including ≥50 patients and outcomes recorded at ≤90 days) were reported by 4 studies and are presented in Figure 3 and Table 3. A range of follow-up durations were reported within the 90-day limit, with up to 1 week posttreatment completion as the most reported period (Fig. 3).^[35]^

**Table 3:** Study details for clinical cure rate among studies including ≥50 patients and outcomes recorded at ≤90 days.

Marker in figure	Study details	Sample size	Outcome definition	Follow-up period	Clinical cure rate (%)		
					Fidaxomicin	Vancomycin	Vancomycin followed by FMT
✶	Mikamo et al (2018) ^[29]^	203*	Global cure (treated patients cured at EOT with no recurrence)	28 days posttreatment completion	67.3	65.7	–
•	Eiland et al (2015)^[22]^	60	Clinical success (resolution of signs and symptoms of the disease and no further therapy required for CDAD)	2 days posttreatment completion	96.7	–	–
◆	Guery et al (2017)^[26]^	356	Clinical response (per ESC guidelines: stool frequency decreases or improved stool consistency, disease severity parameters improve)	Day 12 post-intervention initiation Day 27 (2 days posttreatment completion)	80 94	82 88	– –
■	Hvas et al (2019)^[27]^	64	Clinical resolution without the need for rescue FMT with vancomycin or colectomy	Week 1 posttreatment completion Week 8 posttreatment completion	58 42	38 19	88 92

CDAD = *Clostridioides difficile*-associated diarrhea, CDI = *Clostridioides difficile* infection, EOT = end of treatment, ESC = European Society of Cardiology, FMT = fecal microbiota transplantation, rCDI = recurrent *Clostridioides difficile* infection.

* Modified full analysis set.

Rate of clinical cure at less than 7 days posttreatment follow-up tended to be higher among adult patients treated with fidaxomicin or extended-pulse (EP)-fidaxomicin (58–96.7%, N = 3),^[22,26,27]^ than vancomycin (38–88.0%, N = 2).^[26,27]^ One study reported rate of clinical cure for FMT preceded by 4 to 10 days of vancomycin 125 mg 4 times daily (88–92%; N = 1).^[27]^

One study including pediatric patients, an international RCT, (fidaxomicin: N = 98, median age: 5 years; vancomycin: N = 44, median age: 4 years), reported a higher rate of clinical cure among patients who received fidaxomicin than vancomycin (77.6% versus 70.5%, adjusted risk difference: 7.5%, [95% confidence interval [CI]: ‐7.4–23.9%]).^[36]^

Rates of sustained clinical cure were reported by 4 studies and are presented in Figure 4 and Table 4. A range of follow-up durations were reported within the 90-day limit, with up to 30- and 60-days posttreatment completion reported by all studies (Fig. 4). Rates of sustained clinical cure were higher at 30-days and 60-days follow-up among adult patients treated with fidaxomicin or EP-fidaxomicin than vancomycin. The ranges did not overlap, indicating that fidaxomicin or EP-fidaxomicin induced superior sustained clinical cure compared to vancomycin. At 30 days, the rate of sustained clinical cure post-fidaxomicin treatment was 70.0% to 75.1% (N = 3),^[21,24,26]^ and post-vancomycin treatment was 45.1% to 58.2% (N = 3).^[21,26]^ Similarly, there was no overlap in the ranges at 60 days, with a rate of 63.2% to 78.9% for fidaxomicin (N = 3),^[21,26,31]^ and 38.9% to 50.0% for vancomycin (N = 3).^[21,26,31]^

**Table 4:** Study details for sustained clinical cure rate among studies including ≥50 patients and outcomes recorded at ≤90 days

Marker in figure	Study details	Sample size	Outcome definition	Follow-up period	Sustained clinical cure rate (%)		
					Fidaxomicin	Vancomycin	Metronidazole
•	Dubberke et al^21^	380	Clinical resolution (i.e., no additional CDI treatment or hospitalization before or within 1 day of EOT) and no evidence of recurrence	Week 4 postinitial episode Week 8 postinitial episode Week 4 postrecurrent episode Week 8 postrecurrent episode	71.7 63.2 75.1 68.5	58.2 50.0 45.1 38.9	– – – –
▪	Fehér et al^24^	72	Clinical cure and recurrence free	30 d post-treatment completion	72.2	–	–
◆	Guery et al^26^	356*	Clinical response at test of cure and no recurrence	Day 30 post-treatment completion Day 40 Day 55 Day 90	70 75 70 66	58.2 50.0 45.1 38.9	– – – –
✶	Polivkova et al^31^	271	Surviving patients with resolution of diarrhea and no recurrence	60 d post-treatment completion	78.9	43.8	49.4

CDI = *Clostridioides difficile* infection, EOT = end of treatment.

* Modified full analysis set.

One study reported the sustained clinical cure rate for adult patients who received metronidazole (49.4%) or a combination of metronidazole and vancomycin (45.5%).^[31]^ This study included patients with confirmed CDI (i.e., ≥3 unformed stools and positive laboratory test, N = 271), with a follow-up period of 60 days posttreatment completion, and found fidaxomicin to be significantly associated with higher rates of sustained clinical cure than metronidazole alone (% difference: 29.5%, odds ratio [OR]: 3.8; 95% CI: 1.8–8.4, *P* = .0007) and in combination with vancomycin (% difference: 33.4%, OR: 4.5; 95% CI: 2.0–10.3, *P* = .0004).^[31]^

No studies reported sustained clinical cure among pediatric populations.

#### 3.3.2. Recurrence

Rates of CDI recurrence were reported in 12 studies and are presented in Figure 5 and Table 5.

**Table 5:** Study details for recurrence rate among studies including ≥50 patients and outcomes recorded at ≤90 days.

Marker in figure	Study details	Sample size	Outcome definition	Follow-up period	Recurrence rate (%)		
					Fidaxomicin	Vancomycin	Metronidazole
•	Dubberke et al (2022)^[21]^	380	CDI treatment or hospitalization post-clinical resolution	4 weeks post-clinical resolution 8 weeks post-clinical resolution	20.6 31.3	29.0 38.9	– –
▲	Eiland et al (2015) ^[22]^	60	Reappearance of symptoms posttreatment, positive CDI test and retreatment	90-days posttreatment completion	10.3	–	–
▪	Escudero-Sanchez et al (2021)^[23]^	244	Reappearance of disease symptoms with a post- resolution of symptoms	≤8 weeks ≤12 weeks	14.7 13.5	– –	– –
◆	Fehér et al (2017)^[24]^	72	Reappearance of microbiologically documented clinical CDI	≤12 weeks posttreatment initiation	16.7	–	–
★	Gentry et al (2019)^[25]^	852	Positive toxin test result plus evidence of CDI antimicrobial treatment	90-days posttreatment completion	24.4	24.4	–
▲	Guery et al (2017)^[26]^,*	356	Return of diarrhea post-clinical response	Day 40 Day 55 Day 90	2 4 6	17 18 19	– – –
•	Mekideche et al (2018)^[28]^	50	Return of diarrhea posttreatment completion	30-days posttreatment	32	–	–
◆	Mikamo et al (2018)^[29]^	215	Increased frequency of unformed bowel, positive stool test and need for retreatment	28-days post- treatment	19.5	25.3	–
✚	Patel et al (2021)^[30]^	54	Patients hospitalized with CDI with a positive CDI stool sample for ≥ 48 hours	28-days posttreatment completion	–	22	–
▪	Polivkova et al (2021)^[31]^	271	Surviving patients presenting with symptoms and a positive laboratory test for CDI	60-days posttreatment completion	13.5	46.2	41.8
★	Spiceland et al (2018)^[32]^	81	≥3 episodes of watery diarrhea per 24 hours and *C. difficile* detected in stool either by PCR or enzyme immune assay after documented symptom resolution	<4 weeks 4–8 weeks ≤8 weeks	19 14 5	– – –	– – –
✚	Wilcox et al (2018)^[34]^	356	Diarrhea recurring and positive toxin test	90-days posttreatment	6	19	–

CDAD = *Clostridioides difficile*-associated diarrhea, CDI = *Clostridioides difficile* infection, EOT = end of treatment, PCR = polymerase chain reaction.

* The number of data points for outcomes presented in part (A) exceeds the number of studies, as multiple data points relating to total study population were considered.

Across 30-, 60-, and 90-days follow-up, rate of recurrence tended to be lower among adult patients treated with fidaxomicin or EP-fidaxomicin than vancomycin, although the ranges did overlap. Rate of recurrence post-fidaxomicin ranged from 19% to 32% at 30-days (N = 4),^[21,28,29,32]^ 4% to 31.3% at 60-days (N = 5),^[21,23,26,31,32]^ and 6% to 24.4% at 90-days follow-up (N = 6).^[22–24,26,32,34,37]^ Rate of recurrence post-vancomycin treatment ranged from 22% to 29% at 30-days (N = 3),^[21,29,30]^ 18% to 46.2% at 60-days (N = 3),^[21,26,31]^ and 19 to 24.4 at 90-days follow-up (N = 3).^[25,26,34]^

One study reported the recurrence rate among adult patients with confirmed CDI (i.e., ≥3 unformed stools and positive laboratory test, N = 271), who were followed up for 60 days posttreatment completion and received metronidazole (41.8%) or a combination of metronidazole and vancomycin (35.9%).^[31]^ In this study, fidaxomicin was significantly associated with lower rates of recurrence CDI than metronidazole alone (% difference: ‐28.3%, OR: 0.2; 95% CI: 0.1–0.6, *P* = .00013) and in combination with vancomycin (% difference: ‐32.7%, OR: 0.2; 95% CI: 0.1–0.5, *P* = .0003).^[31]^

One study including a pediatric population met the parameters, an international RCT (fidaxomicin: N = 98, median age: 5 years; vancomycin: N = 44, median age: 4 years) defining recurrence as the return of diarrhea post-clinical, plus a positive stool test for toxigenic *C. difficile,* and requiring retreatment with CDAD anti-infective therapy.^[38]^ The study reported an overall lower rate of recurrence among patients receiving fidaxomicin than vancomycin (11.8% vs 29.0%, adjusted risk difference: ‐15.8%, 95% CI: ‐34.5–0.5).^[36]^

#### 3.3.3. Mortality outcomes

Rate of all-cause mortality with cross-study parameters applied (i.e., studies including ≥50 patients and outcomes recorded at ≤90 days) was reported by 6 studies (presented in Table 6). There was variation in follow-up period duration and point of index (i.e., posttreatment initiation versus completion).

**Table 6:** All-cause mortality rate among studies including ≥50 patients and outcomes recorded at ≤90 days.

Study details	Sample size	Follow-up period	All-cause mortality rate (%)		
			Fidaxomicin	Vancomycin	Metronidazole
Escudero-Sanchez et al (2021)^[23]^	244	≤8 weeks ≤12 weeks	7.1 11.8	– –	– –
Fehér et al (2017)^[24]^	72	30-days posttreatment completion	4.2	–	–
Gentry et al (2019)^[25]^	852	30 days posttreatment 90 days posttreatment	10.8 22.5	11.7 21.9	– –
Patel et al (2021)^[30]^	54	30-days from date of a positive toxin test 60-days from date of a positive toxin test	– –	31.5 38.9	– –
Polivkova et al (2021)^[31]^	271	60-days posttreatment completion	8.7	18.8	15.2
Tieu et al (2019)^[33]^	65	30-days from date of the index positive test	0	4.1	–

CDI = Clostridioides difficile infection.

Rate of all-cause mortality post-fidaxomicin treatment ranged from 4.2% to 10.8% at 30-days (N = 2),^[24,37]^ 7.1% to 8.7% at 60-days (N = 2),^[23,31]^ and 11.8% to 22.5% at 90-days (N = 2).^[23,37]^ Post-vancomycin treatment all-cause mortality rate was less frequently reported, with 11.7% at 30-days (N = 1), 18.8% at 60-days (N = 1), and 21.9% at 90-days (N = 1).

One study reported all-cause mortality among 271 patients at 60 days posttreatment completion who had received metronidazole alone or in combination with vancomycin. All-cause mortality rate among patients receiving metronidazole alone was 15.2%, and 29.1% among patients receiving combination therapy of metronidazole and vancomycin.^[31]^ No studies reported mortality among pediatric populations.

### 3.4. Clinical outcomes in studies not meeting sample size and follow-up parameters

Of the studies included in the SLR, 39 reported outcomes recorded with <50 patients or at >90 days posttreatment.

Clinical cure within adult populations ranged from 39% to 100% among studies reporting fidaxomicin treatment (N = 7),^[39–45]^ and 77.8% to 96.4% for vancomycin treatment (N = 5).^[41–45]^ Sustained clinical cure at any timepoint within adult populations ranged from 58.9% to 100% in studies reporting fidaxomicin treatment (N = 3) and 51.4% to 84.6% for vancomycin (N = 3).^[42,43,46]^ One study including pediatric patients reported a sustained clinical cure rate for fidaxomicin of 22.2% to 37.5% (across age subgroups from 6–23 months to 12–<18 years).^[47]^ Recurrence at any timepoint within adult populations ranged from 0% to 47.8% in studies reporting fidaxomicin treatment (N = 11),^[40,42–44,46,48–53]^ and from 10.3% to 77.6% for vancomycin treatment (N = 9).^[42–44,46,48–51,53]^ Two studies including pediatric populations reported clinical cure and recurrence post-fidaxomicin treatment, which ranged from 30.4% to 92.1% and 0% to 77.8%, respectively.^[47,54]^ All-cause mortality rate among adults receiving fidaxomicin treatment ranged from 3.2% to 22.0% (N = 3),^[52,53,55]^ with 1 study reporting a mortality rate of 4% among vancomycin-treated patients.^[53]^ Two studies reporting CDI-related mortality were identified, both for patients who had received fidaxomicin without comparator (Table 7). CDI-related mortality ranged from 10% to 12.5%.^[56,57]^

**Table 7:** CDI-related mortality (N = 2).

Study details	Sample size	Follow-up period	Mortality rate N (%)
			Fidaxomicin
Enoch et al (2018)^[56]^	16	On days 1 and 5 posttreatment initiation	2 (12.5)
Novotný et al (2018)^[57]^	60	Not reported	6 (10)*

CDI = Clostridioides difficile infection.

* Of the 6 patients (37.5%) who received fidaxomicin for a first recurrence 4 were clinically suspected CDI and 2 were microbiologically confirmed, while 8 patients (50%) received fidaxomicin for a second recurrence - of whom 5 were clinically suspected and 3 microbiologically confirmed). One patient (5.3%) was treated for a third microbiologically confirmed recurrence.

Seven studies were not included in the pooled ranges because they were SLRs or meta-analyses (MA; Table 8).^[^^48,58–63]^ Generally studies reporting recurrence rate found fidaxomicin to be associated with a significantly lower risk of recurrence: four^[41,59–61]^out of five^[41,58–61]^ studies versus vancomycin and two out of two studies versus metronidazole.^[^^41,61]^ Clinical or symptomatic cure post-fidaxomicin treatment was superior to vancomycin, overall and in the majority of subgroups, in 2 studies,^[60,63]^ and metronidazole in 2 studies.^[61,63]^ However, the remaining 4 studies reported comparable clinical cure rates between fidaxomicin- and vancomycin-treated cohorts,^[41,58,61,62]^ and 1 study found donor FMT to be superior to both.^[62]^

**Table 8:** Summary of included SLRs/MAs (N = 7)

References	Population/intervention detail
Cornely et al^[41]^	Population: Patients with CDI who received fidaxomicin or standard of care (vancomycin or metronidazole) treatment (N=1509 [5 studies]) Intervention: Fidaxomicin, vancomycin, metronidazole (dose NR)
Dai et al^[58]^	Population: CDI patients requiring antibiotic therapy (N=2151 across 10 studies), mean age range: 47.5–74.6 Intervention: Fidaxomicin (200 mg twice daily [dose reported by 2/10 studies]), vancomycin (125 mg 4 times daily [reported by 4/10 studies])
Liao et al^[59]^	Population: Adults who have received CDI treatment and experienced recurrence (N=3944 across 14 studies), mean age range: 46–75 Intervention: Fidaxomicin (200 mg [9/14 studies reported] twice daily, 7–10 d, median 11–14 d), vancomycin (125–500 mg [11/14 studies reported] 4 times daily, median 11–14 d)
Tashiro et al^[60]^	Population: Patients aged ≥16 with confirmed CDI (N=NR, 6 studies in total) Intervention: Fidaxomicin (200 mg twice daily for 10 d), vancomycin (125 mg 4 times daily for 10 d)
Okumura et al^[61]^	Population: Adult patients with confirmed CDI of any severity (N=NR, included 7 studies) Intervention: Fidaxomicin (200 mg twice daily for 10 d), vancomycin (125 mg/500 mg 4 times daily for 10 d/3 times daily for 10 d), metronidazole (500 mg 3 times daily for 10 d)
Rokkas et al ^[62]^	Population: Participants who experienced resolution of CDI-related symptoms (N=348 across 6 studies) Intervention: Fidaxomicin, vancomycin, donor FMT, placebo, autologous FMT
Sridharan et al^[63]^	Population: Patients participating in 11 RCTs comparing antimicrobials for CDI (N=2888 across 17 studies) Intervention: Fidaxomicin (200 mg twice a day for 10 d), vancomycin (125 mg 4 times daily or 500 mg thrice daily for 10 d), metronidazole (500 mg thrice daily with rifampicin, 250 or 375 mg 4 times daily or 400 mg thrice daily)

CDI = *Clostridioides difficile* infection, FMT = fecal microbiota transplantation, MA = meta-analysis, NR = not reported, OR = odds ratio, rCDI = recurrent *Clostridioides difficile* infection, RCT = randomized controlled trial, SLR = systematic literature review.

Outcomes other than clinical cure, recurrence, and mortality, were primarily studies reporting antibiotic susceptibility of fidaxomicin, vancomycin, and metronidazole (N = 8, see Table S2, Supplemental Digital Content, http://links.lww.com/MD/N353).^[64–71]^

### 3.5. Economic outcomes

The SLR identified 7 studies reporting costs and HCRU associated with fidaxomicin use versus vancomycin, metronidazole or FMT,^[22,51,52,72–75]^ and 21 economic modeling studies.^[72,76–95]^

#### 3.5.1. Costs and HCRU

Two studies identified in the SLR reported direct costs in patients with CDI.^[51,72]^ McDaniel et al (2022) compared total treatment costs between pre- and post-implementation of fidaxomicin as first-line treatment for patients experiencing their first and second CDI episode, reporting significant savings in the post-implementation period (difference: ‐$2588.63, *P* = .048; adjusted to 2020 US Dollars).^[72]^ Similarly, Gallagher et al (2015) reported that total drug, hospital readmission, and readmission reimbursement costs were $3047 lower per patient for patients treated with fidaxomicin than for patients who received vancomycin.^[40]^ In this study, there was a comparable hospital length of stay between the fidaxomicin and vancomycin groups (mean days: 8.96 vs 10.6, *P* = .26), while CDI-related hospital readmission was significantly less frequent among fidaxomicin-treated patients (20.4% vs 41.3%, *P* = .027), leading to overall hospital savings despite the higher drug cost associated with fidaxomicin.^[40]^ None of the studies identified in the SLR reported indirect costs associated with fidaxomicin use.

#### 3.5.2. Economic analyses

Of the 21 economic analyses identified in the SLR, fidaxomicin or EP-fidaxomicin were reported to be cost-effective either overall or in patient subgroups in 11 studies when compared to vancomycin,^[77,78,80–82,86,87,89,91,92,94]^ in 2 studies when compared to metronidazole,^[81,92]^ and in 1 study the most effective strategy was the use of fidaxomicin for non-severe initial CDI, vancomycin for severe CDI, and FMT for first and subsequent recurrences.^[76]^ In addition, 3 studies reported fidaxomicin as cost-saving versus vancomycin.^[84,93,95]^ Three studies reported FMT to be more cost-effective than fidaxomicin, although neither study included high-risk patient subgroups, such as patients with comorbidities or patients receiving concomitant treatment.^[79,85,90]^ Two studies reported fidaxomicin to be more effective but more costly than vancomycin or metronidazole.^[83,88]^ Finally, 1 study reported a positive association between increased fidaxomicin uptake, improved clinical outcomes and hospital cost-savings.^[72]^

An overview of economic analyses included in this SLR is presented in Table 9, with a detailed summary including model design, intervention regimen, and key inputs presented in Tables S3 and S4, Supplemental Digital Content, http://links.lww.com/MD/N353.

**Table 9:** Overview of economic analysis key findings by study type (N = 21).

Study	Population detail	Key findings	Favored intervention	Study type
*Budget impact analysis*				
Jiang et al (2022)^[44]^	Initial/rCDI	Cost savings: - no prior CDI episode, hospital level: $7824, per treated patient: $96; - 1 prior CDI episode, hospital level: ‐$3725, per treated patient ‐$238 for fidaxomicin vs vancomycin	Fidaxomicin	BIM
McDaniel et al (2022)^[72]^	Initial CDI	Overall direct cost savings: $222,895 per 100 patients for fidaxomicin	Fidaxomicin	BIM
*Cost effectiveness analysis*				
Abdali et al (2020)^[79]^	rCDI	Fidaxomicin dominated by FMT administered both by colonoscopy and by nasogastric tube (NGT; fidaxomicin resulted in a loss of 0.068 quality adjusted life years [QALYs]) compared to FMT-NGT	FMT	CEA
Aby et al (2022)^[76]^	Initial/rCDI	The most effective strategy was the use of fidaxomicin for non-severe initial CDI, vancomycin for severe CDI, and FMT via colonoscopy (with an oral vancomycin course preceding FMT) for first and subsequent recurrences (incremental cost-effectiveness ratio (ICER): $27,135/QALY vs the next less expensive strategy that is the use of vancomycin for initial non-severe CDI as well as for severe initial CDI and FMT for all recurrences of CDI)	Fidaxomicin for non- severe initial CDI, vancomycin for severe CDI, FMT for recurrences	CEA
Brodsky et al (2014) ^[94]^	Initial CDI	ICER: €5520/QALY for fidaxomicin vs vancomycin	Fidaxomicin	CEA
Chen et al (2021)^[78]^	Initial CDI	ICER: $495/QALY for fidaxomicin vs vancomycin	Fidaxomicin	CEA
Cornely et al (2018)^[95]^	Initial CDI	ICER: £33,327/QALY for EP-fidaxomicin at Days 0 to 5 vs vancomycin, £32,833/QALY for EP fidaxomicin at Days 5 to 10 vs vancomycin	Fidaxomicin	CEA
Ford et al (2018)^[83]^	Initial moderate-severe CDI	ICER: $2828.69/1% gain in cure for fidaxomicin vs vancomycin resulting in an incremental cost of over $291,467 for each additional patient cured from a hospital perspective. ICER of $1540.23/1% gain in cure for fidaxomicin vs metronidazole	Vancomycin	CEA
Konijeti et al (2014)^[90]^	rCDI	Fidaxomicin more expensive and less effective than FMT, dominated by FMT with recurrent CDI. In clinical settings where FMT is not available or applicable, the preferred strategy appeared to be initial treatment with oral vancomycin.	FMT	CEA
Lapointe-Shaw et al (2016)^[85]^	Initial/rCDI	FMT by colonoscopy or enema after a 2-week course of oral vancomycin dominated fidaxomicin. If FMT by any means was unavailable, fidaxomicin was cost-effective (ICER: $25,968/QALY for fidaxomicin vs metronidazole)	FMT	CEA
Markovic et al (2014)^[91]^	Initial CDI	ICER: $2977,621.51/QALY for fidaxomicin vs vancomycin	Vancomycin	CEA
Nathwani et al (2014)^[89]^	rCDI/severe CDI	ICER: $16,529/QALY for fidaxomicin vs vancomycin	Fidaxomicin	CEA
Okumura et al (2020)^[80]^	Initial/rCDI	ICER: 5715,183 Japanese Yen (JPY)/QALY for fidaxomicin vs vancomycin	Fidaxomicin	CEA
Rajasingham et al (2020)^[81]^	Initial CDI, non-severe	ICER: $31,751/QALY for fidaxomicin vs vancomycin	Fidaxomicin	CEA
Reveles et al (2017)^[84]^	Initial CDI, comorbidities	Fidaxomicin resulted in total hospital cost savings of $616 and $312 per patient for cancer and concomitant antibiotic use subgroups, respectively, vs vancomycin	Fidaxomicin	CEA
Rubio-Terrés et al (2015)^[87]^	Initial CDI, comorbidities	Fidaxomicin associated with +0.016 QALYs and cost saving of €2397 per patient vs vancomycin	Fidaxomicin	CEA
Rubio-Terrés et al (2019)^[82]^	Initial CDI	Extended pulsed (EP)-fidaxomicin associated with +0.044 QALYs and cost saving of €647 per patient vs vancomycin	Fidaxomicin	CEA
Sclar et al (2012)^[93]^	Initial CDI diarrhea	From hospital perspective: loss of $56.37 per day vs injectable vancomycin, savings of $43.64 to $133.63 per day vs vancomycin capsules	Fidaxomicin	CEA
Stranges et al (2013)^[92]^	Mild-moderate CDI	ICER: $32,020/QALY for fidaxomicin vs vancomycin	Fidaxomicin	CEA
Wagner et al (2014)^[88]^	Initial/rCDI	Fidaxomicin associated with total incremental cost of $1.81 million vs vancomycin in a cohort of 1000 patients, vancomycin more cost-effective	Vancomycin	CEA
Watt et al (2016)^[86]^	Initial/rCDI	ICER: €26,900 to €44,500/QALY across patient subgroups vs vancomycin	Fidaxomicin	CEA

BIM = budget impact model, CDI = *Clostridioides difficile* infection, CEA = cost effectiveness analysis, EP = extended pulsed, FMT = fecal microbiota transplantation, ICER = incremental cost-effectiveness ratio, NGT = nasogastric tube, QALY = quality adjusted life-year, rCDI = recurrent *Clostridioides difficile* infection.

In studies that reported fidaxomicin as the most cost-effective treatment option, key inputs tended to be clinical cure rate, sustained cure rate, recurrence rate, and hospitalization costs, as demonstrated through sensitivity analyses.^[77,78,92]^ Fewer recurrent episodes and outcomes associated with recurrence (e.g., hospital readmission costs) plus a higher proportion of patients achieving clinical cure were reported to offset the higher acquisition cost of fidaxomicin across 10 studies.^[72,77,80,81,84,86,89,92,94,95]^

In studies reporting findings in specific high-risk patient subgroups, fidaxomicin was consistently cost-effective or cost-saving versus vancomycin among patients receiving concomitant antibiotics,^[80,84,86,92]^ patients with cancer,^[84,86,87]^ and patients with renal impairment.^[86,87]^ No comparison with these high-risk subgroups was identified in the SLR for metronidazole or FMT.

### 3.6. Quality assessment

Critical appraisal of included studies was conducted using JBI checklists and the findings from this are presented in Tables S5 to S9, Supplemental Digital Content, http://links.lww.com/MD/N353. Responses to each of the criteria included in the bias assessment checklists were recorded as either: “yes,” “no,” “not clear,” or “NA (N/A),” and an overall qualitative assessment of low or high-risk of bias based on these responses was recorded by the reviewer.

Overall, 13 studies were assessed to be at high-risk of bias, including 5 cohort studies, 6 RCTs, 1 economic evaluation, and 1 systematic review. These studies were primarily conference abstracts which presented limited description of study population and methods (N = 9). For RCT assessment the JBI checklist includes whether participants, those delivering the treatment, and assessors, were blinded to treatment allocation. Therefore, the 4 full-text RCTs studies deemed to be at high-risk of bias were due to open-label design.

## 4. Discussion

This SLR explored clinical and economic outcomes associated with fidaxomicin use versus placebo or comparators vancomycin, metronidazole, or FMT. To account for substantial study population and outcome heterogeneity across studies included in the SLR, studies reporting populations of ≥50 patients over a follow-up period of ≤90 days posttreatment were analyzed independently of those that investigated a specific subpopulation, had a small population size, had outlier follow-up periods or did not specify timepoint. In total, 14 studies met these criteria. These criteria were included to facilitate more meaningful cross-study comparison between fidaxomicin and comparators, by mitigating the impact of outlying data points on clinical outcome ranges deriving from significantly longer follow-up periods and small study populations.

Overall, data from studies that met the aforementioned criteria demonstrated that the effectiveness of fidaxomicin was superior to vancomycin in sustained clinical cure at 30-, 60-, and 90-day follow-up, and comparable to vancomycin in clinical cure across all follow-up periods. While ranges overlapped across all follow-up periods, recurrence rates for fidaxomicin were clustered in the lower end of the range and the overlap was created by a single study. In addition, in all studies with head-to-head comparisons between fidaxomicin and vancomycin with the exception of one,^[25]^ the rate of recurrence was lower in the fidaxomicin-treated patients.^[21,26,29,31,34]^

Further, fidaxomicin was associated with lower all-cause mortality at 30- and 60-day follow-up, although only 1 or 2 studies were considered in these analyses due to few comparable follow-up periods.

Seven SLRs/MAs were identified in the SLR, primarily to cross-check the bibliographies with studies included in this SLR, and ensure all relevant studies were captured via electronic database and supplementary searches. These were not included as primary data in the clinical analyses. However, findings from the published SLRs/MAs supported the findings of this SLR. Of the included SLR/MAs, fidaxomicin was significantly superior to vancomycin in clinical cure in 1 study overall,^[63]^ and in another study among patients with non-severe CDI, initial CDI, non-concomitant antibiotic use, and patients aged < 65 years.^[60]^ Clinical cure post-fidaxomicin was comparable (i.e., OR 95% CI ranges including 1) to vancomycin in another 4 studies.^[41,58,61,62]^ Further, 4 of 5 studies reported significantly lower risk of recurrence (i.e., OR 95% CI upper bound < 1) associated with fidaxomicin treatment compared to vancomycin.^[41,59–61]^

Twenty-one economic analyses were included in this study, of which 13 studies reported fidaxomicin to be cost-effective or cost-saving primarily compared to vancomycin, but also to metronidazole. Fidaxomicin was consistently cost-effective versus vancomycin among specific high-risk subpopulations including patients with cancer or renal impairment and patients with concomitant antibiotic use.^[80,84,86,92]^ Key inputs included in studies where fidaxomicin was the most cost-effective treatment option were sustained cure rate, recurrence rate, and hospitalization costs, as demonstrated through sensitivity analyses.^[77,78,92]^

Reducing CDI recurrence is a key component in the cost-effectiveness of a treatment for CDI, as repeat recurrences result in frequent ED and outpatient visits each year and thus increase HCRU.^[96]^ A key retrospective study demonstrated the real-world value of implementing a fidaxomicin treatment regimen in the US and showed favorable clinical outcomes in terms of high cure and response rates and lower recurrence rates versus vancomycin.^[72]^ This study additionally demonstrated substantial reductions in costs post-implementation of fidaxomicin, resulting in savings of $222,895 overall and $9432 per CDI-readmission avoided.^[72]^ The demonstrated clinical and cost-effectiveness of fidaxomicin is of particular importance in high-risk populations who are more likely to experience adverse events and require higher HCRU.^[96]^ These populations may derive a substantial benefit from treatment with fidaxomicin. Some economic models, such as those by Abdali et al (2020) and Lapointe-Shaw et al (2016) conversely reported that FMT via nasogastric tube after a 2-week course of oral vancomycin was more cost-effective than fidaxomicin for rCDI. However, neither of these studies included patients with comorbidities or patients receiving concomitant treatment.^[79,85]^ Abdali et al recognized this factor as a limitation of the model, particularly among a study cohort aged ≥65 years who are highly likely to be receiving additional medications that may impact CDI treatment.^[79]^

However, despite these clinical benefits and the recommendation for use for initial and recurrent CDI by the IDSA/SHEA guidelines, fidaxomicin uptake remains low.^[72]^ This may be due to the higher acquisition cost of fidaxomicin, despite evidence that the associated clinical benefits can offset this cost.^[72,77,80,81,84,86,89,92,94,95]^ Increased awareness of the demonstrated cost-effectiveness of fidaxomicin may optimize decision-making and help alleviate the clinical and economic burden associated with CDI.^[72]^

Finally, this review captured data on the clinical benefit of fidaxomicin in pediatric populations, demonstrating effectiveness in achieving clinical cure and preventing recurrence.^[36]^ This evidence, coupled with the updated indication of fidaxomicin to include children aged 6 months and older,^[19]^ may aid in increasing uptake among children to alleviate the burden of CDI. However further research into clinical and economic outcomes associated with fidaxomicin use is needed among pediatric populations.

As a meta-analysis was not conducted, heterogeneity across outcome definition and timepoint of recording may have introduced biases into cross-study comparisons. The definition of recurrence, for example, varied across studies, with differing requirements for 1 or multiple positive stool culture tests, specification regarding reinitiation of CDI treatment, and the presence/return of general or specific CDI symptoms such as diarrhea. Variation was also observed in follow-up duration, timepoint at which outcomes were reported, and study populations (including number of participants and participant characteristics). Therefore, a relatively low number of studies reporting an outcome across comparable parameters (e.g., outcome definition, timepoint) was deemed to be eligible for pooled analyses. When summarizing clinical outcomes across studies, the impact of restrictions of sample size and timepoint on the overall findings was explored. The range of values across the same clinical outcomes among studies that did not meet the restriction criteria demonstrated that the application of these limits did not impact the trends, rather led to more conservative estimations.

## 5. Conclusion

Overall, this SLR supported the clinical and economic value of fidaxomicin versus vancomycin. Of significance, reduced recurrence rate and associated HCRU were shown to offset the higher acquisition cost of fidaxomicin across a range of economic modeling scenarios. With the addition of a pediatric indication for fidaxomicin, greater research into real-world outcomes with fidaxomicin is needed for this population to further elucidate the benefits in a real-world setting. The findings of this study suggest that an increase in fidaxomicin uptake may improve clinical and economic outcomes and alleviate the burden associated with CDI.

## Author contributions

**Conceptualization:** Qinghua Li, Engels Obi, Anne Marciniak, Rebecca Newman.

**Investigation:** Anne Marciniak, Rebecca Newman, Isabelle Whittle, Jason Kufakwaro.

**Methodology:** Qinghua Li, Engels Obi, Anne Marciniak, Rebecca Newman.

**Writing – review & editing:** Qinghua Li, Engels Obi, Anne Marciniak, Rebecca Newman, Isabelle Whittle, Jason Kufakwaro.

**Writing – original draft:** Qinghua Li, Engels Obi, Anne Marciniak, Rebecca Newman, Isabelle Whittle, Jason Kufakwaro.

## Supplementary Material


