# Supplementary material for: Clinical and economic outcomes associated with fidaxomicin in comparison to vancomycin, metronidazole, and FMT: A systematic literature review
Source: Medicine (Baltimore). 2024 Dec 27;103(52):e39219. doi: 10.1097/MD.0000000000039219 (PMC11688082; doi:10.1097/MD.0000000000039219)
Supplement: Supplementary file 1 [file medi-103-e39219-s001.docx]

Table 1. Systematic literature review OVID^®^ database search strategy

| # | Query | Results from 6 Dec 2022 |
| --- | --- | --- |
| 1 | *clostridium difficile infection/ | 17,233 |
| 2 | *clostridioides difficile/ | 10,134 |
| 3 | clostridium difficile.tw. | 36,309 |
| 4 | (c diff* or cdi or cdad).tw. | 40,763 |
| 5 | clostridioides.tw. | 6,493 |
| 6 | or/1-5 | 61,418 |
| 7 | *recurrence risk/ | 6,653 |
| 8 | *hospital discharge/ | 14,511 |
| 9 | *mortality/ | 118,079 |
| 10 | *hospitalization/ | 83,316 |
| 11 | *antibiotic resistance/ | 74,828 |
| 12 | *treatment response/ | 23,172 |
| 13 | *treatment response time/ | 225 |
| 14 | *comorbidity/ | 25,439 |
| 15 | (recurren* or discharge* or respon* or resol* or unrespon* or unresol* or nonrespon* or nonresol* or cure* or resistan* or death* or die* or mortalit*).tw. | 18,954,749 |
| 16 | (or/7-15) not 14 | 19,010,746 |
| 17 | 6 and 16 | 31,059 |
| 18 | *health economics/ | 11,091 |
| 19 | *cost of illness/ | 20,165 |
| 20 | *cost control/ | 10,087 |
| 21 | *disease burden/ | 21,110 |
| 22 | *medical leave/ | 2,241 |
| 23 | *healthcare financing/ | 4,616 |
| 24 | *medical fee/ | 7,111 |
| 25 | *work disability/ | 1,923 |
| 26 | *absenteeism/ | 8,805 |
| 27 | *presenteeism/ | 833 |
| 28 | *productivity/ | 13,288 |
| 29 | *caregiver/ | 58,513 |
| 30 | *length of stay/ | 26,293 |
| 31 | *intensive care/ | 95,269 |
| 32 | *hospitalization cost/ | 1,291 |
| 33 | *health care utilization/ | 54,461 |
| 34 | *hospital readmission/ | 27,474 |
| 35 | ((economic or societ* or socioeconomic or socio economic or illness or disease or patient* or caregiver* or carer* or productiv* or employ*) adj3 (burden or impact or consequence?)).tw. | 443,010 |
| 36 | (productivity or cost* or price* or pricing or pharmacoeconomic* or pharmaco-economic* or expenditure* or expens* or financ* or visit* or inpatient* or outpatient* or hospitali* or readmi* or re admi* or financ* or (length adj5 stay)).tw. | 5,154,768 |
| 37 | (resource* adj2 ("use" or usage or utili* or allocat*)).tw. | 138,641 |
| 38 | (leave adj2 (medical or sick or disability)).tw. | 15,960 |
| 39 | or/18-38 | 5,694,164 |
| 40 | 6 and 39 | 14,955 |
| 41 | *economic evaluation/ | 16,220 |
| 42 | *cost effectiveness analysis/ | 44,668 |
| 43 | *cost utility analysis/ | 13,084 |
| 44 | *cost benefit analysis/ | 20,779 |
| 45 | *cost minimization analysis/ | 8,247 |
| 46 | *economic model/ | 4,673 |
| 47 | *decision tree/ | 4,290 |
| 48 | *markov chain/ | 3,630 |
| 49 | *quality adjusted life year/ | 4,390 |
| 50 | (economic adj2 (analy* or evaluat*)).tw. | 76,174 |
| 51 | (cost adj2 (effective* or utilit* or minimi* or benefit)).tw. | 465,102 |
| 52 | (model or markov or (decision adj2 tree)).tw. | 5,977,813 |
| 53 | (incremental cost effectiveness ratio or icer).tw. | 28,692 |
| 54 | budget impact.tw. | 7,194 |
| 55 | (quality adjusted life year or qaly?).tw. | 48,487 |
| 56 | (life Years Gained or lyg?).tw. | 4,683 |
| 57 | or/41-56 | 6,410,440 |
| 58 | 6 and 57 | 5,802 |
| 59 | *fidaxomicin/ | 453 |
| 60 | (fidaxomicin or clostomicin or dafclir or dificid or dificlir or difimicin or lipiarm#cin or tiacumicin b).tw. | 1,756 |
| 61 | *vancomycin/ | 25,621 |
| 62 | (vancomycin or adimicin or aerovanc or amplobac or balcorin or diatracin or edicin or firvanq or icoplax or ifavac or levovanox or lyphocin or maxivanil or norimko or selamat or vamysin or vanauras or vancam or vancam#cin or vanccostacin or vanco or vancoci* or vancoled or vancom* or vancor or vancosan or vancox or vankom#cin or vanmicina or vanococin or varedet or voncon or vondem).tw. | 80,302 |
| 63 | *metronidazole/ | 17,407 |
| 64 | (metr#nid* or acea or acromona or amevan or amiyodazol or anaerob* or apo metr#nid* or arcazol or arilin? or aristogyl or asiazole or asuzol or atrivyl or biotazol or camezol or clont or cont or danizol or deflamon or dumozol or elyzol or endazole or entizol or farnat or fladex or flagenase or flagesol or flagil or flagizole or flagyl or flasinyl or flazol or flegyl or fossyol or frotin or giardyl or gineflavir or helminzol or ivemetro or klion or keucosan or marphazole or metragyl or metrocream or metrog#l or metrol* or metromidol? or metronide or metronil or metronizadole or metroring or mterozin? or metryl or monasin or nalox or nidazol).tw. | 254,606 |
| 65 | *fecal microbiota transplantation/ | 4,690 |
| 66 | ((f?ecal or gut or stool or intestin*) adj5 (transplant* or enema? or transfus* or infus* or instillat* or bacteriotherap* or fmt)).tw. | 24,852 |
| 67 | or/59-66 | 360,056 |
| 68 | 67 and (17 or 40 or 58) | 10,978 |
| 69 | exp case study/ or exp case report/ or exp letter/ or exp editorial/ or exp preliminary communication/ or exp note/ | 8,592,063 |
| 70 | (exp animal/ or exp invertebrate/ or nonhuman/ or animal experiment/ or animal tissue/ or animal model/ or exp plant/ or exp fungus/) not (exp human/ or human tissue/) | 11,591,644 |
| 71 | 68 not (69 or 70) | 8,573 |
| 72 | limit 71 to yr="2012 -Current" | 6,714 |
| 73 | exp conference paper/ or conference abstract/ or (conference adj (abstract or paper or review or proceeding)).pt. | 5,346,262 |
| 74 | limit 73 to yr="2012 - 2019" | 3,067,187 |
| 75 | 72 not 74 | 5,435 |
| 76 | limit 75 to english language | **3,246** |

Table 2. Summary of included study population characteristics and clinical and economic burden outcomes

| **Study details** | **Study type** | **Country** | **Study setting** | **Study period** | **Population of interest** | **Age (years)** | **Intervention (dose)** | **Key findings** |
| --- | --- | --- | --- | --- | --- | --- | --- | --- |
| Aoki *et al.*, 2019(64) | Phase 3, double-blind, parallel study | Japan | RCT | June 2014–September 2016 | *C. difficile* isolates from adult patients with CDI (N=188) | Range: ≥20 | Fidaxomicin, vancomycin, metronidazole. Dose not specified. | **MIC_50_/MIC_90_ (mg/liter)**   - Fidaxomicin: 0.06/0.12 - Vancomycin: 0.5/0.5 - Metronidazole: 0.1/1 |
| Beran *et al.*, 2017(65) | Retrospective analysis | Czech Republic | Clinical/hospital | 2011–2012 | Patients with microbiologically confirmed CDI (N=64) | NA | Fidaxomicin, vancomycin, metronidazole. Dose not specified. | **MIC_50_/MIC_90_ (mg/liter)**   - Fidaxomicin: 0.06/0.125 - Vancomycin: 0.125/0.25 - Metronidazole: 0.25/0.5 |
| Biggs *et al.*, 2019(39) | Retrospective cohort | UK | Clinical/hospital | April 2017–March 2018 | Patients aged ≥18 years who received fidaxomicin treatment for mild, serve or rCDI (N=38) | Range: ≥18 | Fidaxomicin (200mg twice daily for 10 days) | Across full cohort at 30-days post‑treatment, first any episode/episode:   - Clinical response: 15 (39%)/11(55%) - Clinical failure: 23/9 - In **any CDI episode** at 30 days post‑treatment:  \|  \| Clinical response (N/%) \| Clinical failure (%) \| \| --- \| --- \| --- \| \| Mild (n=16) \| 10 (63) \| 6 \| \| Severe (n=12) \| 5 (42) \| 7 \| \| rCDI (n=10) \| 0 (0) \| 10 \|  - In **first CDI episode** at 30 days post‑treatment:  \|  \| Clinical response (N/%) \| Clinical failure (%) \| \| --- \| --- \| --- \| \| Mild (n=16) \| 9 (64) \| 5 \| \| Severe (n=12) \| 2 (33) \| 4 \| |
| Bouza *et al.*, 2017(48) | Prospective cohort, | Spain | Clinical/hospital | November 2014–November 2015 | Patients presenting with CDI-027 infection (N=141) | Median (IQR): 76 (59–82) | Fidaxomicin, vancomycin and vancomycin taper | \|  \| No recurrence N (%) \| First recurrence N (%) \| \| --- \| --- \| --- \| \| Metronidazole \| 1 (1.1) \| 5 (18.5) \| \| Vancomycin \| 19 (21.6) \| 13 (48.1) \| \| Vancomycin taper \| 53 (60.2) \| 5 (18.5) \| \| Fidaxomicin \| 4 (4.5) \| 1 (3.7) \|  - Patients treated with prolonged vancomycin or standard fidaxomicin experienced fewer recurrences. |
| Cho *et al.*, 2018(40) | Retrospective cohort | US | Clinical/hospital | December 2012–March 2014 | Adult patients admitted to hospital with laboratory confirmed CDI who received ≥3 days of fidaxomicin (N=64) | Mean age: 68.6–82.0 | Fidaxomicin (200mg twice daily) | - Clinical cure at day 14 post-treatment across two healthcare systems: 83–93%. - Rate of recurrence at 30-day/60-day post-treatment: 0–7.0%/0–9.7% - Incidence of hospital readmission: 5–6 (14–21%): CDAD-related: 0–3 (0–50%) |
| Conrad *et al.*, 2022(54) | Retrospective cohort | US | Tertiary care institutions | 2013–2021 | Pediatric patients with recurrent CDI (N=79) | Median (IQR): 13 (7–15.5) | Fidaxomicin (dose not reported) | - Achieved clinical cure: 24 (30.4%). - rCDI among patients who achieved cure: 0% (median follow-up 1.5 years). - rCDI among patients with partial response: 18 (58%) had additional rCDI episode (>60‑day follow-up). - No deaths reported. |
| Cornely *et al.*, 2012(Suppl 1) | Retrospective cohort | US, Canada, European countries | RCT | NA | Patients aged >15 years, with a CDI diagnosis who had received <24 hours of pre-treatment with vancomycin or metronidazole (Per protocol: N=794, mITT: N=962) | Median (IQR): NA | EP-fidaxomicin (200mg twice daily on days 1–5, then once daily  on alternate days 7–25), vancomycin (125mg four times daily for 10 days) | - Clinical response significantly improved (chi-square p<0.05) among EP-fidaxomicin‑treated patients than vancomycin, regardless of ribotype, at day 40, 55 and 90 post-treatment. - Sustained clinical cure at 30-days post‑treatment improved across all subgroups treated with EP-fidaxomicin. - Subgroups investigated: age (≥75 vs <75), CDI severity (severe vs non-severe), previous CDI episode (none/one/two), cancer presence, ribotype (PCR-RT 027 vs other ribotype). |
| Cornely et  al.,  2014(41) | Meta-analysis | US, Canada, European countries | RCT | July 2011–August 2011 | Patients with CDI who received fidaxomicin or standard of care (vancomycin or metronidazole) treatment (N=1,509 (5 studies)) | NA | Fidaxomicin, vancomycin, metronidazole | Direct comparison fidaxomicin versus vancomycin, OR (95% CI):   - Clinical cure: 1.17 (0.82–1.66) - Recurrence: 0.47 (0.34–0.65)* - Sustained cure: 1.75 (1.35–2.27)*   Indirect comparison fidaxomicin versus metronidazole, OR (95% CI):   - Clinical cure: 2.01 (0.99–4.1) - Recurrence: 0.42 (0.18–0.96)* - Sustained cure: 2.55 (1.44–4.51)*   *statistically significant findings |
| Cornely *et al.*, 2019(49) | Phase 3b/4, parallel, superiority, open-label | European countries (21 countries) | RCT | 2014–2016 | Patients aged ≥60 years and were hospitalized with clinically confirmed CDI (N=356) | Median (range): 75 (60–95) | Fidaxomicin (200mg twice daily for 10 days), vancomycin (125mg four times daily for 10 days) | Recurrence fidaxomicin versus vancomycin (p‑value):   - Within 14 days: 7.6% vs 7.4% (0.003) - During 15–28 days: 13.1% vs 11.1% (NR) - No recurrence at 28 days: 80.3% vs 64.5% (NR)   Time to recurrence vancomycin vs fidaxomicin:   - HR 2.17 (95% CI: 1.09–4.34); p=0.03 |
| Dai *et al.*, 2022(58) | SLR and MA | US, Switzerland, Czech Republic | Clinical/hospital | 1954–January 2022 | CDI patients requiring antibiotic therapy (N=2,151 across 10 studies) | Mean age range: 47.5–74.6 | Fidaxomicin (200mg twice daily (dose reported by 2/10 studies)), vancomycin (125mg four times daily (reported by 4/10 studies)) | Fidaxomicin versus vancomycin, OR (95% CI):  (>1 favors fidaxomicin, <1 favors vancomycin)   - Clinical cure: 0.4 (0.09–1.68) - Sustained cure: 2.02 (0.36–11.39) - All-cause mortality: 0.73 (0.5–1.07)   (<1 favors fidaxomicin, >1 favors vancomycin)   - Recurrence: 0.69 (0.4–1.2) |
| Dubberke *et al.*, 2022(21) | Retrospective cohort | US | Clinical/hospital | 2016–2018 | Elderly fee-for-service Medicare beneficiaries receiving fidaxomicin or vancomycin in the (a) initial CDI episode and (b) recurrent CDI episode setting (N=380) | NR | Fidaxomicin (dose not reported), vancomycin (dose not reported) | - Fidaxomicin versus vancomycin  \|  \| Difference (%) \| p-value \| \| --- \| --- \| --- \| \| Sustained cure, initial CDI:  4 weeks  8 weeks \| 13.5  13.2 \| 0.0058  0.0114 \| \| Recurrence:  4 weeks  8 weeks \| -8.4  -7.6 \| 0.101  0.1893 \| \| Sustained cure, rCDI:  4 weeks  8 weeks \| 30.1  27.6 \| 0.0002  0.0012 \| |
| Dubberke  et al.,  2022(Suppl 2) | Retrospective cohort | US | Clinical/hospital | 2016–2018 | Patients with a claim for an initial CDI episode (N=15,135) | Range: ≥65 | Fidaxomicin (dose not reported), vancomycin (dose not reported) | Pre- vs post-IDSA guideline update (fidaxomicin as first-line treatment for initial and rCDI)   \|  \| Pre (%) \| Post (%) \| p-value \| \| --- \| --- \| --- \| --- \| \| Sustained cure, initial CDI:  4 weeks  8 weeks \| 56.9  52.9 \| 54.8  49.8 \| 0.01  0.0002 \| \| Recurrence:  4 weeks  8 weeks \| 31.0  35.9 \| 33.8  39.8 \| 0.001  <0.0001 \| \| Sustained cure,  rCDI:  4 weeks  8 weeks \| 57.4  53.3 \| 52.2  46.7 \| 0.0369  0.0084 \| |
| Eiland *et al.*, 2015(22) | Retrospective cohort | US | Clinical/hospital | August 2011–March 2013 | Adults prescribed fidaxomicin for a diagnosis of CDAD, based upon clinical signs and symptoms of disease and a positive PCR test for *C. difficile* toxin B gene (N=60) | Median (range): 66 (18–92) | Fidaxomicin (200mg twice daily for 10 days) | - Median hospital length of stay: 10 days (range: 3–82) - Clinical success: 58 (96.7%) patients - All-cause mortality (not attributed to CDAD or fidaxomicin therapy): 2 (3.3%) patients - Recurrence within 90 days post-treatment: 6 (10.3%) patients - Hospital readmission within 30 days post‑discharge: 4 (6.9%) patients |
| Enoch *et al.*, 2018(56) | Retrospective cohort | UK | Clinical/hospital | November 2014–October 2017 | Adult patients who were treated with fidaxomicin (N=16) | Median (range): 77 (60–89) | Fidaxomicin (200mg twice daily for 10 days) | - Clinical cure, initial CDI: 8 (50%) patients - Time to recurrence post end of treatment (N=2), range: 5–9 days - Sustained response (N=8), 90-days: 3 patients |
| Escudero-Sanchez *et al.*, 2021(23) | Retrospective cohort | Spain | Clinical/hospital | January 2013–December 2017 | Patients who had received fidaxomicin for at least 2 days for the treatment of CDI (N=244) | Mean (SD): 68.1 (18) | Fidaxomicin | - Median time to recurrence: 32 days (IQR: 22–57) - Recurrence (%):  \|  \| Total \| Initial CDI \| 1^st^ rCDI \| ≥2^nd^ rCDI \| \| --- \| --- \| --- \| --- \| --- \| \| ≤8 weeks \| 14.7 \| 6.5 \| 16.3 \| 26.4 \| \| ≤12 weeks \| 19.8 \| 9.7 \| 20.7 \| 35.8 \|  - All-cause mortality (%):  \|  \| Total \| Initial CDI \| 1^st^ rCDI \| ≥2^nd^ rCDI \| \| --- \| --- \| --- \| --- \| --- \| \| ≤8 weeks \| 7.1 \| 12.9 \| 3.3 \| 3.8 \| \| ≤12 weeks \| 11.8 \| 17.2 \| 9.8 \| 5.7 \| |
| Eyre *et al.*, 2014(50) | Retrospective cohort | US, Canada, European countries | RCT | NA | Adults with >3 unformed bowel movements <24 hours before randomization; *had C. difficile* toxin detected in stool; received ≤4 doses of vancomycin or metronidazole for a total period of ≤24 hours; and had ≤1 CDI episode in the previous 3 months (N=1,164 across 2 studies) | NA | Fidaxomicin (200mg twice daily for 10 days), vancomycin (125mg four times daily for 10 days) | Fidaxomicin vs vancomycin:   - Recurrence HR (95% CI): 0.46 (0.35–0.52); p=<0.001.   **Same-strain** relapse (i.e., follow-up isolate with ≤2 SNVs change from baseline)   - Relapse HR (95% CI): 0.4 (0.25–0.66; p=0.0003.   **Reinfection** (i.e., follow-up isolate with >10 SNVs from the baseline)   - Relapse HR (95% CI): 0.33 (0.11–1.01; p=0.05 |
| Fehér *et al.*, 2017(24) | Retrospective cohort | Spain | Clinical/hospital | July 2013–July 2014 | Adult patients with microbiologically documented, symptomatic CDI, who had received fidaxomicin treatment (N=72) | Mean (SD): 64.4 (18.3) | Fidaxomicin (200mg twice daily for median of 11 days) | Number of cases (%):   - Achieving clinical cure: 65 (90.3) - Median time to symptom resolution: 4 days (IQR: 2–6) - Sustained cure at 30-days: 52 (72.2) - Recurrence within 30-days: 12 (16.7) - All-cause mortality within 30-days: 3 (4.2) - CDI-related mortality: 2 (2.8)   Factors associated with absence of sustained cure (statistically significant values only), univariate OR (95% CI); p-value:   - Cardiopathy: 4.58 (1.50–13.99); p=0.006 - McCabe score fatal: 4.05 (1.18–13.85); p=0.02 - Severe illness: 10.09 (2.1–48.41); p=0.001 - Acute kidney failure: 5.25 (1.65–16.71); p=0.003 - Shock/severe sepsis: 7.00 (1.76–27.87); p=0.006 - Additional CDI treatment: 3.75 (1.1–12.78); p=0.043 |
| Filippidis  et al.,  2022(Suppl 3) | Retrospective cohort | Switzerland | Clinical/hospital | January 2014–December 2018 | Patients with confirmed CDI first episodes and available clinical data (N=676) | Median (IQR): 67 (54–78) | Fidaxomicin, vancomycin, metronidazole (all doses listed as at discretion of physician) | Multivariable logistic regression model,  risk of **recurrence** at week 8 (OR, 95% CI):   - Fidaxomicin: 1 (reference) - Vancomycin: 1.01 (0.20–7.46) - Metronidazole: 1.77 (0.46–11.6) |
| Freeman *et al.*, 2020(66) | Retrospective cohort | Czech Republic, France, UK, Germany, Ireland, Spain | Clinical/hospital | 2011–2016 | CDI strain isolates (N=3,499) | Median: 71 | Fidaxomicin, vancomycin, metronidazole (dose NA) | Antibiotic susceptibility:  Geometric mean MIC (mg/L):   - Fidaxomicin: 0.04 - Vancomycin: 0.46 - Metronidazole: 0.7 |
| Gallagher *et al.*, 2015(51) | Retrospective cohort study, single center | US | Clinical/hospital | January 2012–January 2014 | Adult patients who received oral vancomycin or fidaxomicin for CDI treatment (N=95) | Mean: 72.1–73.2 | Fidaxomicin (200mg twice daily for 10 days), vancomycin (125–250mg four times daily for 10 days) | - Adjusted OR for recurrence post-fidaxomicin treatment at 90 days follow-up: 0.33 (95% CI: 0.12–0.93).   Recurrence, number of patients (%):   \|  \| Total \| ≥2^nd^ rCDI \| \| --- \| --- \| --- \| \| Fidaxomicin \| 22 (47.8) \| 8 (36.4) \| \| Vancomycin \| 38 (77.6) \| 10 (26.3) \| \| *p-value for treatment comparison* \| *0.003* \| *0.413* \|   CDI-related readmission at 90 days post-treatment, number of patients (%):   \|  \| Total \| 1^st^ episode \| \| --- \| --- \| --- \| \| Fidaxomicin \| 10 (20.4) \| 0 (0) \| \| Vancomycin \| 19 (41.3) \| 11 (45.8) \| \| *p-value for treatment comparison* \| *0.027* \| *0.007* \|   Total drug costs (USD, cost year NR)   - Fidaxomicin: $62,112 - Vancomycin: $6,646   Total length of stay per intervention group:   - Fidaxomicin: 87 - Vancomycin: 183   Actual costs for CDI-related readmission:   - Fidaxomicin: $196,200 - Vancomycin: $454,800   Hospital losses per patient:   - Fidaxomicin: $3,286 - Vancomycin: $6,333 |
| Gentry *et al.*, 2019(25) | Retrospective matched cohort | US | Clinical/hospital | June 2011–June 2017 | Patients with severe CDI (baseline white blood cell count ≥1.5 times the premorbid level) who received treatment with either fidaxomicin or oral vancomycin within 72 hours of a positive toxin result (N=852) | Mean (SD): 69.7 (11.1) | Fidaxomicin, vancomycin | Clinical failure, number of patients (%)   - Fidaxomicin: 68 (31.9) - Vancomycin: 163 (25.5) - *P-value for treatment comparison: 0.071*   Recurrence at 90-days post-treatment, number of patients (%):   - Fidaxomicin: 52 (24.4) - Vancomycin: 156 (24.4) - *P-value for treatment comparison: 1.0*   All-cause mortality rate:   \|  \| 30-days \| 90-days \| 180-days \| \| --- \| --- \| --- \| --- \| \| Fidaxomicin \| 23 (10.8) \| 48 (22.5) \| 62 (29.1) \| \| Vancomycin \| 75 (11.7) \| 140 (21.9) \| 186 (29.1) \| \| *p-value for treatment comparison* \| *0.71* \| *0.85* \| *1.0* \| |
| Giancola *et al.*, 2018(52) | Retrospective cohort | US | Clinical/hospital | June 2011–October 2016 | Adult patients who received at least one dose of fidaxomicin (N=880) | Range: >18 | Fidaxomicin (dose NR) | **Secondary analyses** in sub cohort (N=251):  Number of patients (%):   - Clinical failure: 57 (24.4) - Recurrence: 17 (6.8) - All-cause mortality at 30-days: 8 (3.2) Median length of hospital stay: 5.6 days (range: 0.9–84.3) |
| Goldenberg *et al.*, 2015(74) | Retrospective cohort | England | Clinical/hospital | December 2013–October 2014 | Hospitalized patients with a positive toxin A/B test for *C. difficile* within the evaluation period (pre- or post-FDX) were identified from microbiology department records (N=NA; 7 hospitals reporting data for ≥20 patients) | Median : Primary episode: 70–81; Recurrent episode: 43.5–84.5 | Fidaxomicin (dose NR) | - Relative change in recurrence rate from pre- to post- fidaxomicin use showed fewer recurrences in 5/7 hospitals (-8.5–-81.0%), and increased recurrence in 2/7 hospitals (7.4–7.8%). - Readmission rate with 30-days of recurrence decreased across 5 hospitals and was equivalent in 1 (data not provided for 1 hospital). |
| Gomez Delgado *et al.*, 2022(55) | Retrospective cohort | Spain | Clinical/hospital | January 2014–April 2021 | All patients treated by fidaxomicin from pharmacy dispensation (N=41) | Mean (range): 69 (21–99) | Fidaxomicin (200mg four times daily for at least 10 days) | Number of patients (%) at week 8 post-treatment:   - Without recurrence: 34 (82.9) - Mortality rate: 9 (22) |
| Green *et al.*, 2021(42) | Retrospective cohort | US | Clinical/hospital | March 2014–October 2020 | Adult HSCT recipients diagnosed with an initial episode of CDI within 100 days following HSCT, treated with oral vancomycin or fidaxomicin (N=61) | NA | Fidaxomicin, vancomycin (both median course for 14 days, dose NR) | % patients at 30-days post-treatment, fidaxomicin vs vancomycin:   - Recurrence: 0% vs 10.3%; p=0.287 - Sustained clinical cure: 100% vs 84.6%; p=0.079 - Initial clinical cure: 100% vs 92.3%; p=0.547 |
| Guery *et al.*, 2017(26) | Phase 3b RCT | European countries (21 countries) | RCT | 2014–2016 | Hospitalized patients aged ≥60 years with clinically confirmed CDI (N=356) | Median (IQR): 75 (EP-fidaxomicin: 69–83; vancomycin: 67–82) | Fidaxomicin (400mg per day for 10 days) | Number of patients (%):   - Clinical cure (N=286)  \| All patients \| Age (years) \| \| Concomitant antibiotic use \| \| \| --- \| --- \| --- \| --- \| --- \| \| ≥65 \| <65 \| Yes \| No \| \| 92.2 \| 31.3 \| 93.8 \| 92.1 \| 92.3 \|  - Sustained clinical cure:  \| All patients \| Age (years) \| \| Concomitant antibiotic use \| \| \| --- \| --- \| --- \| --- \| --- \| \| ≥65 \| <65 \| Yes \| No \| \| 76.5 \| 74.2 \| 80.4 \| 78.9 \| 74.5 \|   Recurrence, number of patients (%), within:   - 1 month: 21 (9.0) - 2 months: 32 (13.7) - 3 months 38 (16.3) - >3 months: 2 (0.9) |
| Guery *et al.*, 2021(46) | Retrospective cohort | France | Clinical/hospital | September 2014–November 2017 | Patients hospitalized and diagnosed with CDI (N=294) | Mean (SD): 68.4 (17.3) | EP-fidaxomicin (200mg twice daily on days 1–5, then once daily on alternate days 7–25), vancomycin (125mg four times daily on days 1–10) | Modified full analysis set:   - Recurrence, number of patients (%)  \|  \| Day 40 \| Day 55 \| Day 90 \| \| --- \| --- \| --- \| --- \| \| EP-fidaxomicin \| 3 (2) \| 7 (4) \| 11 (6) \| \| Vancomycin \| 30 (17) \| 32 (18) \| 34 (19) \| \| *OR (95% CI)* \| *0.09 (0.03–0.29)* \| *0.2 (0.08–0.46)* \| *0.29 (0.14–0.6)* \| \| *p-value for OR* \| *<0.0001* \| *<0.0001* \| *0.00073* \|  - Sustained clinical cure, 30-days post-treatment  \|  \| Day 40 \| Day 55 \| Day 90 \| \| --- \| --- \| --- \| --- \| \| EP-fidaxomicin \| 133 (75) \| 124 (70) \| 116 (66) \| \| Vancomycin \| 106 (59) \| 99 (55) \| 92 (51) \| \| *OR (95% CI)* \| *2.1 (1.32–3.34)* \| *1.91 (1.23–2.98)* \| *1.8 (1.17–2.77)* \| \| *p-value for OR* \| *0.0013* \| *0.0038* \| *0.0070* \| |
| Hall et al.,  2022(Suppl 4) | Retrospective cohort | US | Clinical/hospital | July 2011–June 2018 | Patients with index case of CDI in-hospital (N=15,674) | Mean: 66.7 | Fidaxomicin, vancomycin (dose NR) | <1 favors fidaxomicin, >1 favors vancomycin:   - Risk of recurrence within 90-days of treatment, HR (95% CI): 0.67 (0.5–0.9). |
| Hvas *et al.*, 2019(27) | RCT, active comparator, open-label | Denmark | RCT | April 2016–June 2018 | Adult patients with rCDI and documented recurrence within 8 weeks after stopping anti-CDI treatment (N=64) | Median (range): 68 (22–90) | Fidaxomicin (200mg twice daily for 10 days), vancomycin (125mg four times daily for 10 days),  FMT (dose NA) | Clinical resolution, number of patients (%):   \|  \| Week 1 \| Week 8 \| \| --- \| --- \| --- \| \| Fidaxomicin \| 14 (58) \| 10 (42) \| \| Vancomycin \| 6 (38) \| 3 (19) \| \| FMT \| 21 (88) \| 22 (92) \|   P-values for treatment comparison:  **Week 1**   - Fidaxomicin vs vancomycin: 0.27 - Fidaxomicin vs FMT: 0.02 - FMT vs vancomycin: 0.002   **Week 8**   - Fidaxomicin vs vancomycin: 0.13 - Fidaxomicin vs FMT: 0.0002 - FMT vs vancomycin: <0.0001 |
| Imwattana *et al.*, 2021(Suppl 5) | Retrospective cohort | Thailand | Clinical/hospital | 2017–2018 | Diarrheal stool samples collected from patients with a high suspicion of index CDI (N=321) | NA | Fidaxomicin, vancomycin, metronidazole (dose NA) | Antibiotic susceptibility  **MIC_50_/MIC_90,_ range (mg/L)**   - Fidaxomicin: 0.125/0.25, 0.03–0.25 - Vancomycin: 1/2, 0.5–2 - Metronidazole: 0.25/0.25, 0.06–1 |
| Jon J *et al.*, 2021(67) | Retrospective cohort | UK | Laboratory | 2012-2016 | Isolates from patients with confirmed CDI infection (N=75) | NA | Fidaxomicin, vancomycin, metronidazole (dose NA) | Antibiotic susceptibility  **Geometric mean MIC (mg/L)**   \|  \| Time period \| \| \| --- \| --- \| --- \| \| 1980–1986 \| 2012–2016 \| \| Fidaxomicin \| 0.028 \| 0.05 \| \| Vancomycin \| 0.704 \| 0.839 \| \| Metronidazole \| 0.171 \| 0.283 \| |
| Khun *et al.*, 2022(68) | Prevalence, epidemiological testing | Vietnam | Clinical/hospital | October 2020–February 2021 | Diarrheal stool samples from pediatric patients aged 0–17 years (N=151 samples) | Median: 10 months | Fidaxomicin, vancomycin, metronidazole (dose NA) | Antibiotic susceptibility  **Geometric mean MIC (mg/L)**   - Fidaxomicin: 0.124 - Vancomycin: 1.023 - Metronidazole: 0.232 |
| Knight *et al.*, 2015(69) | Epidemiological | Australia | Laboratory | August 2013–September 2014 | Isolates positive for CDI (regardless of patient symptoms) (N=NA) | Median: 69–71 | Fidaxomicin, vancomycin, metronidazole (dose NA) | Antibiotic susceptibility  **MIC_50_/MIC_90,_ range (mg/:)**   - Fidaxomicin: 0.03/0.12, <0.008–0.5 - Vancomycin: 1/2, 0.5–2 - Metronidazole: 0.25/0.5, 0.12–1 |
| Lee *et al.*, 2016(43) | Retrospective cohort | Canada | RCT | 2006–2009 | Patients aged ≥16 years, had CDI (defined as >3 unformed bowel movements) in the 24 hours before randomization, and had *C. difficile* toxin A or B (or both) in stool within 48 hours before randomization (N=406) | Range: ≥16 | Fidaxomicin (200mg twice daily), vancomycin (125mg four times daily) | Fidaxomicin versus vancomycin   - Clinical response  \| **Subgroup** \| **Difference (CI)** \| \| --- \| --- \| \| Age ≥65 years \| -0.9 (-10.5–8.7) \| \| Concomitant antibiotic use \| 11.1 (-9.3–30.0) \| \| Cancer \| -4.5 (-24.4–14.7) \| \| Renal dysfunction \| -10.5 (-24.2–14.7) \| \| BI strain \| -4.7 (-16.6–7.4) \| \| Non-BI strain \| -2.4 (8.7–3.8) \|  - Sustained clinical response  \| **Subgroup** \| **% change (p-value)** \| \| --- \| --- \| \| Age ≥65 years \| 20.1 (0.076) \| \| Concomitant antibiotic use \| 40.4 (0.056) \| \| Cancer \| 27.7 (0.184) \| \| Renal dysfunction \| 8.0 (0.641) \| \| BI strain \| 11.2 (0.393) \| \| Non-BI strain \| 19.9 (0.021) \|  - Recurrence  \| **Subgroup** \| **% change (p-value)** \| \| --- \| --- \| \| Age ≥65 years \| -48.0 (0.026) \| \| Concomitant antibiotic use \| -58.1 (0.036) \| \| Cancer \| -82.2 (0.0616) \| \| Renal dysfunction \| -38.0 (0.143) \| \| BI strain \| -43.2 (0.113) \| \| Non-BI strain \| -58.2 (0.004) \| |
| Liao *et al.*, 2021(44) | Retrospective cohort | NA | RCT | –February 2021 | Adult patients with CDI who received guidelines-recommended regimens of fidaxomicin or vancomycin (N=1,359 across 3 studies) | Mean (SD): 64.4 (17.44) | Fidaxomicin, vancomycin | Fidaxomicin vs vancomycin   - Initial clinical cure: 87.2% vs 86.5% - Recurrence: 15% vs 25.9% - Mortality: 5.8% vs 5.9% - Relative risk of recurrence: 0.58 (95% CI: 0.45–0.75; p<0.0004) |
| Liao *et al.*, 2022(59) | SLR and MA | Multinational (including Canada, US, Japan, European countries) | Clinical/hospital | From inception of searched databases to September 2021 | Adults with CDI treated with vancomycin or fidaxomicin, with reported recurrence rates (N=3,944 across 14 studies) | Mean age range: 46–75 | Fidaxomicin (200mg [9/14 studies reported] twice daily, 7–10 days, tapered, or median 11–14 days), vancomycin (125–500mg (11/14 studies reported] four times daily, tapered, median 11–14 days) | (<1 favors fidaxomicin, >1 favors vancomycin)  Fidaxomicin vs vancomycin, relative risk of recurrence (95% CI):   - Overall: 0.69 (0.52–0.94) - Initial vs any episode of rCDI: 0.6 (0.45–0.79) - 1^st^ rCDI vs 2^nd^ or higher rCDI: 0.57 (0.38–0.84) - Based on CDI severity: 0.51 (0.36–0.72) |
| McDaniel *et al.*, 2022(72) | Retrospective, quasi-experimental cohort | US | Clinical/hospital | August 2016–November 2017; May 2018–January 2020 | Adult patients with a positive CDI test (Pre-implementation cohort: N=186, post-implementation: N=187) | Median: 67–68 | Treatment uptake pre/post-implementation of IDSA/SHEA guideline 2021 update:  **Pre:** metronidazole (47.8%), vancomycin (50.5%), fidaxomicin (1.6%).  **Post:** metronidazole (1.6%), vancomycin (41.7%), fidaxomicin (56.7%) | Pre-implementation/post-implementation (%, p-value for difference):   - Clinical cure: 84.4/94.1 p=0.002 - Recurrence: - 30-days: 18/6.4, p=0.001 - 90-days: 27.1/14.9, p=0.009 - Sustained response at 90-days: 55.9/73.3, p=<0.001 - CDI-related readmission: - 30-days: 12.7/4.6, p=0.007 - 90-days: 18.9/8.5, p=0.006 - All-cause readmission: - 30-days: 35.7/27.7, p=0.109 - 90-days: 45.4/38.2, p=0.181   Pre vs post-implementation   - Index length of hospital stay: median 8 days (IQR: 5–14) vs 6 days (4–13), p=0.093   Pre vs post-implementation (difference, p-value)   - Total treatment cost: -$2,588.63, p=0.048 - Total direct cost: -$1,285.73, p=0.07 |
| Mekideche *et al.*, 2018(28) | Retrospective cohort | France | Clinical/hospital | June 2014–June 2015 | Adult patients with CDI who were treated with fidaxomicin (N=50) | Mean: 69.6 | Fidaxomicin (dose NR) | Recurrence rate of 32%. |
| Mikamo *et al.*, 2018(29) | Phase 3, double-blind, parallel | Japan | RCT recruited from hospitals | June 2014–September 2016 | Patients hospitalized with symptomatic CDI aged ≥20 years (N=215) | Mean (SD): 74–75 | Fidaxomicin (200mg twice daily for 10 days), vancomycin (500mg four times daily for 10 days), metronidazole (dose NR) | **Full analysis set**  Global cure rate, n (%), 95% CI:   - Fidaxomicin: 70 (67.3), 58.3–76.3 - Vancomycin: 71 (65.7), 56.8–74.7 |
| Novotný *et al.*, 2018(57) | Retrospective cohort, single center | Slovakia | Clinical/hospital | January 2013–January 2018 | Adult patients with CDI who were treated with fidaxomicin (N=NA) | Mean (range): 72.45 (35–94) | Fidaxomicin (200mg twice daily) | Number of patients (%):   - Clinical success: NR (86.7) - Clinical failure: 8 (13.3%) - Recurrence: - First, within 8-weeks: 2 (10.5) - Second or higher, timepoint NR: 2 (10.5) - Mortality - CDI-related: 2 (3.3) - All-cause: 6 (10) |
| O'Gorman *et al.*, 2018(47) | RCT, phase 2a, multicenter, single arm, open-label | US, Canada | RCT | October 2012–March 2014 | Pediatric patients with confirmed CDAD (N=38) | mean (range, SD) months: 99.4 (11–206, 68.9) | Fidaxomicin (200mg twice daily for 10 days) | - At end of therapy, mITT population  \| Age subgroup \| Clinical response \| Sustained clinical response \| Recurrence \| \| --- \| --- \| --- \| --- \| \| All patients \| 35 (92.1) \| 11 (31.4) \| 25 (65.8) \| \| 6–23 months \| 8 (88.9) \| 3 (37.5) \| 6 (66.7) \| \| 2–<6 years \| 6 (75) \| 2 (33.3) \| 4 (50) \| \| 6–<12 years \| 9 (100 \| 2 (22.2) \| 7 (77.8) \| \| 12–<18 years \| 12 (100) \| 4 (33.3) \| 8 (66.7) \| |
| Okumura *et al.*, 2020(61) | SLR and MA | Austria, Canada, European countries, Japan, US | RCT | July 2011–September 2016 | Adult patients who received fidaxomicin, vancomycin or metronidazole with confirmed CDI of any severity (N=NR, included 7 studies) | Range: >18 | Fidaxomicin (200mg twice daily for 10 days), vancomycin (125mg/500mg four times daily for 10 days/three times daily for 10 days), metronidazole (500mg three times daily for 10 days) | Fixed effects model fidaxomicin vs comparator, median OR (95% CI):  **Clinical cure**   - Vancomycin: 1.07 (0.78–1.48) - Metronidazole: 1.77 (1.11–2.83)   **Recurrence**   - Vancomycin: 0.5 (0.37–0.68) - Metronidazole: 0.44 (0.27–0.72) |
| Olivares *et al.*, 2022(73) | Retrospective cohort | NA | Clinical/hospital | October 2020–September 2021 | Patients with pseudomembranous colitis treated for CDI (N=97) | Median (SD): 72 (16) | Fidaxomicin, vancomycin (dose NR) | Total treatment costs, fidaxomicin/vancomycin to achieve cure (EUR):   - One line: NR /€3,216 - Two or three lines: €30,300/€2,266   Among patients aged ≥65 years:   - Achievement of cure: €102,453/ €6,461 |
| Patel *et al.*, 2021(30) | Retrospective cohort | US | Clinical/hospital | January 2008–July 2017 | Patients hospitalized with CDI and treated with vancomycin (N=54) | Mean (SD): 74.9 (11.1) | Fidaxomicin, vancomycin (dose NR) | Recurrence within 60-days post-treatment (%):   - Fidaxomicin: 22.2% - Vancomycin: 7.9% - P-value for treatment comparison: 0.06   All-cause mortality, within 30-days/60-days (%):   - Fidaxomicin: 31.1/21.1 - Vancomycin: 31.5/21.1 - P-value for treatment comparison: 0.27/0.07 |
| Penziner *et al.*, 2015(75) | Retrospective cohort | US | Clinical/hospital | August 2011–April 2014 | Adult patients who received fidaxomicin therapy for at least two consecutive days (N=50) | Mean (SD): 61 (19) | Fidaxomicin (dose NR) | - Treatment response  \| **Subgroup** \| **%** \| \| --- \| --- \| \| Overall \| 64 \| \| Disease severity: Severe and severe complicated CDI \| 46 \| \| Disease severity: Mild-to-moderate CDI \| 81 \| \| Strain: NAP1 strain \| 52 \| \| Strain: non-NAP1 strain \| 71 \| \| CCU admission: CCU \| 60 \| \| CCU admission: Non-CCU \| 67 \|   Length of hospital stay, median days (IQR)   \| **Subgroup** \| **Median** \| **IQR** \| \| --- \| --- \| --- \| \| All patients \| 19 \| 8–29 \| \| CCU admission \| 17.5 \| 7–27 \| \| No CCU admission \| 18.5 \| 9–31 \| |
| Polivkova *et al.*, 2021(31) | Cohort | Czech Republic | Clinical/hospital | October 2013–March 2016 | Patients with confirmed CDI (N=271) | Mean, median: 75.2, 78 | Fidaxomicin (200mg twice daily for 10 days), vancomycin (125mg four times daily for 10 days), metronidazole (500mg three times daily for 10 days) | Univariate logistic regression analysis, fidaxomicin vs comparator, OR (95% CI); p-value: **Sustained clinical response at 60-days post-treatment**   \| **Subgroup** \| **vs MTZ** \| **vs VAN** \| \| --- \| --- \| --- \| \| Overall \| 3.8 (1.8–8.4); p=0.0007 \| 4.8 (2.2–10.5); p=0.0001 \| \| Initial CDI episode \| 3.5 (1.1–11.7); p=0.0380 \| 4.7 (1.4–16.0);  p=0.0136 \| \| First rCDI \| 14.2 (1.8–109.9); p=0.0112 \| 13.2 (2.8–62.7); p=0.0011 \| \| Multiple rCDI \| - \| 0.5 (0.1–5.6); p=0.5905 \| \| Non-severe CDI \| 3.9 (1.6–9.7); p=0.0027 \| 5.7 (2.3–14.0); p=0.0002 \| \| Severe CDI \| 12.5 (1.1–143.4); p=0.0425 \| 2.8 (0.5–14.9); p=0.2399 \|   **Recurrence at 60-days post-treatment**   \| **Subgroup** \| **vs MTZ** \| **vs VAN** \| \| --- \| --- \| --- \| \| Overall \| 0.2 (0.1–0.6); p=0.0013 \| 0.2 (0.1–0.5); p=0.0003 \| \| Initial CDI episode \| 0.1 (0.0–0.8); p=0.0296 \| 0.1 (0.0–0.7); p=0.0249 \| \| First rCDI \| 0 (0–0.6); p=0.0185 \| 0 (0–0.6); p=0.0027 \| \| Multiple rCDI \| - \| 1.9 (0.2–20.8); p=0.5905 \| \| Non-severe CDI \| 0.2 (0.0–0.6); p=0.0036 \| 0.2 (0.1–0.5); p=0.0006 \| \| Severe CDI \| 0.1 (0.0–0.5); p=0.0889 \| 0.3 (0.0–3.1); p=0.2805 \|   **Mortality at 60-days post-treatment (N)**   \| **Subgroup** \| **FDX** \| **MTZ** \| **VAN** \| \| --- \| --- \| --- \| --- \| \| Overall \| 5 \| 12 \| 15 \| \| Initial CDI episode \| 1 \| 25 \| 18 \| \| First rCDI \| 1 \| 3 \| 11 \| \| Multiple rCDI \| 5 \| - \| 1 \| \| Non-severe CDI \| 2 \| 5 \| 8 \| \| Severe CDI \| 3 \| 7 \| 7 \| |
| Putsathit *et al.*, 2021(70) | Retrospective cohort | Australia | Clinical/hospital | 2015–2018 | CDI strain isolates (N=1,091) | NA | Fidaxomicin, vancomycin, metronidazole (dose NA) | Antibiotic susceptibility   \|  \| MIC­_50_ \| MIC_90_ \| \| --- \| --- \| --- \| \| Fidaxomicin \| 0.125 \| 0.125 \| \| Vancomycin \| 2 \| 2 \| \| Metronidazole \| 0.25 \| 0.25 \|   **Geometric mean MIC (mg/L):**   - Fidaxomicin: 0.101 - Vancomycin: 1.7 - Metronidazole: 0.229 |
| Rinaldi *et al.*, 2021(53) | Retrospective cohort | US | Clinical/hospital | January 2013–May 2019 | Patients with confirmed recurrent CDI (N=135) | Median (range): 61 (18–89) | Fidaxomicin (dose NR, median course for 10 days), vancomycin (125mg, 250mg, or 500mg twice daily for median 14 days) | At 6-months follow-up, number of patients (%):  **Treatment failure**   - Fidaxomicin: 6 (17) - Vancomycin: 8 (8) - *P-value for treatment comparison: 0.13*   **Recurrence (index)**   - Fidaxomicin: 7 (20) - Vancomycin: 11 (11) - *P-value for treatment comparison: 0.18*   **Recurrence (reinfection post-index)**   - Fidaxomicin: 5 (14) - Vancomycin: 12 (12) - *P-value for treatment comparison: 0.73*   All-cause in-hospital mortality (timepoint NR):   - Fidaxomicin: 2 (6) - Vancomycin: 4 (4) |
| Rokkas *et al.*, 2019(62) | MA, Bayesian Network | Canada, Denmark, Italy, US, Netherlands | RCT | –December 2018 | Participants with CDI who experienced resolution of CDI-related symptoms (N=348 across 6 studies) | NA | Fidaxomicin, vancomycin, DFMT, placebo, AFMT | **Clinical resolution of symptoms, pairwise comparisons**   \| **Treatment comparison** \| \| **OR (95% CI)** \| \| --- \| --- \| --- \| \| Vancomycin \| Fidaxomicin \| 1.06 (0.32-3.49) \| \| DFMT \| Placebo \| 2.29 (1.00-5.12) \| \| Placebo \| AFMT \| 2.92 (0.39-28.61) \| \| AFMT \| Vancomycin \| 3 (0.20-22.06) \| \| AFMT \| Fidaxomicin \| 3.26 (0.16-30.93) \| \| Placebo \| Vancomycin \| 9.22 (2.21-39.86) \| \| Placebo \| Fidaxomicin \| 9.57 (1.54-70.82) \| \| DFMT \| Vancomycin \| 20.02 (7.05-70.03) \| \| DFMT \| Fidaxomicin \| 22.01 (4.38-109.63) \| |
| Spiceland *et al.*, 2018(32) | Retrospective cohort, multicenter | US | Clinical/hospital | August 2011–July 2015 | Patients with CDI diagnosis and treatment with fidaxomicin (N=81) | Median (range): 55.9 (19.2–85.5) | Fidaxomicin (200mg twice daily for 10 days) | Overall, number of patients (%):   - Complete treatment response: 73 (90) - Recurrence within 8 weeks: 14 (19)   Recurrence   \| **Subgroup** \| **N (%)** \| \| --- \| --- \| \| Comorbidity: IBD \| 4 (19) \| \| Prior treatment: FMT \| 1 (25) \| \| Concomitant antibiotic use <8 weeks prior \| 0 (0) \| \| No concomitant antibiotic use <8 weeks prior \| 13 (22) \|   Complete treatment response, OR (95% CI):   - Age group: <65 vs ≥65 years: 0.72 (0.14–3.86) - Immunosuppressed vs not: 0.77 (0.17–3.46) - Median time to recurrence: 20 days (range: 1–46) |
| Sridharan *et al.*, 2018(63) | MA, Bayesian Network | Austria, US, Canada, Italy, Sweden, UK | RCT | –April 2018 | Patients participating in 11 RCTs comparing antimicrobials for Clostridium difficile infection as determined by clinical features and stool toxin assay (N=2,888 across 17 studies) | NA | Fidaxomicin (200 mg twice a day for 10 days), vancomycin (125mg four times daily or 500mg thrice daily for 10 days), metronidazole (500mg thrice daily with rifampicin, 250mg or 375mg four times daily or 400mg thrice daily) | Treatment comparisons for symptomatic cure, pooled OR (95% CI):   \| **Intervention** \| **Comparator** \| **OR** \| **95% CI** \| \| --- \| --- \| --- \| --- \| \| Mixed treatment \| \| \| \| \| Vancomycin \| Fidaxomicin \| 1.6 \| 1.2–2 \| \| Metronidazole \| Fidaxomicin \| 2.2 \| 1.3–3.7 \| \| Vancomycin \| Metronidazole \| 0.7 \| 0.5–1.1 \| \| Direct treatment \| \| \| \| \| Vancomycin \| Fidaxomicin \| 1.6 \| 1.2–2 \| \| Vancomycin \| Metronidazole \| 0.7 \| 0.4–1.1 \| |
| Tariq *et al.*, 2017(Suppl 6) | Prospective, retrospective cohort | US | Clinical/hospital | January 2013–May 2015 | Patients with ≥3 CDI episodes established by a positive C difficile stool assay in the presence of diarrhea, and previous treatment with first-line therapies for CDI (metronidazole, vancomycin, or fidaxomicin), or a 6- to 8-week tapering course of vancomycin (N=211) | Median (range): 65 (18–93) | Fidaxomicin (dose NR, course for 10 days), vancomycin (dose NR course for 14 days), FMT (dose NA) | Treatment failure, number of patients (%):   - Fidaxomicin: 1 (25) - Vancomycin: 11 (26) - FMT: 7 (97) |
| Tashiro *et al.*, 2022(60) | SLR and MA | America, Canada, European countries, Japan | RCT | –October 2021 | Patients with confirmed CDI (N=NR, six studies in total) | Range: ≥16 | Fidaxomicin (200mg twice daily for 10 days), vancomycin (125mg four times daily for 10 days) | Fidaxomicin vs vancomycin, risk ratio (95 % CI)  **Global cure rate**   \| **Subgroup** \| **RR** \| **95% CI** \| \| --- \| --- \| --- \| \| Non severe CDI \| 1.18 \| 1.09–1.26 \| \| Severe CDI cases \| 1.23 \| 0.93–1.64 \| \| Initial CDI cases \| 1.19 \| 1.09–1.29 \| \| Recurrent CDI cases \| 1.21 \| 0.98–1.49 \| \| Non-BI/AP1/027 infection \| 1.29 \| 1.16–1.43 \| \| BI/AP1/027 infection \| 1.05 \| 0.76–1.45 \| \| Non-concomitant antibiotic use \| 1.22 \| 1.07–1.40 \| \| Concomitant antibiotic use \| 1.18 \| 0.94–1.48 \| \| Age <65 years \| 1.17 \| 1.06–1.3 \|   **Recurrence**   \| **Subgroup** \| **RR** \| **95% CI** \| \| --- \| --- \| --- \| \| Non severe CDI \| 0.61 \| 0.45–0.84 \| \| Severe CDI cases \| 0.41 \| 0.23–0.72 \| \| Initial CDI cases \| 0.54 \| 0.40–0.73 \| \| Recurrent CDI cases \| 0.62 \| 0.36–1.07 \| \| Non-BI/AP1/027 infection \| 0.35 \| 0.23–0.55 \| \| BI/AP1/027 infection \| 0.87 \| 0.4–1.9 \| \| Non-concomitant antibiotic use \| 0.47 \| 0.28–0.8 \| \| Concomitant antibiotic use \| 0.69 \| 0.44–1.08 \| \| Age <65 years \| 0.55 \| 0.37–0.82 \| |
| Thorpe *et al.*, 2019(71) | Prevalence, epidemiological testing | US | Clinical/hospital | 2013–2016 | *C. difficile* isolates from toxin-positive stool samples (N=1,889) | NA | Fidaxomicin, vancomycin, metronidazole (dose NA) | **Antibiotic susceptibility**  Fidaxomicin:   \| REA group \| MIC_50_ \| MIC_90_ \| MIC range \| \| --- \| --- \| --- \| --- \| \| BI \| 0.25 \| 0.5 \| ≤0.004–1 \| \| Non-BI \| 0.25 \| 0.5 \| ≤0.004–1 \|   Vancomycin:   \| REA group \| MIC_50_ \| MIC_90_ \| MIC range \| \| --- \| --- \| --- \| --- \| \| BI \| 2 \| 4 \| ≤0.25–8 \| \| Non-BI \| 1 \| 2 \| ≤0.25–4 \|   Metronidazole   \| REA group \| MIC_50_ \| MIC_90_ \| MIC range \| \| --- \| --- \| --- \| --- \| \| BI \| 2 \| 2 \| 0.12–4 \| \| Non-BI \| 0.5 \| 1 \| ≤0.06–4 \| |
| Tieu *et al.*, 2018 (33) | Retrospective, observational cohort study | US | Clinical/hospital | June 2011–December 2015 | Propensity score-matched patients aged ≥18 years who had first or second recurrent CDI episode (N=260) | Mean (SD): 67.4 (15.5) | Fidaxomicin (dose NR, course for 10 days), vancomycin (dose NR, course for 10 days) | Number of patients (%):  **Treatment failure**   - Fidaxomicin: 4 (6.15) - Vancomycin: 2 (1.03) - *P-value for treatment comparison: 0.036*   **Recurrence (index)**   - Fidaxomicin: 15 (23.1) - Vancomycin: 41 (21.0) - *P-value for treatment comparison: 0.73*   **All-cause mortality at 30-days follow-up**   - Fidaxomicin: 0 (0) - Vancomycin: 8 (4.1) - *P-value for treatment comparison: 0.21* |
| Wilcox *et al.*, 2018(Suppl 7) | Retrospective cohort | NA | RCT | NA | Patients aged >60 years with positive local test for CDI (N=286) | Range: >60 | EP-Fidaxomicin (200mg twice daily on days 1–5 and once daily on alternate days on Days 7–25), vancomycin (125mg four times daily for 10 days) | Rate of sustained clinical cure at 30-days post EOT, difference in Chi-square test: 12.9%, p=0.042. |
| Wilcox *et al.*, 2018 (34) | Retrospective cohort | NA | RCT | NA | Patients aged ≥60 years with CDI (N=356) | Range: ≥60 | EP-Fidaxomicin (200mg twice daily on days 1–5 and once daily on alternate days on Days 7–25), vancomycin (125mg four times daily for 10 days) | Recurrence rate at 90-days post-treatment:   - EP-fidaxomicin: 11(6) - Vancomycin: 34 (19) |
| Wolf *et al.*, 2019(Suppl 8) | Phase 3 multicenter, investigator blind, parallel trial | US, Canada, Europe | RCT | NA | Pediatric patients with diagnosed CDI (N=142) | Median: 5 | Fidaxomicin (16 mg/kg twice daily, max. 400mg per day for 0 to <6 years, or 200mg tablets twice daily for patients aged 6 to <18 years for 10 days), vancomycin (10 mg/kg oral liquid 4 times daily maximum 500mg per day for patients aged 0 to <6 years, or 125mg capsules 4 times daily for those aged 6 to <18 years for 10 days) | **Fidaxomicin vs vancomycin**   - Confirmed clinical response 30-days post-EOT  \| **Subgroup** \| **Risk diff.** \| **95% CI** \| \| --- \| --- \| --- \| \| All evaluable patients \| 18.8 \| 1.5–35.3 \| \| Age group: <2 years \| -15 \| -50.8–20.8 \| \| Age group: ≥2 years \| 27.7 \| 8.2–47.1 \| \| Immunocompromised \| 11.3 \| -22.8–42.2 \| \| Non-immunocompromised \| 25.8 \| 3.2–46.8 \|  - Recurrence at 30-days post-EOT  \| **Subgroup** \| **Risk diff.** \| **95% CI** \| \| --- \| --- \| --- \| \| All evaluable patients \| -15.8 \| -34.5–0.5 \| \| Age group: <2 years \| -6.8 \| -40.3–26.7 \| \| Age group: ≥2 years \| -20.3 \| -41.4–0.7 \| \| Immunocompromised \| -17.6 \| -44–8.8 \| \| Non-immunocompromised \| -13.4 \| -38.7–7.8 \| |
| Yen *et al.*, 2022(45) | Retrospective cohort | US, Canada | Clinical/hospital | NA | Patients with recurrent CDI who had not received any form of FMT as treatment (N=412) | NA | Fidaxomicin (dose NR), vancomycin (dose NR) received pre-FMT procedure | Clinical cure at 30-days post-FMT, OR (95% CI):   - Fidaxomicin: 2.16 (1.06–4.40) - Vancomycin: 1.92 (0.59–8.77) |

AFMT: autologous fecal microbiota transplantation; CCU: critical care unit; CDAD: clostridium difficile-associated diarrhea; CDI: clostridioides difficile infection; CI: confidence interval; DFMT: donor fecal microbiota transplantation; EOT: end of treatment; EP: extended-pulse; EUR: Euro; FDX: fidaxomicin; FMT: fecal microbiota transplantation; HR: hazard ratio; L: liter; HSCT: hematopoietic stem cell transplantation; IBD: inflammatory bowel disease; IQR: interquartile range; ITT: intention to treat; kg: kilogram; MA: meta-analysis; mg: milligram; MIC: minimum inhibitory concentration; MTZ: metronidazole; NA: not applicable; NR: not reported; OR: odds ratio; rCDI; recurrent CDI; RCT: randomized controlled trial; RR: risk ratio; PCR: polymerase chain reaction; SD: standard deviation; SLR: systematic literature review; SNV: single nucleotide variants; UK: United Kingdom; US: United States; USD: US Dollar; VAN: vancomycin.

Suppl 1. Cornely OA, Miller MA, Louie TJ, et al. Treatment of first recurrence of Clostridium difficile infection: fidaxomicin versus vancomycin. Clin Infect Dis. 2012;55 Suppl 2(Suppl 2):S154-61.

Suppl 2. Dubberke ERP, Justin T. Obi, Engels N. Kamal-Bahl, Sachin Desai, Kaushal Stuart, Bruce Doshi, Jalpa A. Impact of Updated Clinical Practice Guidelines on Outpatient Treatment for Clostridioides difficile Infection and Associated Clinical Outcomes. Open forum infectious diseases. 2022;9(10):ofac435.

Suppl 3 Filippidis PK, E. Woelfle, M. Badinski, T. Croxatto, A. Galperine, T. Papadimitriou-Olivgeris, M. Grandbastien, B. Achermann, Y. Guery, B. Treatment and Outcomes of Clostridioides difficile Infection in Switzerland: A Two-Center Retrospective Cohort Study. Journal of Clinical Medicine. 2022;11(13):3805.

Suppl 4 Hall RGC, T. J. Shaw, C. Alvarez, C. A. The Risk of Clostridioides difficile Recurrence after Initial Treatment with Vancomycin or Fidaxomicin Utilizing Cerner Health Facts. Antibiotics. 2022;11(3):295.

Suppl 5. Imwattana KP, P. Knight, D. R. Kiratisin, P. Riley, T. V. Molecular Characterization of, and Antimicrobial Resistance in, Clostridioides difficile from Thailand, 2017-2018. Microbial Drug Resistance. 2021;27(11):1505-12.

Suppl 6. Tariq RW, R. M. Kammer, P. P. Pardi, D. S. Khanna, S. Experience and Outcomes at a Specialized Clostridium difficile Clinical Practice. Mayo Clinic Proceedings: Innovations, Quality and Outcomes. 2017;1(1):49-56.

Suppl 7. Wilcox MC, O. A. Guery, B. Longshaw, C. Georgopali, A. Karas, A. Kazeem, G. Palacios-Fabrega, J. A. Vehreschild, Mjgt. Multiplex PCR-based analysis of enteric pathogens in faecal samples from patients with clostridium difficile infection in the randomized, controlled extend study comparing the efficacy of extended-pulsed fidaxomicin with vancomycin therapy. Open forum infectious diseases. 2018;5:S575.

Suppl 8. Wolf J, Kalocsai K, Fortuny C, et al. Safety and Efficacy of Fidaxomicin and Vancomycin in Children and Adolescents with Clostridioides (Clostridium) difficile Infection: A Phase 3, Multicenter, Randomized, Single-blind Clinical Trial (SUNSHINE). Clinical Infectious Diseases. 2019;71(10):2581-8.

Table 3. Summary of included study population characteristics: studies reporting economic modeling analyses

| **Study details** | **Study type** | **Country** | **Study setting** | **Model population** |
| --- | --- | --- | --- | --- |
| Aby *et al.*, 2022(76) | CEA | US | Treatment paradigm for CDI as per 2021 IDSA/SHEA guidelines | Patients presenting with initial CDI diagnosis |
| McDaniel *et al.*, 2022(72) | BIM | US | Clinical/hospital | Adult patients with a positive CDI test |
| Jiang *et al.*, 2022(77) | BIM | US | Clinical/hospital (inpatient and outpatient) | Adult patients with CDI, with none or one prior CDI episode who are treated with mix of fidaxomicin and vancomycin, or vancomycin only |
| Chen *et al.*, 2021(78) | CEA | US | Community | Patients in initial CDI episode |
| Abdali *et al.*, 2020(79) | CEA | UK | Clinical/hospital | Hospitalized patients aged ≥65 years who had at least one CDI recurrence |
| Okumura *et al.*, 2020(80) | CEA | Japan | In-hospital treatment | Patients with CDI who had failed first-line treatment |
| Rajasingham *et al.*, 2020(81) | CEA | US | Treatment paradigm per 2018 IDSA/SHEA guidelines | Patients presenting with initial CDI episode |
| Rubio-Terrés *et al.*, 2019(82) | CEA | Spain | NHS | Patients aged ≥60 years with CDI |
| Cornely *et al.*, 2018(95) | CEA | England | In-hospital | Hospitalized patients ≥60 years old with confirmed CDI |
| Ford *et al.*, 2018(83) | CEA | US | Hospital admission and outpatient clinic for recurrent episodes | Adult inpatients with an initial episode of mild-to-moderate CDI to compare treatments with metronidazole, vancomycin, and fidaxomicin |
| Reveles *et al.*, 2017(84) | CEA | US | In-hospital | Patients receiving initial CDAD treatment and subsequent treatment for up to two recurrences of CDAD who were treated with fidaxomicin or vancomycin |
| Lapointe-Shaw *et al.*, 2016(85) | CEA | US | Inpatient and outpatient healthcare, data from Medicare database | 70-year-old community-dwelling person experiencing their first recurrence of CDI |
| Watt *et al.*, 2016(86) | CEA | Germany | In-hospital | Patients with initial CDI episode |
| Rubio-Terrés *et al.*, 2015(87) | CEA | Spain | In-hospital (severe cases) and community (non-severe) | Patients with initial CDI |
| Wagner *et al.*, 2014(88) | CEA | Canada | Clinical/hospital | Patients with severe CDI (i.e., patients with a white blood cell count 15×109 cells/L or a serum creatinine level ≥1.5 times the premorbid level) who received fidaxomicin or vancomycin treatment |
| Nathwani *et al.*, 2014(89) | CEA | Scotland | In-hospital | Adult patients aged ≥18 years old with CDI |
| Konijeti *et al.*, 2014(90) | CEA | US | Clinical/hospital | Adult patients with CDI |
| Markovic *et al.*, 2014(91) | CEA | Serbia | Clinical/hospital | Patients with colitis induced by *C. difficile* who did not respond to oral therapy with metronidazole |
| Stranges *et al.*, 2013(92) | CEA | US | Clinical/hospital | Patients who received a 10-day course of either fidaxomicin or oral vancomycin for initial episodes of CDI or first recurrence, and were either treated as an outpatient or hospitalized |
| Sclar *et al.*, 2012(93) | CEA | US | In-hospital treatment | Patients with CDAD |
| Brodszky *et al.*, 2014(94) | CEA | Hungary | RCT | Patients with initial and recurrent CDI |

BIM: budget impact model; CEA: cost-effectiveness analysis; CDAD: Clostridioides difficile-associated diarrhea; CDI: Clostridioides difficile infection; IDSA: Infectious Diseases Society of America; L: liter; NHS: National Health Service; RCT: randomized controlled trial; SHEA: Society for Healthcare Epidemiology of America; UK: United Kingdom; US: United States

Table 4. Summary of economic analyses outcomes

| **Author, year** | **Model design** | **Intervention regimen** | **Population detail** | **Key model inputs** | **Outputs** | **Conclusion** |
| --- | --- | --- | --- | --- | --- | --- |
| Abdali *et al*, 2020(79)  CEA | Markov transition state model  2-month cycle  UK NHS perspective  2018 GBP | FMT-NGT; FMT‑colonoscopy; fidaxomicin 200mg twice daily for 10 days; vancomycin 125mg four times daily for 10 days | Hospitalized patients aged ≥65 years who had at least one CDI recurrence  Health states: relapsed, recovered, recurrent CDI, dead | Intervention/comparator costs:   - FMT-NGT: £741.16 - FMT-colonoscopy: £3,006.17 - Vancomycin 250mg: £200.11 - Vancomycin 500mg: £400.23 - Vancomycin tapered: £297.60 - Fidaxomicin: £1,350   Cost of hospital stay per day: £404 | **Cost per patient**:   - FMT-NGT: £8,877 - FMT-colonoscopy: £11,716 - Fidaxomicin: £14,399 - Vancomycin: £17,279   **QALYS**   - FMT-NGT: 0.645 - FMT-colonoscopy: 0.657 - Fidaxomicin: 0.577 - Vancomycin: 0.513 | FMT was most cost‑effective in treating patients with rCDI.  Of the antibiotic interventions, fidaxomicin was less costly and incurred higher QALYs than vancomycin. |
| Aby *et al,* 2022(76)  CEA | Markov health state  1-year  2020 USD  3% annual discount | Treatment strategies including combination of following interventions for:   - Non-severe - Severe - First recurrence - Second or later recurrence:   vancomycin 125mg four times daily, metronidazole 500mg three times daily, for 10 days  fidaxomicin 200mg twice daily, for 10 days  metronidazole IV 500mg three-four times daily for 14 days  FMT | Patients with initial CDI diagnosis  Median age: 67 years | Price (per unit/per course):   - Vancomycin: $12.52/$500.96 - Metronidazole: $0.29/$8.58 - Fidaxomicin: $107.40/$2,148.10 - Metronidazole IV: $0.01/$0.50   Total cost for a full course:   - FMT : $3,230.39   Clinical parameter probabilities:  **Clinical cure (non-severe/severe)**   - Vancomycin: 0.898/0.846 - Metronidazole: 0.783/NA - Fidaxomicin: 0.917/0.800   **Recurrence (non-severe/severe)**   - Vancomycin: 0.215/0.253 - Metronidazole: 0.208/NA - Fidaxomicin: 0.134/0.114 | Optimal treatment combination strategy:  **For non-severe CDI**   - Fidaxomicin   **For severe CDI**   - Vancomycin:   **For recurrent CDI**   - FMT: $27,135   **Combination strategy ICER** per QALY**:** $27,135 | Fidaxomicin was the most effective intervention for non‑severe initial CDI at a WTP threshold of $100,000.  Using FMT earlier was cost-effective for first re­currence of CDI, if the probability of cure is 69% or greater. |
| Brodszky *et al*, 2014*(94)  CEA | Decision tree analysis  Third party payer perspective  EUR (cost year not reported)  Hungary | Fidaxomicin (dose not reported); vancomycin (dose not reported) | Patients with initial and recurrent CDI who were treated with fidaxomicin or vancomycin | Total mean costs:   - Fidaxomicin: €3,987 - Vancomycin: €2,505   Mean recurrence episodes per patient:   - Fidaxomicin: 0.13 - Vancomycin: 0.40 | **ICER** (versus vancomycin):   - Per avoided recurrent episode: €5,520 | Fidaxomicin is more cost-effective than vancomycin, driven by lower associated recurrence rate. |
| Chen *et al*, 2021(78)  CEA | Markov health state transition model  15-day cycle length for initial 6 months, then annual cycles for remainder of lifetime  Societal perspective  2020 USD | EP-fidaxomicin, 200mg twice daily day 1–5, once daily on alternate days from day 7–25; fidaxomicin, 200mg twice daily for 10 days vancomycin 125mg four times daily for 10 days | Patients in initial CDI episode (N=1,000 simulations).  Mean age (range): 62.4 years (49.9–74.9).  Health states: initial CDI episode, treatment failure, treatment success (clinical cure), recurrence, colectomy, sustained clinical cure, post-colectomy, death | Intervention/comparator costs:   - EP-fidaxomicin: $3,613 - Standard fidaxomicin: $3,613 - Vancomycin: $21 | **Total cost** (80% uncertainty interval):   - EP-fidaxomicin: $37,613 (35,795–39,196) - Fidaxomicin: $39,325 (37,113–39,690) - Vancomycin: $39,178 (36,715–39,951)   **ICER** versus vancomycin (80% uncertainty interval):   - EP-fidaxomicin: Dominant (‑$127,102–$16,844) - Fidaxomicin: $495 (‑$1,384–$1,957)   **ICER** versus EP-fidaxomicin (80% uncertainty interval):   - Fidaxomicin: $6,004 ($4,222–$6,162) | EP-fidaxomicin is associated with lowest cost and dominates over vancomycin but is less cost-effective than standard-regimen fidaxomicin at a WTP threshold of $150,000. |
| Cornely *et al.,* 2018(95)  CEA | Cohort-based semi-Markov treatment sequence model  NHS England and Personal Social Services perspective  1-year horizon  2016 GBP  No discount applied | EP-fidaxomicin, 200mg twice daily on days 1–5, once daily on alternate days 7–25; vancomycin 125mg four times daily for 10 days | Hospitalized patients ≥60 years old with confirmed CDI  Health states: CDI episode, clinical outcome of treatment and treatment line, clinical response, CDI recurrence, disease free survival, death | Drug acquisition cost per course:   - EP-fidaxomicin: £1,350 - Vancomycin: £189.24   Hospitalization cost per episode:   - Per 10-day admittance: £8,214.00 - Rescue treatment: £4,107.00   Clinical input parameters:  **Clinical response 2-days post end of treatment**   - EP-fidaxomicin: 78% - Vancomycin: 82.1%   **Recurrence at day 40/55/90**   - EP-fidaxomicin: 1.4%/4.3%/7.2% - Vancomycin: 19.7%/21.1%/22.4% | Drug acquisition costs per patient:   - EP-fidaxomicin: £1,356 - Vancomycin: £260   Hospitalization costs per patient:   - EP-fidaxomicin: £10,815 - Vancomycin: £11,459   AE-management associated costs per patient:   - EP-fidaxomicin: £694 - Vancomycin: £1,199   EP-fidaxomicin treatment for initial CDI episode was associated with cost savings of £53 per patient and a gain of 0.0229 QALYs per patient compared with vancomycin treatment. | Higher drug acquisition costs associated with EP‑fidaxomicin are offset by lower hospitalization and AE-management costs. |
| Ford *et al,* 2018(83)  CEA | Decision analysis  Healthcare system perspective  2016 USD | Fidaxomicin 200mg twice daily for 10 days; metronidazole, 500mg three times daily for 10 days; vancomycin, 125mg four times daily for 10 days | Hospitalized adults (≥18 years) with an initial episode of mild-to-moderate CDI  Health states: Cure, nonresponse to initial treatment, sustained cure, recurrence, persistent nonresponse and complicated CDI, death | Cost of treatment course:   - Fidaxomicin: $4,188 - Vancomycin: $37 - Metronidazole: $22 - FMT: $1,207   Cost of healthcare resource use:   - Outpatient visit: $108 - Readmission: $11,176 - Colectomy: $9,315 - Added length of stay: $9,770 - Persistent CDI post-FMT: $16,701 | **ICER** per 1% gain in cure, (versus fidaxomicin)   - Vancomycin: $2,828.69 - Metronidazole: $1,540.23   Optimal combination treatment strategies with each intervention for initial treatment, **cost/sustained cure rate** per patient:   - Fidaxomicin, fidaxomicin, vancomycin taper: $4,470.75/**99.12%** - Vancomycin, vancomycin taper, fidaxomicin: $**449.66**/98.68% - Metronidazole, fidaxomicin, vancomycin taper: $600.46/98.15% | Treatment with vancomycin or metronidazole for initial CDI episode was less costly but less effective than fidaxomicin treatment. |
| Jiang *et al*, 2022(77)  BIM | Cohort-based decision analysis  1 year horizon  US Healthcare provider perspective  2019 USD | Fidaxomicin 200mg twice daily for 10 days; vancomycin 125mg four times daily for 10 days. | Adult patients with CDI (≥18 years), with no or one prior CDI episode (N=10,000 annual simulations) | Intervention cost:   - Fidaxomicin 10-day course: catalog $3,865.80; discounted $2,319.48.   *Assumed a 40% discount, consistent with previous economic analysis*  Comparator cost:   - Vancomycin 10-days course: catalog and discounted $136.30   CDI recurrence rate:  **No prior episode/one prior episode**   - Fidaxomicin: 11.7%/19.7% - Vancomycin: 22.6% /35.5% | Total potential savings for patients with **no** prior CDI episode:   - At hospital level: $1,105 - Per treated patient: $14 - Per admitted patient: $0.11   Total potential savings for patients with **one** prior CDI episode:   - At hospital level: $1,150 - Per treated patient: $74 - Per admitted patient: $0.12 | Fidaxomicin use is associated with hospital savings due to reduced CDI recurrence and superior sustained clinical response. |
| Konijeti *et al,* 2014(90)  CEA | Decision analysis  1-year horizon  Societal perspective  2012 USD | FMT-colonoscopy; FMT‑enema; FMT‑duodenal, metronidazole 500mg three times daily for 10 days; vancomycin 125/500mg four times daily for 10 days or 6‑week taper, fidaxomicin 200mg twice daily for 10 days | Adult patients with CDI  Median age: 65 years  Health states: healthy, mild-moderate CDI, severe CDI, recurrent disease, post‑colectomy, death | Intervention cost per course:   - Metronidazole: $22–$32 - Vancomycin: $250–$680, taper: $850 - Fidaxomicin: $2,800 | Cost/QALY for interventiions for the management of recurrent CDI (Base Case):   - Vancomycin: $2,912/0.8580 - FMT-colonoscopy: $3,149/0.8719 - FMT enema: $4,090/0.8543 - FMT duodenal infusion: $4,208/0.8553 - Metronidazole: $3,941/0.8292 - Fidaxomicin: $4,261/0.8653   **ICER**  FMT via colonoscopy dominated against all other interventions. | FMT via colonoscopy was the most cost-effective strategy at a WTP of $50,000 per QALY. |
| Lapointe-Shaw *et al.,* 2016(85)  CEA | Decision analysis with Markov processes  Lifetime horizon  6-week cycle  Canadian NHS (Ontario Ministry of Health and Long-Term Care) perspective  2014 CAD | Metronidazole 500mg three times daily for 14 days; vancomycin 125mg four times daily for 14 days; fidaxomicin 200mg twice daily for 10 days;  FMT by enema, NGT, or colonoscopy (all FMT regimen included 14 days of oral vancomycin) | 70-year-old community-dwelling person experiencing their first recurrence of CDI (N=1,000 simulations)  Health states: Recurrence during first cycle, then patients were cured, experienced further recurrence, or died | Cost of intervention course:   - Metronidazole: $20–$39, - Vancomycin: $347–$505, - Fidaxomicin: $2,405. - FMT by enema: $7,690 - FMT by NGT: $953 - FMT by colonoscopy: $4,760 | Outcomes per 1,000 patients (post-initial recurrences/hospitalizations/ CDI‑related deaths/mean life years):   - Vancomycin: 636/284/119/14.46 - Metronidazole: 583/275/115/14.78 - Fidaxomicin: 458/253/106/14.90 - FMT enema: 340/233/98/15.04 - FMT-NGT: 426/247/08/14.87 - FMT-colonoscopy: 144/199/84/15.26 | FMT by colonoscopy was the most effective and less costly intervention. |
| Markovic *et al,* 2014(91)  CEA | Markov model  3-month horizon  Healthcare payer perspective (Republic Institute for Health Insurance Serbia) | Fidaxomicin, 200mg twice daily for 10 days; vancomycin 125mg four times daily for 10 days | Patients with colitis induced by *C. difficile* who did not respond to oral therapy with metronidazole (N=1,000 iterations)  Health states: colitis, relapsed colitis by *C. difficile,* subtotal colectomy with recovery, subtotal colectomy with death outcome, fulminant colitis treatment response, death | Intervention cost per course:   - Fidaxomicin: $2,800 (not provided in RSD) - Vancomycin: 5,840 RSD   Total cost per 3-month cycle per patient:  **Fidaxomicin**   - 48,106.19 RSD (SD: 118.07)   **Vancomycin**   - 25,872.85 RSD (SD: 41.44) | Number of patients with CDI‑induced colitis undergoing total colectomy:   - Fidaxomicin: 0.014 (SD: 0.00) - Vancomycin: 0.016 (SD: 0.005)   Mortality rate:   - Fidaxomicin: 0.05 (SD: 0.00) - Vancomycin: 0.057 (0.00)   **ICER** (versus vancomycin):   - Per life saved: 2,977,621.51 RSD (SD: 29,733.10) - Per avoided colectomy: 10,175,146.97 RSD (SD: 101,310.61) | Fidaxomicin is a cost-effective treatment for colitis versus vancomycin for treating patients with CDI‑induced colitis per life gained, lying below the threshold value for statistical life of 53.3 million RSD.  Fidaxomicin use to prevent one total colectomy is above the threshold and is not a cost‑effective strategy. |
| McDaniel *et al,* 2022(72)  BIM | Budget impact  2020 USD | Pre-versus post-implementation of fidaxomicin as first-line treatment for first or second episode of CDI | Adult patients (≥18 years) with a positive CDI test (N=100) | **Intervention uptake:**   - Pre-imp. period: metronidazole (1.6%), vancomycin (41.7%), fidaxomicin (56.7%) - Post-imp.: metronidazole (47.8%), vancomycin (50.5%), fidaxomicin (1.6%)   Incremental difference from pre- to post- implementation, **clinical inputs per 100 patients**:   - CDI recurrence: -12 - Sustained response: 17 - CDI-related admission: -10 - Readmission length of stay: -50 | **Total direct cost savings (index + readmission):** $222,895  **Savings in post-implementation period:**   - Per additional patient with sustained response: $5,548 - Per CDI-related readmission avoided: $9,432 - Per bed-day avoided: $1,886 | Increased fidaxomicin use was associated with improved clinical outcomes and hospital cost‑savings. |
| Nathwani *et al,* 2014(89)  CEA | Time-dependent Markov model  10-day cycle  1-year horizon  NHS Scotland perspective  2010/11 GBP | Fidaxomicin 200mg twice daily for 10 days; vancomycin 125mg four times daily for 10 days | Adult patients (aged ≥18 years) with CDI  Patient subgroups:   - Severe CDI - 1^st^ recurrence   Health states: CDI, CDI cured, CDI cured after failure, CDI treatment failed, death | Cost of intervention:   - Fidaxomicin: £1,350 - Metronidazole: £2.17 - Vancomycin: £189–757, taper £407 - Last resort therapy: £397   Cost of severe CDI complication: £9,915  Cost of GP visit (clinic/home): £53/120  Cost of admission per day:   - General ward: £430.87 - Infectious ward: £606 - Intensive care: £2,044 | Key mean costs per patient (fidaxomicin/vancomycin):  **Severe CDI**   - Drug acquisition: £2,567/£571 - Hospitalization: £13,600/£11,793 - Total: £14,515/£14,344   **1^st^ recurrence**   - Drug acquisition: £3,630/£800 - Hospitalization: £12,742/£15,928 - Total: £16,535/£16,926   Mean QALYs (fidaxomicin/vancomycin):  **Severe CDI**   - 0.715/0.705   **1^st^ recurrence**   - 0.711/0.692   Cost per recurrence avoided:   - For severe CDI: £400 - For 1^st^ recurrence: -£518   **ICER** (versus vancomycin)   - For severe CDI: £16,529 - For 1^st^ recurrence: Dominant | Fidaxomicin is cost-effective at WTP of £30,000 per QALY for use in patients severe CDI and dominant versus vancomycin in patients experiencing first CDI recurrence.  Higher acquisition costs associated with fidaxomicin were offset by reduced hospitalization costs. |
| Okumura *et al*, 2020(80)  CEA | Cohort-based semi-Markov model  1 year  2018 JPY  No discount applied | Fidaxomicin 200mg twice daily for 10 days; vancomycin 125mg four times daily for 10 days' | Hospitalized adult patients with CDI who had failed first-line treatment with metronidazole (N=100)  Mean age: 74.5 years  Patient subgroups:   - Primary CDI - Non-severe CDI - Severe CDI - Concomitant antibiotics - Age ≥65 years   Health states: CDI, CDI cured, CDI recurrence, treatment failure, death | Clinical parameters:  Cure rate/ recurrence rate   - Fidaxomicin: 96.7%/ 5.2% - Vancomycin: 89.7%/ 7.3%   Drug acquisition cost per treatment (JPY):   - Fidaxomicin: 78,876 - Vancomycin 125mg: 25,366 - Vancomycin 250mg: 50,732 - Vancomycin 500mg: 101,464 - Vancomycin taper (8 weeks): 33,220 - Metronidazole: 1,444 | **ICER** (versus vancomycin)   - Base case: 5,715,183JPY per QALY gained.   Scenario analyses when used as first‑line treatment for CDI:   - All patients: 867,081 - Primary CDI: 2,144,621 - Non-severe CDI: 1,210,385 - Severe CDI: 4,070,193 - Concomitant antibiotics: Dominant - Age ≥65 years: 368,530 | Higher acquisition cost of fidaxomicin is partly offset by reduced costs associated with hospitalization, GP visit, and complications, driven by fewer recurrences.  When used as first-line treatment fidaxomicin is cost‑effective at a WTP threshold of 5,000,000JPY across all scenarios (primary episode, non-severe, severe, receiving concomitant antibiotics, and ≥65 years). |
| Rajasingham *et al*, 2020(81)  CEA | Markov model  2-month cycle; 1‑year horizon  Healthcare payer perspective  2018 USD  3% annual discount | Fidaxomicin 200mg twice daily for 10 days  vancomycin 125 mg four times daily for 10 days  metronidazole 500mg three times daily for 10 days (initial CDI non-severe only)  vancomycin taper (recurrences only)  FMT, vancomycin+rifaximin (second or higher recurrences) | Patients presenting with initial CDI episode  Median age: 67 years  Health states: Initial CDI, non-severe CDI, first CDI recurrence, second or higher recurrence | Total intervention costs per course:   - Fidaxomicin: $1,767.20 - Vancomycin: $14.08 - Metronidazole: $8.76 - Metronidazole IV: $39.12 | Across four scenarios (non‑severe, severe, first recurrence, second or higher recurrence), the **most effective treatment strategy** was:   - Fidaxomicin for non-severe CDI - Vancomycin for severe CDI - Fidaxomicin for first recurrence - FMT for second or higher recurrence   The outlined strategy had an ICER of $31,751 per QALY and was cost‑effective at a WTP of $100,000 per QALY gained. | Fidaxomicin is a cost-effective treatment choice for initial non‑severe CDI and first recurrence.  Treatment was primarily cost-effective due to clinical parameters: deaths avoided, reduced utility loss, and hospitalization costs associated with recurrent CDI. |
| Reveles *et al.,* 2017(84)  CEA | Decision analysis  Hospital perspective  2014 USD | Fidaxomicin 200mg twice daily for 10 days; vancomycin, 125mg four times daily for 10 days | Patients receiving treatment for initial and ≤2 recurrences of CDAD.  Model considered patient subgroups:   - General population - ≥65 years - Cancer patients - Concomitant antibiotic use - Patients at stage 3/4 CKD   Health states: cured, failure, first recurrence or no recurrence | Daily drug acquisition cost:   - Fidaxomicin: $235 - Vancomycin 125mg: $20   Cost of additional hospital days in cases with treatment failure: $5,573  Attributable hospitalization cost of initial/recurrent CDAD: $11,145/$12,627 | CDAD treatment cost among general population (initial/1^st^ recurrence/2^nd^ recurrence/overall):   - Fidaxomicin: $13,171**/$1,088/$183**/$14,442 - Vancomycin: $12,058/$1,818/$303/$14,179   Cost savings for CDAD treatment across initial, 1^st^, and 2^nd^ recurrent CDAD hospitalization per patient (i.e., fidaxomicin–vancomycin = savings):   - ≥65 years: $-243 - Patients at stage 3/4 CKD: $‑371 - Cancer patients: **$616** - Concomitant antibiotic use: **$312** | Cost savings associated with fidaxomicin due to lower recurrence rates offset most of the drug costs.  Fidaxomicin is cost saving for patients with cancer and those receiving concomitant antibiotics. |
| Rubio-Terrés *et al*, 2019 (82)  CEA | Cohort-based Markov model  5-day cycle; 1‑year horizon  Spanish NHS perspective  2017 EUR  No discount | EP-fidaxomicin, 200mg twice daily on days 1–5, once daily on alternate days 7–25;  vancomycin 125mg four times daily for 10 days | Patients aged ≥60 years with CDI  Health states: initial CDI episode, treatment success, treatment failure, CDI recurrence, sustained clinical cure, and death | Total intervention costs per course:   - EP-fidaxomicin: €1,387.50 - Vancomycin (125mg): €34.50 - Vancomycin (250mg/500mg): €69.00 - Vancomycin taper: €74.00 | **Base case/probabilistic analysis**  **Cost**   - EP-fidaxomicin: €10,046/€10,051 - Vancomycin: €10,693/€10,697   **QALY**   - EP-fidaxomicin: 0.638/0.635 - Vancomycin: 0.594/0.592   **ICER**  In base case and probabilistic analyses EP-fidaxomicin dominates.  EP-fidaxomicin is 99% likely to be cost-effective compared to vancomycin at a WTP threshold of €20,000. | First-line treatment with EP-fidaxomicin is more cost‑effective than vancomycin and meets standard Spanish WTP thresholds |
| Rubio-Terrés *et al.,* 2015(87)  CEA | Markov health state  NHS Spanish hospital perspective  1-year horizon  2013 EUR | Fidaxomicin 200mg twice daily for 10 days; vancomycin 125mg four times daily for 10 days | Patients with initial CDI in‑hospital and community  Patient subgroups:   - Cancer - Concomitant antibiotic - Renal impairment | Daily drug acquisition costs:   - Fidaxomicin: €138.75 - Vancomycin: €3.45–€6.90 - Vancomycin taper: €74.46   Cost of healthcare resource use per day or visit:   - General ward: €604 - ICU: €1,134.46 - Primary care GP visit: €35.54 - Primary care GP home visit: €46.25 | **Incremental QALYs/total costs** (fidaxomicin–vancomycin=difference or savings):   - Cancer patients: 0.016/-€2,397 - Concomitant antibiotic use: 0.014/-€1,452 - Renal impairment patients: 0.013/-€1,432   **ICER** (versus vancomycin):   - Cancer patients: Dominant - Concomitant antibiotic use: Dominant - Renal impairment patients: Dominant | Fidaxomicin was more effective and less costly than vancomycin among patients with cancer or renal impairment, and those receiving concomitant antibiotic at a WTP threshold of €30,000. |
| Sclar *et al.,* 2012(93)  CEA | Epidemiological model to estimate market price value  2011 USD  Healthcare perspective | Fidaxomicin 200mg twice daily for 10 days; vancomycin 125mg or 250mg four times daily for 10 days | Patients with CDAD (N=336,565) | Wholesale acquisition cost for intervention per day:   - Fidaxomicin: $280 - Vancomycin (injectable administered orally 125mg/capsules 125mg/capsules 250mg): $6/$106/$196   Healthcare resource utilization costs were derived from the 2009 US Healthcare Cost and Utilization Project Nationwide Inpatient Sample. | Savings/loss per day for **primary** CDAD (i.e., fidaxomicin–vancomycin=savings or loss):   - Hospital: -$56.37–$133.63 - Ambulatory: -$174.00– -$84.00 - Health system: $-916.57–$683.43   Savings/loss per day for **secondary** CDAD (i.e., fidaxomicin–vancomycin=savings or loss):   - Hospital: $476.43–$666.43 - Ambulatory: -$174.00– -$84.00 - Health system: $211.29–$1,411.29 | In most primary-case scenarios, and all secondary-cases of CDAD examined, in‑hospital fidaxomicin use was cost-saving compared to vancomycin. |
| Stranges *et al,* 2013(92)  CEA | Decision analysis  23-year horizon  USD  Payer perspective  3% discount | Fidaxomicin 200mg twice daily for 10 days; vancomycin 125mg four times daily for 10 days | Patients experiencing initial CDI episode of CDI or first recurrence, treated as an outpatient or hospitalized  Age; 59.9 years  Patient subgroups:   - Inpatient - Outpatient - Mild-to-moderate cure - Severe cure - Concomitant antimicrobial - NAP1/B1/027 strains   Health states: cure, recurrence, no recurrence, no cure, surgery, or no surgery, alive, death | Intervention cost per course:   - Fidaxomicin: $2,800 - Vancomycin (outpatient/125mg/500mg/taper): $1,161/$20/$80/$812 - Metronidazole (500mg/IV): $114/$75   Cost of hospitalization (per day): $10,793 ($1,542)  Clinical parameter base case values (inpatient/outpatient/mild-moderate cure/severe cure/concomitant antimicrobial/NAP1/B1/027 strain):  **Clinical cure**   - Fidaxomicin: 0.814/0.975/0.92/0.821/0.9/0.787 - Vancomycin: 0.781/0.975/0.839/0.886/0.794/0.807   (inpatient/outpatient/previous episode/mild‑moderate cure/severe cure/concomitant antimicrobial/NAP1/B1/027 strain):  **Recurrence**   - Fidaxomicin: 0.176/0.128/0.214/0.168/0.13/0.169/0.271 - Vancomycin: 0.274/0.227/0.312/0.244/0.266/0.292/0.209 | **ICER** (versus vancomycin):   - Base case: $67,576   Initial episode   - Outpatient: $38,571 - Inpatient: $75,111   Disease severity   - Mild-to-moderate: $31,020 - Severe: $352,994 - NAP1/B1/027 strain: Dominated - Concomitant antimicrobials: $1,487 (versus metronidazole) - Mild-to-moderate disease: $40,513 | Fidaxomicin is more cost-effective than vancomycin and metronidazole in several treatment scenarios at a WTP threshold of $100,000 per QALY.  Cost-effectiveness of fidaxomicin was driven by higher clinical cure and reduced recurrence rates than vancomycin. |
| Wagner *et al.,* 2014(88)  CEA | Decision tree analysis  Healthcare system perspective  2 months (post-onset initial episode)  CAD (cost year not specified) | Fidaxomicin 200mg twice daily for 10 days; vancomycin 125mg four times daily for 10 days | Patients with severe CDI (i.e., white blood cell count 15×109 cells/L or a serum creatinine level ≥1.5 times the premorbid level) (N=1,000 simulations)  Health states: Initial cure, failure, recurrence, no recurrence | Cost of intervention per day:   - Fidaxomicin: $220.00 - Vancomycin: $20.72   Cost of outpatient consultation: $65.90  Cost of colectomy physician fees: $1,700 | **Clinical outcomes for 1000 patients with severe CDI** treated with fidaxomicin/vancomycin:  Severe CDI   - Initial cure: 813/813 - Recurrence: 93/230 - Hospitalized for recurrence: 18/44   First recurrent CDI   - Initial cure for 1^st^ recurrence: 926/926 - 2^nd^ recurrences: 188/301 - Hospitalized for recurrence: 36/58   **Incremental cost** (fidaxomicin–vancomycin):  For severe CDI:   - Initial treatment: $1,992,800 - Recurrent treatment: -$182,226   (Total cost: $1,810,574)   - Per recurrence avoided: $13,202   For patients with first recurrent CDI:   - 1^st^ recurrence treatment: $1,992,800 - 2^nd^ recurrence treatment: -$69,863   (Total cost; $2,062,663)   - Per 2^nd^ recurrence avoided: $18,190 | Fidaxomicin was associated with fewer recurrences and recurrence-related hospitalizations but was more costly than vancomycin. |
| Watt *et al,* 2016(86)  CEA/BIM | Semi-Markov model  10-day cycle  1-year horizon  German payer perspective  2014 EUR  No discount | Fidaxomicin 200mg twice daily for 10 days; vancomycin 125mg four times daily for 10 days | Hospitalized patients with initial CDI episode (N=100)  Six patient subgroups:   - ≥1 recurrence - Severe CDI - Concomitant antibiotics - ≥65 years - Cancer - Renal impairment   Health states: initial episode, treatment failure, clinical cure, sustained clinical cure, recurrence, death | Cost of intervention:   - Fidaxomicin: €1,387 - Vancomycin: €61. - Rescue treatment (FMT): €1,500.   Hospitalization costs on general ward per day: €348 | **ICER** per QALY gained **(**versus vancomycin):   - ≥1 recurrence: €43,900 - Severe CDI: €34,800 - Concomitant antibiotics: €30,700 - ≥65 years: €44,500 - Cancer: Dominant - Renal impairment: €26,900   **Budget impact** (fidaxomicin–vancomycin=impact)   - ≥1 recurrence: €461 - Severe CDI: €396 - Concomitant antibiotics: €291 - ≥65 years: €461 - Cancer: -€806 - Renal impairment: €334 | Fidaxomicin is cost-effective intervention for first-line CDI treatment at WTP threshold of €50,000 per QALY.  Fidaxomicin is a more cost‑effective treatment than vancomycin among patients with cancer.  Cost-effectiveness was driven by savings per bed-day saved per recurrence avoided. |

*Limited detail of model characteristics due to being a conference abstract.

BIM: budget impact model; CAD: Canadian Dollars; CDAD: Clostridioides difficile-associated diarrhea; CDI: Clostridioides difficile infection; CEA: cost-effectiveness analysis; CKD: chronic kidney disease; EP-fidaxomicin: extended-pulsed fidaxomicin; EUR: Euro; FMT: fecal microbiota transplantation; GBP: British Pound Sterling; ICER: incremental cost-effectiveness ratio; IV: intravenous; JPY: Japanese Yen; mg: milligram; NA: not applicable; NGT: nasogastric tube; NHS: National Health Service; QALY: quality adjusted life-year; RSD: Serbian Dinar; SD: standard deviation; UK: United Kingdom; US: United States; USD: US Dollar; WTP: willingness to pay

Table 5. JBI critical appraisal results for cohort studies

For Supplementary references see at end of Supplementary Table 2.

|  | **Were the two groups similar and recruited from the same population?** | **Were the exposures measured similarly to assign people to both exposed and unexposed groups?** | **Was the exposure measured in a valid and reliable way?** | **Were confounding factors identified?** | **Were strategies to deal with confounding factors stated?** | **Were the groups/participants free of the outcome at the start of the study (or at the moment of exposure)?** | **Were the outcomes measured in a valid and reliable way?** | **Was the follow-up time reported and sufficient to be long enough for outcomes to occur?** | **Was follow-up complete, and if not, were the reasons to loss to follow-up described and explored?** | **Were strategies to address incomplete follow-up utilized?** | **Was appropriate statistical analysis used?** | **Overall appraisal** |
| --- | --- | --- | --- | --- | --- | --- | --- | --- | --- | --- | --- | --- |
| Biggs *et al.* 2019(39) | Yes | Yes | Yes | No | NA | Yes | Yes | Yes | NR | NA | Yes | Low risk of bias |
| Bouza *et al.* 2017(48) | Yes | Yes | Yes | No | NA | Yes | Yes | Yes | Yes | NA | Yes | Low risk of bias |
| Cho *et al.* 2018(40) | NA | NA | Yes | No | NA | Yes | Yes | Yes | Yes | NA | Yes | Low risk of bias |
| Conrad *et al.* 2022(54) | Yes | NA | Yes | No | No | Yes | Yes | Yes | Yes | NA | Yes | High risk of bias |
| Dubberke *et al.* 2022(21) | Yes | NA | Yes | No | NA | NA | Yes | Yes | NR | NA | Yes | High risk of bias |
| Dubberke *et al.* 2022(Suppl 2) | Yes | Yes | Yes | No | NA | Yes | Yes | Yes | NR | NA | Yes | Low risk of bias |
| Eiland *et al.* 2015(22) | Unclear | Unclear | Unclear | No | NA | Yes | Unclear | NR | NR | NA | Unclear | High risk of bias |
| Enoch *et al.* 2018(56) | Yes | Yes | Yes | No | NA | Yes | Yes | Yes | Yes | NA | Yes | Low risk of bias |
| Escudero-Sanchez *et al.* 2021(23) | Yes | Yes | Yes | Yes | No | Yes | Yes | Yes | Yes | NA | Yes | Low risk of bias |
| Fehér *et al.* 2017(24) | Yes | Yes | Yes | Yes | Yes | Yes | Yes | Yes | NR | NA | Yes | Low risk of bias |
| Filippidis *et al.* 2022(Suppl 3) | Yes | Yes | Yes | No | NA | No | Yes | Yes | NR | NA | Yes | Low risk of bias |
| Gallagher *et al.* 2015(51) | Yes | Yes | Yes | No | NA | Yes | Yes | Yes | NR | NA | Yes | Low risk of bias |
| Gentry *et al.* 2019(25) | Yes | Yes | Yes | Yes | Yes | Yes | Yes | Yes | Yes | NA | Yes | Low risk of bias |
| Giancola *et al.* 2018(52) | Yes | Yes | Yes | No | NA | Yes | Yes | Yes | Yes | NA | Yes | Low risk of bias |
| Goldenberg *et al.* 2015(74) | Yes | Yes | Yes | Yes | No | Yes | Yes | Yes | Yes | NA | Yes | Low risk of bias |
| Gomez Delgado *et al.* 2022(55) | Yes | Yes | Yes | No | NA | NA | Yes | NR | NA | NA | Yes | High risk of bias |
| Green *et al.* 2021(42) | Yes | Unclear | Unclear | NR | NA | Yes | Unclear | Yes | Unclear | NA | Yes | High risk of bias |
| Guery *et al.* 2021(46) | Yes | Yes | Yes | No | NA | Yes | Yes | Yes | Yes | NA | Yes | Low risk of bias |
| Hall *et al.* 2022(Suppl 4) | Yes | Yes | Yes | Yes | No | Yes | Yes | Yes | Yes | NA | Yes | Low risk of bias |
| Jon *et al.* 2021(67) | NA | NA | Yes | No | NA | NA | Yes | NA | NA | NA | No | Low risk of bias |
| McDaniel *et al.* 2022(72) | Yes | Yes | Yes | Yes | NA | NA | Yes | Yes | NA | NA | Yes | Low risk of bias |
| Mekideche *et al.* 2018(28) | Yes | Yes | Yes | Yes | No | Yes | Yes | Yes | Yes | NA | Yes | Low risk of bias |
| Novotný *et al.* 2018(57) | Yes | Yes | Yes | No | NA | Yes | Yes | Yes | Yes | No | Yes | Low risk of bias |
| Patel *et al.* 2020(30) | Yes | Yes | Yes | Yes | Yes | Yes | Yes | Yes | Yes | NA | Yes | Low risk of bias |
| Penziner *et al.* 2014(75) | NA | NA | Yes | No | NA | Yes | Yes | Yes | Yes | NA | Yes | Low risk of bias |
| Polivkova *et al.* 2021(31) | Yes | NA | NA | Yes | Yes | Yes | Yes | Yes | NR | NR | Yes | Low risk of bias |
| Rinaldi *et al.* 2021(53) | Yes | Yes | Yes | Yes | Yes | Yes | Yes | Yes | Yes | NA | Yes | Low risk of bias |
| Spiceland *et al.* 2018(32) | Unclear | Yes | Yes | No | NA | Yes | Yes | Yes | NA | NA | Yes | Low risk of bias |
| Tariq *et al.* 2017(Suppl 6) | NA | NA | Yes | No | NA | Yes | Yes | Yes | Yes | NA | Yes | Low risk of bias |
| Thorpe *et al.* 2019(71) | NA | NA | Yes | No | NA | NA | Yes | NA | NA | NA | No | Low risk of bias |
| Tieu *et al.* 2018(33) | NA | NA | Yes | No | NA | Yes | Yes | Yes | Yes | NA | Yes | Low risk of bias |
| Yen *et al.* 2022(45) | Yes | Unclear | Unclear | NR | NA | Yes | Unclear | Yes | Unclear | NA | Yes | High risk of bias |

NA: not applicable, NR: not reported.

Table 6. JBI critical appraisal results for randomized controlled trial studies

For Supplementary references see at end of Supplementary Table 2.

|  | **Was true randomization used for assignment of participants to treatment groups?** | **Was allocation to treatment groups concealed?** | **Were participants blind to treatment assignment?** | **Were those delivering treatment blind to treatment assignment?** | **Were outcomes assessors blind to treatment assignment?** | **Were treatment groups treated identically other than the intervention of interest?** | **Was follow-up complete and if not, were differences between groups in terms of their follow-up adequately described and analyzed?** | **Were participants analyzed in the groups to which they were randomized?** | **Were outcomes measured in the same way for treatment groups?** | **Were outcomes measured in a reliable way?** | **Was appropriate statistical analysis used?** | **Was the trial design appropriate, and any deviations from the standard RCT design (individual randomization, parallel groups) accounted for in the conduct and analysis of the trial?** | **Overall appraisal** |
| --- | --- | --- | --- | --- | --- | --- | --- | --- | --- | --- | --- | --- | --- |
| Aoki *et al.* 2019(64) | Yes | Yes | Yes | Yes | NR | Yes | Yes | Yes | Yes | Yes | Yes | Yes | Low risk of bias |
| Cornely *et al.*, 2012 (Suppl 1) | Yes | Yes | Yes | Yes | NR | Yes | Yes | Yes | Yes | Yes | Yes | Yes | Low risk of bias |
| Cornely *et al.*, 2019(49) | Yes | No | No | No | No | Yes | Yes | Yes | Yes | Yes | Yes | Yes | High risk of bias |
| Eyre *et al.*, 2014(50) | Yes | Yes | Yes | Yes | Unclear | Yes | Yes | Yes | Yes | Yes | Yes | Yes | Low risk of bias |
| Guery *et al.*, 2018(26) | Yes | No | No | No | NR | Yes | Yes | Yes | Yes | Yes | Yes | Yes | High risk of bias |
| Hvas *et al.*, 2019(27) | Yes | No | No | No | No | Yes | Yes | Yes | Yes | Yes | Yes | Yes | High risk of bias |
| Lee *et al.*, 2016(43) | Yes | Yes | Yes | Yes | NR | Yes | Yes | Yes | Yes | Yes | Yes | Yes | Low risk of bias |
| Mikamo *et al.*, 2018(29) | Yes | Yes | Yes | Yes | Yes | Yes | Yes | Yes | Yes | Yes | Yes | Yes | Low risk of bias |
| O'Gorman *et al.*, 2018(47) | Yes | No | No | No | No | Yes | Yes | Yes | Yes | Yes | Yes | Yes | High risk of bias |
| Wilcox *et al.*, 2018 (Suppl 7) | NR | NR | NR | NR | NR | Unclear | NR | Yes | Yes | Yes | Yes | Unclear | High risk of bias |
| Wilcox *et al.*, 2018(34) | NR | NR | NR | NR | NR | Yes | Unclear | Yes | Yes | Yes | Yes | Yes | High risk of bias |
| Wolf *et al.*, 2020(36) | Yes | Yes | No | Yes | Yes | Yes | Yes | Yes | Yes | Yes | Yes | Yes | Low risk of bias |

NR: not reported.

Table 7. JBI critical appraisal results for economic evaluation studies

|  | **Is there a well-defined question?** | **Is there comprehensive description of alternatives?** | **Are all important and relevant costs and outcomes for each alternative identified?** | **Has clinical effectiveness been established?** | **Are costs and outcomes measured accurately?** | **Are costs and outcomes valued credibly?** | **Are costs and outcomes adjusted for differential timing?** | **Is there an incremental analysis of costs and consequences?** | **Were sensitivity analyses conducted to investigate uncertainty in estimates of cost or consequences?** | **Do study results include all issues of concern to users?** | **Are the results generalizable to the setting of interest in the review?** | **Overall appraisal** |
| --- | --- | --- | --- | --- | --- | --- | --- | --- | --- | --- | --- | --- |
| Abdali *et al.* 2020(79) | Yes | Yes | Yes | Yes | Yes | Yes | No | Yes | Yes | Yes | Yes | Low risk of bias |
| Aby *et al.*, 2022(76) | Yes | Yes | Yes | Yes | Yes | Yes | No | Yes | Yes | Yes | Yes | Low risk of bias |
| Brodszky *et al.*, 2014(94) | Yes | Yes | NR | Yes | NR | NR | No | Yes | NR | No | Unclear | High risk of bias |
| Chen *et al.*, 2021(78) | Yes | NA | NA | Yes | Yes | Yes | Yes | Yes | Yes | Yes | Yes | Low risk of bias |
| Cornely *et al.*, 2018(95) | Yes | Yes | Yes | Yes | Yes | Yes | Yes | Yes | Yes | Yes | Yes | Low risk of bias |
| Ford *et al.*, 2018(83) | Yes | Yes | Yes | Yes | Yes | Yes | No | Yes | Yes | Yes | Yes | Low risk of bias |
| Jiang *et al.*, 2022(77) | Yes | Yes | Yes | Yes | Yes | Yes | Yes | Yes | Yes | Yes | Yes | Low risk of bias |
| Konijeti *et al.*, 2014(90) | Yes | Yes | Yes | Yes | Yes | Yes | No | Yes | Yes | Yes | Yes | Low risk of bias |
| Lapointe-Shaw *et al.*, 2016(85) | Yes | Yes | Yes | Yes | Yes | Yes | Yes | Yes | Yes | Yes | Yes | Low risk of bias |
| Markovic *et al.*, 2014(91) | Yes | Yes | Yes | Yes | Yes | Yes | No | Yes | Yes | Yes | Yes | Low risk of bias |
| Nathwani *et al.*, 2014(89) | Yes | Yes | Yes | Yes | Yes | Yes | Yes | Yes | Yes | Yes | Yes | Low risk of bias |
| Okumura *et al.*, 2020(80) | Yes | Yes | Yes | Yes | Yes | Yes | No | Yes | Yes | Yes | Yes | Low risk of bias |
| Olivares *et al.*, 2022(73) | Yes | NA | NA | No | Unclear | Unclear | No | No | No | No | No | High risk of bias |
| Rajasingham *et al.*, 2020(81) | Yes | Yes | Yes | Yes | Yes | Yes | Yes | Yes | Yes | Yes | Yes | Low risk of bias |
| Reveles *et al.*, 2017(84) | Yes | Yes | Yes | Yes | Yes | Yes | No | No | Yes | Yes | Yes | Low risk of bias |
| Rubio-Terrés *et al.*, 2015(87) | Yes | Yes | No | Yes | Yes | Yes | No | Yes | Yes | No | Yes | Low risk of bias |
| Rubio-Terrés *et al.*, 2019(82) | Yes | Yes | Yes | Yes | Yes | Yes | Yes | Yes | Yes | Yes | Yes | Low risk of bias |
| Sclar *et al.*, 2012(93) | Yes | Yes | Yes | Yes | Yes | Yes | No | No | No | No | Yes | Low risk of bias |
| Stranges *et al.*, 2013(92) | Yes | Yes | Yes | Yes | Yes | Yes | Yes | Yes | Yes | Yes | Yes | Low risk of bias |
| Wagner e*t al.*, 2014(88) | Yes | Yes | Yes | Yes | Yes | Yes | No | Yes | Yes | Yes | Yes | Low risk of bias |
| Watt *et al.*, 2016(86) | Yes | Yes | Yes | Yes | Yes | Yes | No | Yes | Yes | No | Yes | Low risk of bias |

NA: not applicable

Table 8. JBI critical appraisal results for systematic review studies

|  | **Is the review question clearly and explicitly stated?** | **Were the inclusion criteria appropriate for the review question?** | **Was the search strategy appropriate?** | **Were the sources and resources used to search for studies adequate?** | **Were the criteria for appraising studies appropriate?** | **Was critical appraisal conducted by two or more reviewers independently?** | **Were there methods to minimize errors in data extraction?** | **Were the methods used to combine studies appropriate?** | **Was the likelihood of publication bias assessed?** | **Were recommendations for policy and/or practice supported by the reported data?** | **Were the specific directives for new research appropriate?** | **Overall appraisal** |
| --- | --- | --- | --- | --- | --- | --- | --- | --- | --- | --- | --- | --- |
| Cornely *et al.*, 2014(41) | Yes | Yes | Yes | Yes | Yes | Yes | Yes | Yes | NR | NA | Yes | Low risk of bias |
| Liao *et al.*, 2021(44) | Yes | Yes | Yes | Yes | NR | NR | NR | Yes | No | Yes | NR | High risk of bias |
| Liao *et al.*, 2022(59) | Yes | Yes | Yes | Yes | Yes | Yes | Yes | Yes | Yes | Yes | Yes | Low risk of bias |
| Okumura *et al.*, 2020(61) | Yes | Yes | Yes | Yes | NR | Yes | Yes | Yes | NR | NA | No | Low risk of bias |
| Rokkas *et al.*, 2019(62) | Yes | Yes | Yes | Yes | Yes | Yes | Yes | Yes | Yes | NA | Yes | Low risk of bias |
| Sridharan *et al.*, 2018(63) | Yes | Yes | Yes | Yes | Yes | Yes | Yes | Yes | Yes | No | Yes | Low risk of bias |
| Tashiro *et al.*, 2022(60) | Yes | Yes | Yes | Yes | Yes | Yes | Yes | Yes | Yes | NA | NA | Low risk of bias |

NA: not applicable, NR: not reported.

Table 9. JBI critical appraisal results for epidemiological studies

For Supplementary references see at end of Supplementary Table 2.

|  | **Was the sample frame appropriate to address the target population?** | **Were study participants recruited in an appropriate way?** | **Was the sample size adequate?** | **Were the study subjects and setting described in detail?** | **Was data analysis conducted with sufficient coverage of the identified sample?** | **Were valid methods used for the identification of the condition?** | **Was the condition measured in a standard, reliable way for all participants?** | **Was there appropriate statistical analysis?** | **Was the response rate adequate, and if not, was the low response rate managed appropriately?** | **Overall appraisal** |
| --- | --- | --- | --- | --- | --- | --- | --- | --- | --- | --- |
| Beran *et al.*, 2017(65) | Yes | Yes | Yes | Yes | Yes | Yes | Yes | Yes | Yes | Low risk of bias |
| Dai *et al.*, 2022(58) | Yes | Yes | Yes | Yes | Yes | Yes | Yes | Yes | NA | Low risk of bias |
| Freeman *et al.*, 2020(66) | Yes | Yes | Yes | Yes | Yes | Yes | Yes | Yes | Yes | Low risk of bias |
| Imwattana *et al.*, 2021 (Suppl 5) | Yes | Yes | Yes | Yes | Yes | Yes | Yes | Yes | Yes | Low risk of bias |
| Khun *et al.*, 2022 (68) | Yes | Yes | Yes | Yes | Yes | Yes | Yes | Yes | Yes | Low risk of bias |
| Knight *et al.*, 2015 (69) | Yes | Yes | Yes | Yes | Yes | Yes | Yes | Yes | NA | Low risk of bias |
| Putsathit *et al.*, 2021 (70) | Yes | Yes | Yes | Yes | Yes | Yes | Yes | Yes | NA | Low risk of bias |
| Thorpe *et al.*, 2019 (71) | Yes | Yes | Yes | Yes | Yes | Yes | Yes | Yes | NA | Low risk of bias |

NA: not applicable

Table 10. Studies excluded at full-text screening stage (N=431)

| **Study author** | **Year** | **Study title** | **Reason for exclusion** |
| --- | --- | --- | --- |
| S. L. Johnson, T. J. Gerding, D. N. Cornely, O. A. Chasan-Taber, S. Fitts, D. Gelone, S. P. Broom, C. Davidson, D. M. | 2014 | Vancomycin, metronidazole, or tolevamer for clostridium difficile infection: Results from two multinational, randomized, controlled trials | Comparator |
| G. C. Gimignani, A. Clementi, C. | 2022 | Clostridium difficile infection: infection of the multipathological elderly | Intervention |
| S. S. Muneer, M. Lobo, G. Brandt, D. Blatt, S. P. | 2020 | Prophylactic Vancomycin Therapy in Long Term Care Patients Colonized with Clostridioides difficile to Reduce Hospital-Acquired C. diff Infection | Intervention |
| A. G. Zamarripa, S. D. Ling, J. Amundsen, T. Thosani, N. Guha, S. Badillo, R. Kannadath, B. S. Fallon, M. B. | 2020 | Changes in the Burden of Clostridium difficile Infections (CDI) in the United States: Results from the National Inpatient Sample Database 2012-2017 | Intervention |
| T. A. U. Scott, S. Boules, M. Teigland, C. Parente, A. Nelson, W. | 2020 | CLINICAL BURDEN OF RECURRENT CLOSTRIDIOIDES DIFFICILE INFECTION IN THE MEDICARE POPULATION | Intervention |
| M. D. S. Zilberberg, A. F. Jesdale, W. M. Tjia, J. Lapane, K. | 2017 | Recurrent Clostridium difficile infection among Medicare patients in nursing homes | Intervention |
| C. Z. Lubbert, L. Borchert, J. Horner, B. Mutters, R. Rodloff, A. C. | 2016 | Epidemiology and Recurrence Rates of Clostridium difficile Infections in Germany: A Secondary Data Analysis | Intervention |
| K. L. Weiss, T. Miller, M. A. Mullane, K. Crook, D. W. Gorbach, S. L. | 2015 | Effects of proton pump inhibitors and histamine-2 receptor antagonists on response to fidaxomicin or vancomycin in patients with Clostridium difficile-Associated diarrhea | Intervention |
| C. E. A. Collins, M. D. Flahive, J. M. Emhoff, T. A. Anderson Jr, F. A. Santry, H. P. | 2014 | Epidemiology and outcomes of community-acquired clostridium difficile infections in medicare beneficiaries | Intervention |
| J. R. S. Zahar, C. Adrie, C. Garrouste-Orgeas, M. Francais, A. Vesin, A. Nguile-Makao, M. Tabah, A. Laupland, K. Le-Monnier, A. Timsit, J. F. | 2012 | Outcome of ICU patients with Clostridium difficile infection | Intervention |
| S. G. Armbruster, L. | 2012 | A 5-year retrospective review of experience with Clostridium difficile-associated diarrhea | Intervention |
| J. C. L. Lee, C. C. Chiu, C. W. Tsai, P. J. Hsueh, P. R. Lee, Y. T. Hung, Y. P. Ko, W. C. | 2022 | Reappraisal of the clinical role of metronidazole therapy for Clostridioides difficile infection in Taiwan: A multicenter prospective study | Intervention |
| T. D. Wu, S. L. Church, B. Alangaden, G. J. Kenney, R. M. | 2022 | Outcomes of clinical decision support for outpatient management of Clostridioides difficile infection | Intervention |
| J. E. C. Baek, I. H. Cho, Y. W. Cho, Y. S. | 2022 | CLINICAL CHARACTERISTICS AND OUTCOMES OF CLOSTRIDIOIDES DIFFICILE INFECTION IN THE INTENSIVE CARE UNIT | Intervention |
| J. H. L. Lee, S. J. Shin, J. Park, S. H. Cha, B. Kwon, K. S. Shin, Y. W. | 2022 | RISK FACTORS FOR TREATMENT FAILURE AFTER FECAL MICROBIOTA TRANSPLANTATION IN CLOSTRIDIOIDES DIFFICILE INFECTION | Intervention |
| S. M. D. A. Baunwall, S. E. Hansen, M. M. Kelsen, J. Hoyer, K. L. Ragard, N. Eriksen, L. L. Stoy, S. Rubak, T. Damsgaard, E. M. S. Mikkelsen, S. Erikstrup, C. Dahlerup, J. F. Hvas, C. L. | 2022 | Faecal microbiota transplantation for first or second Clostridioides difficile infection (EarlyFMT): a randomised, double-blind, placebo-controlled trial | Intervention |
| M. E. Mironova, A. C. Grinspan, A. Protano, M. A. | 2022 | Fecal microbiota transplantation may reduce the mortality of patients with severe and fulminant Clostridioides difficile infection compared to standard-of-care antibiotics in a community hospital | Intervention |
| M. F. S. Spagnol, D. Comerlato, P. H. Tonietto, T. A. Caierao, J. Pasqualotto, A. C. Lima-Morales, D. D. Martins, A. F. Falci, D. R. | 2022 | High rate of Clostridioides difficile colonization in patients admitted to intensive care: A prospective cohort study | Intervention |
| B. M. Angelica, R. Tucker, E. C. Bryant, R. V. Costello, S. | 2022 | Outcomes of fecal microbiota transplantation for Clostridioides difficile infection in South Australia | Intervention |
| S. O. Dogra, C. Sherman, A. Varughese, R. Yuen, A. Sherman, I. Cohen, A. Luo, Y. Chen, L. A. | 2022 | Long-Term Efficacy and Safety of Fecal Microbiota Transplantation for C. difficile Infections Across Academic and Private Clinical Settings | Intervention |
| K. L. Suchman, Y. Grinspan, A. | 2022 | Fecal Microbiota Transplant for Clostridioides Difficile Infection Is Safe and Efficacious in an Immunocompromised Cohort | Intervention |
| J. D. F. Y. Alves, A. de Mendonca, J. S. de Melo Gamba, C. Fonseca, C. L. Paraskevopoulos, D. K. S. de Paula, A. I. Hosino, N. Costa, S. F. Guimaraes, T. | 2022 | Metronidazole for Treatment of Clostridioides difficile Infections in Brazil: A Single-Center Experience and Risk Factors for Mortality | Intervention |
| R. V. Stebel, L. Svacinka, R. Husa, P. | 2020 | Faecal microbiota transplantation in the treatment of Clostridioides difficile infection | Intervention |
| B. P. F. Vaughn, M. Kelly, C. R. Allegretti, J. R. Graiziger, C. Thomas, J. McClure, E. Kabage, A. J. Khoruts, A. | 2022 | Effectiveness and safety of colonic and capsule fecal microbiota transplantation for recurrent Clostridioides difficile infection | Intervention |
| D. Y. M. Yang, T. Sun, H. Russell, L. Roach, B. Wong, K. Zhang, W. Kao, D. H. | 2021 | ECONOMIC EVALUATION OF A FECAL MICROBIOTA TRANSPLANTATION PROGRAM FOR RECURRENT CLOSTRIDIOIDES DIFFICILE INFECTION IN ALBERTA | Intervention |
| R. B. Najjar-Debbiny, A. Schwartz, N. Shaked, P. Saliba, W. Weber, G. | 2022 | Non-inferiority of metronidazole to vancomycin in the treatment of first episode non-severe Clostridioides difficile infection: a single center retrospective cohort study | Intervention |
| X. X. Li, F. Li, Y. Hu, H. Xiao, Y. Xu, Q. Li, D. Yu, G. Wang, Y. Zhang, T. | 2022 | Characteristics and management of children with Clostridiodes difficile infection at a tertiary pediatric hospital in China | Intervention |
| E. F. K. Yen, C. R. Chiou, I. Amusin, D. B. Serra, S. M. Nersesova, Y. Fredell, L. Laine, L. | 2022 | PRE-FMT ANTIBIOTIC USE IS ASSOCIATED WITH HIGHER RATES OF CURE AFTER FECAL MICROBIOTA TRANSPLANTATION FOR RECURRENT CLOSTRIDIOIDES DIFFICILE INFECTION | Intervention |
| Y. K. L. Yau, L. H. S. Mak, J. W. Lui, R. N. Ng, W. Y. R. Li, A. Chin, M. L. Guo, C. L. Chan, P. K. Chan, F. K. Ng, S. C. | 2022 | LONG-TERM SAFETY AND EFFICACY OF FECAL MICROBIOTA TRANSPLANTATION IN THE REAL WORLD: UP TO 8-YEAR DATA FROM THE FECAL MICROBIOTA REGISTRY IN HONG KONG | Intervention |
| V. M. C. Khaykin, A. Turgeon, D. K. Rao, K. Kao, J. Y. | 2022 | THE PREVALENCE OF NEW ONSET GI SYMPTOMS AND CLOSTRIDIUM DIFFICILE RECURRENCE POST-TRANSPLANT IN PATIENTS UNDERGOING FECAL MICROBIOTA TRANSPLANT FOR TREATMENT OF RECURRENT CLOSTRIDIUM DIFFICILE INFECTION | Intervention |
| J. L. Yan, J. Lv, T. Gu, S. Jiang, T. Huang, L. Shen, P. Fang, Y. Chen, Y. | 2017 | Epidemiology of Clostridium difficile in a county level hospital in China | Intervention |
| D. N. Popa, B. Mihalache, M. Boicean, A. Banciu, A. Banciu, D. D. Moga, D. F. C. Birlutiu, V. | 2021 | Fecal microbiota transplant in severe and non-severe clostridioides difficile infection. Is there a role of fmt in primary severe cdi? | Intervention |
| M. R. A. Nicholson, E. Bartlett, M. Becker, P. Davidovics, Z. Knackstedt, E. E. Docktor, M. Dole, M. Felix, G. Gisser, J. Hourigan, S. Jensen, K. Kaplan, J. Kelsen, J. Kennedy, M. Khanna, S. Leier, M. Lewis, J. Lodarek, A. Michail, S. Mitchell, P. Oliva-Hemker, M. Patton, T. Queliza, K. Singh, N. Solomon, A. Suskind, D. Werlin, S. Kellermayer, R. Kahn, S. | 2018 | A multicenter study of fecal microbiota transplantation for Clostridium difficile infection in children | Intervention |
| A. T. Amin, C. Mohammadi, I. Murunga, A. Schablik, J. Guo, A. | 2022 | Contemporary unmet needs and high mortality in recurring clostridium difficile patients | Intervention |
| H. R. Gadhikar, H. Patwardhan, S. Gandhi, A. Parikshit, P. Bapaye, A. Bhagwat, S. | 2022 | To study the clinical profile, risk factors and treatment outcomes of Clostridoitis difficile associated diarrhea. An observational retrospective study in a tertiary hospital from western India | Intervention |
| E. W. Aguilar-Zamora, B. C. Torres, R. C. Gomez-Delgado, A. Ortiz-Olvera, N. Aparicio-Ozores, G. Barbero-Becerra, V. J. Torres, J. Camorlinga-Ponce, M. | 2022 | Molecular Epidemiology and Antimicrobial Resistance of Clostridioides difficile in Hospitalized Patients From Mexico | Intervention |
| C. D. Nivet, V. Beaurain, M. Delobel, P. Quelven, I. Alric, L. | 2022 | Fecal Microbiota Transplantation for Refractory Clostridioides Difficile Infection Is Effective and Well Tolerated Even in Very Old Subjects: A Real-Life Study | Intervention |
| E. P. D. Josep, B. M. M. Sergio, G. M. Julia, B. A. Esther, V. E. Raul, F. P. | 2021 | Compounding, stability evaluation, efficacy and safety of vancomycin syrup for the treatment of clostridioides difficile infection | Intervention |
| M. B. Klezovich-Benard, F. Rouveix, E. Goossens, P. L. Davido, B. | 2021 | Management and characteristics of patients suffering from Clostridiodes difficile infection in primary care | Intervention |
| F. S. Cold, C. K. Petersen, A. M. Hansen, L. H. Helms, M. | 2022 | Long-Term Safety Following Faecal Microbiota Transplantation as a Treatment for Recurrent Clostridioides difficile Infection Compared with Patients Treated with a Fixed Bacterial Mixture: Results from a Retrospective Cohort Study | Intervention |
| M. K. Sholeh, E. Talebi, M. Hallajzadeh, M. Godarzi, F. Amirmozafari, N. | 2021 | Toxin gene profiles and antimicrobial resistance of Clostridioides difficile infection: a single tertiary care center study in Iran | Intervention |
| M. v. D. Jovanovic, S. M. Drakulovic, M. Papic, D. Pavic, S. Jovanovic, S. Lesic, A. Korac, M. Milosevic, I. Kuijper, E. J. | 2020 | A pilot study in Serbia by European clostridium difficile infection surveillance network | Intervention |
| S. V. Vigvari, A. Solt, J. Sipos, D. Feiszt, Z. Kovacs, B. Kappeter, A. Peterfi, Z. | 2018 | Experiences with fecal microbiota transplantation in Clostridium difficile infections via upper gastrointestinal tract | Intervention |
| G. B. Ianiro, S. Porcari, S. Settanni, C. R. Giambo, F. Curta, A. R. Quaranta, G. Scaldaferri, F. Masucci, L. Sanguinetti, M. Gasbarrini, A. Cammarota, G. | 2021 | Fecal microbiota transplantation for recurrent C. difficile infection in patients with inflammatory bowel disease: experience of a large-volume European FMT center | Intervention |
| K. I. Misawa, O. Enoki, Y. Taguchi, K. Uno, S. Uwamino, Y. Hasegawa, N. Matsumoto, K. | 2021 | Retrospective study of the efficacy and safety of metronidazole and vancomycin for Clostridioides difficile infection | Intervention |
| E. S. d. M. T. Girao, B. dos Santos, S. A. Gamarra, G. L. Rizek, C. Martins, R. C. Neto, L. V. P. Diogo, C. D' Annibale Orsi T Morales, H. M. P. da Silva Nogueira, K. Maestri, A. C. Boszczowski, I. Piastrelli, F. Costa, C. L. Costa, D. V. Maciel, G. Romao, J. Guimaraes, T. de Castro Brito, G. A. Costa, S. F. | 2021 | Predictive factors, outcomes, and molecular epidemiology of Clostridioides difficile diarrhea in Brazilian hospitals | Intervention |
| A. J. O. Gonzales-Luna, A. O. Shen, W. J. Deshpande, A. Carlson, T. J. Dotson, K. M. Lancaster, C. Begum, K. Alam, M. J. Hurdle, J. G. Garey, K. W. | 2021 | Reduced Susceptibility to Metronidazole Is Associated with Initial Clinical Failure in Clostridioides difficile Infection | Intervention |
| A. H. G. Rupawala, D. Bakhit, M. Jimoh, L. Kelly, C. R. | 2021 | Management of Severe and Severe/Complicated Clostridoides difficile Infection Using Sequential Fecal Microbiota Transplant by Retention Enema | Intervention |
| H. K. Ali, S. Ma, W. Peng, Y. Jiang, Z. D. DuPont, H. Zhang, H. C. Thomas, A. S. Okhuysen, P. Wang, Y. | 2021 | Safety and efficacy of fecal microbiota transplantation to treat and prevent recurrent Clostridioides difficile in cancer patients | Intervention |
| C. A. C. Gentry, D. L. Williams, R. J. | 2021 | Outcomes associated with recent guideline recommendations removing metronidazole for treatment of non-severe Clostridioides difficile infection: a retrospective, observational, nationwide cohort study | Intervention |
| R. S. Tariq, S. Solanky, D. Pardi, D. S. Khanna, S. | 2021 | Predictors and Management of Failed Fecal Microbiota Transplantation for Recurrent Clostridioides difficile Infection | Intervention |
| L. A. Shokoohizadeh, F. Yadegar, A. Azimirad, M. Hashemi, S. H. Alikhani, M. Y. | 2021 | Frequency of toxin genes and antibiotic resistance pattern of Clostridioides difficile isolates in diarrheal samples among hospitalized patients in Hamadan, Iran | Intervention |
| A. M. Agarwal, A. Verma, S. Arrup, D. Phillips, L. Vinayek, R. Nair, P. Hagan, M. Dutta, S. | 2021 | Superiority of Higher-Volume Fresh Feces Compared to Lower-Volume Frozen Feces in Fecal Microbiota Transplantation for Recurrent Clostridioides Difficile Colitis | Intervention |
| C. P. Haifer, S. Borody, T. J. Clancy, A. Leong, R. W. Kaakoush, N. O. | 2021 | Long-term bacterial and fungal dynamics following oral lyophilized fecal microbiota transplantation in clostridioides difficile infection | Intervention |
| B. J. A. Ereshefsky, D. El Nekidy, W. S. Pontiggia, L. Ghazi, I. M. | 2021 | Optimal vancomycin dose in the treatment of Clostridium difficile infection, antimicrobial stewardship initiative | Intervention |
| J. N. N. O'Donnell, G. M. Bratek, B. R. Singh, G. Duru, O. O. Mitchell, C. L. Roddy, K. M. Bidell, M. R. | 2021 | Effect of oral vancomycin dose on outcomes in patients with Clostridioides difficile infection | Intervention |
| J. R. M. Allegretti, S. R. Kassam, Z. Kelly, C. R. Kao, D. Xu, H. Fischer, M. | 2021 | Risk Factors that Predict the Failure of Multiple Fecal Microbiota Transplantations for Clostridioides difficile Infection | Intervention |
| C. R. Y. Kelly, E. F. Grinspan, A. M. Kahn, S. A. Atreja, A. Lewis, J. D. Moore, T. A. Rubin, D. T. Kim, A. M. Serra, S. Nersesova, Y. Fredell, L. Hunsicker, D. McDonald, D. Knight, R. Allegretti, J. R. Pekow, J. Absah, I. Hsu, R. Vincent, J. Khanna, S. Tangen, L. Crawford, C. V. Mattar, M. C. Chen, L. A. Fischer, M. Arsenescu, R. I. Feuerstadt, P. Goldstein, J. Kerman, D. Ehrlich, A. C. Wu, G. D. Laine, L. | 2021 | Fecal Microbiota Transplantation Is Highly Effective in Real-World Practice: Initial Results From the FMT National Registry | Intervention |
| S. M. Saha, K. Pardi, D. S. Khanna, S. | 2021 | Durability of Response to Fecal Microbiota Transplantation After Exposure to Risk Factors for Recurrence in Patients With Clostridioides difficile Infection | Intervention |
| S. Z. Gupta, J. McCarty, T. R. Pruce, J. Kassam, Z. Kelly, C. Fischer, M. Allegretti, J. R. | 2021 | Cost-effectiveness analysis of sequential fecal microbiota transplantation for fulminant Clostridioides difficile infection | Intervention |
| C. V. Bestfater, M. J. G. T. Stallmach, A. Tuffers, K. Erhardt, A. Frank, T. Gluck, T. Goeser, F. Sellge, G. Solbach, P. Eisenlohr, H. Storr, M. | 2021 | Clinical effectiveness of bidirectional fecal microbiota transfer in the treatment of recurrent Clostridioides difficile infections | Intervention |
| K. Rosenberg | 2021 | Fecal Microbiota Transplantation is Safe and Effective for C. Difficile Infection | Intervention |
| J. P. El Halabi, N. P. Fox, K. Kohane, I. Farhat, M. R. | 2021 | Fecal microbiota transplantation and clostridioides difficile infection among privately insured patients in the United States | Intervention |
| N. T. Vobugari, O. Nadella, S. Ramirez, M. P. Kilian, N. Mattar, M. C. | 2021 | Characterization of factors and outcomes associated with recurrent clostridium difficile infection in patients after fecal microbiota transplant | Intervention |
| J. C. Damianos, A. Rizwan, R. Mansoor, M. S. Sharma, P. Ejaz Malik, U. Alnahhas, H. Feuerstadt, P. | 2021 | Concomitant cephalosporins or vancomycin during or after treatment of clostridioides difficile infection increases risk of 3-month CDI recurrence | Intervention |
| M. F. T. Ashraf, O. Nassar, Y. Batool, A. | 2021 | Fecal microbiota transplantation in patients with recurrent clostridium difficile infection: A four year single-center retrospective chart review | Intervention |
| S. W. Taylor, J. Chand, S. | 2021 | Complications and mortality in patients who have undergone fecal microbiota transplantation for Clostridium difficile infection: A single-center retrospective cohort study | Intervention |
| N. V. Idrees, C. Kurup, A. | 2021 | Single centre experience of efficacy and safety of faecal microbiota transplantation for clostridium difficile diarrhoea | Intervention |
| L. B. Hickson, D. McClements, D. | 2021 | The role of FMT in reducing hospital admissions and length of stay | Intervention |
| G. L. Miyasato, H. Katariya, D. Francis, K. Hadker, N. | 2020 | PIN77 ASSESSING THE REAL-WORLD INPATIENT TREATMENT PARADIGM FOR RECURRENT CLOSTRIDIUM DIFFICILE | Intervention |
| S. B. Haubitz, N. Bucheli Laffer, E. Spelters, C. Fankhauser, H. Fux, C. A. | 2020 | Outcome of Clostridioides difficile infections treated in a Swiss tertiary care hospital: an observational study | Intervention |
| E. M. V. Terveer, K. E. W. Ooijevaar, R. E. Lingen, E. V. Boeije-Koppenol, E. Nood, E. V. Goorhuis, A. Bauer, M. P. van Beurden, Y. H. Dijkgraaf, M. G. W. Mulder, C. J. J. Vandenbroucke-Grauls, C. M. J. E. Seegers, J. F. M. L. van Prehn, J. Verspaget, H. W. Kuijper, E. J. Keller, J. J. | 2020 | Faecal microbiota transplantation for Clostridioides difficile infection: Four years' experience of the Netherlands Donor Feces Bank | Intervention |
| S. W. B. Johnson, S. V. Priest, D. H. | 2020 | Effectiveness of oral vancomycin for prevention of healthcare facility-onset clostridioides difficile infection in targeted patients during systemic antibiotic exposure | Intervention |
| G. B. Ianiro, S. Masucci, L. Quaranta, G. Porcari, S. Settanni, C. R. Lopetuso, L. R. Fantoni, M. Sanguinetti, M. Gasbarrini, A. Cammarota, G. | 2020 | Maintaining standard volumes, efficacy and safety, of fecal microbiota transplantation for C. difficile infection during the COVID-19 pandemic: A prospective cohort study | Intervention |
| Y. S. Wang, A. Li, J. Gomez-Simmonds, A. Salmasian, H. Freedberg, D. E. | 2020 | Does Addition of Intravenous Metronidazole to Oral Vancomycin Improve Outcomes in Clostridioides difficile Infection? | Intervention |
| J. W. Choucair, R. Haddad, E. Chedid, M. Chehata, N. Saliba, G. Dahboul, H. | 2020 | Clostridioides difficile infections: Epidemiology, correlations and treatment in a Lebanese cohort with use of ATLAS scoring | Intervention |
| B. D. P. Navalkele, J. Sandhu, A. Awali, R. Krishna, A. Chandramohan, S. Tillotson, G. Chopra, T. | 2020 | Clinical outcomes after faecal microbiota transplant by retention enema in both immunocompetent and immunocompromised patients with recurrent Clostridioides difficile infections at an academic medical centre | Intervention |
| A. M. Baghani, A. Kuijper, E. J. Aliramezani, A. Talebi, M. Douraghi, M. | 2020 | High prevalence of Clostridiodes diffiicle PCR ribotypes 001 and 126 in Iran | Intervention |
| S. R. W. Shaffer, J. Targownik, L. E. Kao, D. Lee, C. Smieliauskas, F. Rubin, D. T. Singh, H. Bernstein, C. N. | 2020 | Cost-effectiveness analysis of a fecal microbiota transplant center for treating recurrent C.difficile infection | Intervention |
| Y. T. Luo, E. N. Grinspan, A. M. | 2020 | Fecal Microbiota Transplantation for Clostridioides difficile in High-Risk Older Adults Is Associated with Early Recurrence | Intervention |
| K. P. Imwattana, P. Leepattarakit, T. Kiratisin, P. Riley, T. V. | 2020 | Mild or malign: Clinical characteristics and outcomes of clostridium difficile infection in Thailand | Intervention |
| J. R. K. Allegretti, C. R. Grinspan, A. Mullish, B. H. Kassam, Z. Fischer, M. | 2020 | Outcomes of Fecal Microbiota Transplantation in Patients With Inflammatory Bowel Diseases and Recurrent Clostridioides difficile Infection | Intervention |
| E. M. S. Knight, D. S. Fulman, M. K. Rastogi, R. | 2020 | Long-Term Efficacy of Oral Vancomycin Prophylaxis for the Prevention of Clostridium difficile Recurrence | Intervention |
| R. D. Tariq, M. B. Baise John, K. D. Orenstein, R. Saha, S. Solanky, D. Loftus, E. V. Pardi, D. S. Khanna, S. | 2020 | Efficacy of fecal microbiota transplantation for recurrent c difficile infection in inflammatory bowel disease | Intervention |
| B. K. C. Perler, B. Phelps, E. Allegretti, J. R. Fischer, M. Ganapini, V. Krajiceck, E. Kumar, V. Marcus, J. Nativ, L. Kelly, C. R. | 2020 | Long-term efficacy and safety of fecal microbiota transplantation for treatment of recurrent clostridioides difficile infection | Intervention |
| B. F. Barberio, S. Mele, E. D'Inca, R. Sturniolo, G. C. Farinati, F. Zingone, F. Quagliariello, A. Ghisa, M. Massimi, D. Casadei, C. Savarino, E. V. | 2020 | Faecal microbiota transplantation in Clostridioides difficile infection: real-life experience from an academic Italian hospital | Intervention |
| M. Y. Azimirad, A. Gholami, F. Shahrokh, S. Aghdaei, H. A. Ianiro, G. Suzuki, H. Cammarota, G. Zali, M. R. | 2020 | Treatment of recurrent clostridioides difficile infection using fecal microbiota transplantation in iranian patients with underlying inflammatory bowel disease | Intervention |
| J. H. S. J. You, X. Lee, W. H. Chan, P. K. S. Ng, S. C. | 2020 | Cost-effectiveness analysis of fecal microbiota transplantation for recurrent Clostridium difficile infection in patients with inflammatory bowel disease | Intervention |
| F. K. M. T. F. Tilkorn, H. Simon, I. S. Schwanbeck, J. Horn, S. Zimmermann, O. Gross, U. Bohne, W. Zautner, A. E. | 2020 | Antimicrobial resistance patterns in clostridioides difficile strains isolated from neonates in Germany | Intervention |
| G. Z. P. Saldanha, R. N. Rauber, A. P. de Lima-Morales, D. Falci, D. R. Caierao, J. Pasqualotto, A. C. Martins, A. F. | 2020 | Genetic relatedness, Virulence factors and Antimicrobial Resistance of C. difficile strains from hospitalized patients in a multicentric study in Brazil | Intervention |
| K. A. C. Connor, K. M. | 2020 | Analysis of the impact of secondary prophylaxis on Clostridioides difficile recurrence in critically ill adults | Intervention |
| T. S. Kimura, S. Sugitani, T. | 2020 | Clostridioides (Clostridium) difficile infection in Japanese hospitals 2008-2017: A real-world nationwide analysis of treatment pattern, incidence and testing density | Intervention |
| J. R. M. Allegretti, J. Storm, M. Sitko, J. Kennedy, K. Gerber, G. K. Bry, L. | 2020 | Clinical Predictors of Recurrence After Primary Clostridioides difficile Infection: A Prospective Cohort Study | Intervention |
| S. M. Ishii, Y. Kusama, Y. Yagi, T. Goto, R. Ebisui, A. Kawabe, A. Inose, R. Ohmagari, N. | 2020 | The trend for antibiotic use for clostridioides (Clostridium) difficile infection in Japan | Intervention |
| V. L. Q. McCune, M. N. Manzoor, S. Moran, C. E. Banavathi, K. Steed, H. Massey, D. C. O. Trafford, G. R. Iqbal, T. H. Hawkey, P. M. | 2020 | Results from the first English stool bank using faecal microbiota transplant as a medicinal product for the treatment of Clostridioides difficile infection | Intervention |
| Y. L. Luo, A. L. Grinspan, A. M. | 2020 | Fecal Transplants by Colonoscopy and Capsules Are Cost-Effective Strategies for Treating Recurrent Clostridioides difficile Infection | Intervention |
| M. R. M. Nicholson, P. D. Alexander, E. Ballal, S. Bartlett, M. Becker, P. Davidovics, Z. Docktor, M. Dole, M. Felix, G. Gisser, J. Hourigan, S. K. Jensen, M. K. Kaplan, J. L. Kelsen, J. Kennedy, M. Khanna, S. Knackstedt, E. Leier, M. Lewis, J. Lodarek, A. Michail, S. Oliva-Hemker, M. Patton, T. Queliza, K. Russell, G. H. Singh, N. Solomon, A. Suskind, D. L. Werlin, S. Kellermayer, R. Kahn, S. A. | 2020 | Efficacy of Fecal Microbiota Transplantation for Clostridium difficile Infection in Children | Intervention |
| A. I. Caupenne, P. Ingrand, I. Forestier, E. Roubaud-Baudron, C. Gavazzi, G. Paccalin, M. | 2020 | Acute Clostridioides difficile Infection in Hospitalized Persons Aged 75 and Older: 30-Day Prognosis and Risk Factors for Mortality | Intervention |
| Y. W. P. Cheng, E. Nemes, S. Rogers, N. Sagi, S. Bohm, M. El-Halabi, M. Allegretti, J. R. Kassam, Z. Xu, H. Fischer, M. | 2020 | Fecal Microbiota Transplant Decreases Mortality in Patients with Refractory Severe or Fulminant Clostridioides difficile Infection | Intervention |
| T. B. Avni, T. Ben-Zvi, H. Hijazi, R. Ayada, G. Atamna, A. Bishara, J. | 2020 | Clostridioides difficile infection in immunocompromised hospitalized patients is associated with a high recurrence rate | Intervention |
| E. S. Novakova, M. Kopilec, M. G. Novak, M. Kotlebova, N. Kuijper, E. Krutova, M. | 2020 | The emergence of Clostridium difficile ribotypes 027 and 176 with a predominance of the Clostridium difficile ribotype 001 recognized in Slovakia following the European standardized Clostridium difficile infection surveillance of 2016 | Intervention |
| M. K. Azimirad, M. Balaii, H. Kodori, M. Shahrokh, S. Azizi, O. Yadegar, A. Aghdaei, H. A. Zali, M. R. | 2020 | Coexistence of Clostridioides difficile and Staphylococcus aureus in gut of Iranian outpatients with underlying inflammatory bowel disease | Intervention |
| N. N. Boton, J. Mack, A. Eschenauer, G. Patel, T. S. Kao, J. Rao, K. | 2020 | Evaluation of Persistent Diarrhea and Recurrence Following Fecal Microbiota Transplantation for Recurrent Clostridioides difficile Infection | Intervention |
| H. D. Bao, Y. Papadopoulos, J. Siegfried, J. Merchan, C. Lighter, J. Jen, S. P. | 2020 | Oral Vancomycin as Secondary Prophylaxis Against Clostridioides difficile Infection in Pediatric Patients | Intervention |
| E. V. D.-D. J. Van Lingen, A. E. Vendrik, K. E. W. Kuijper, E. J. Terveer, E. M. Keller, J. J. | 2020 | Fecal microbiota transplantation as treatment for recurrent Clostridiodes difficile infection in patients with inflammatory Bowel disease: Experiences of the Netherlands donor feces bank | Intervention |
| S. S. Edds, R. | 2020 | Relapsing Clostridium difficile Infection: Resolution with Combined Patient Choice Fecal Microbiota Transplantation, Vancomycin Taper, and Vancomycin Suppression | Intervention |
| E. Y. Berman, B. H. Javia, A. Kozuch, P. Choudhary, C. Shivashankar, R. | 2020 | SComparing Recurrence Rates of Clostridioides difficile Infection (CDI) for Patients with Inflammatory Bowel Disease after Long vs Short-Term Oral Vancomycin for Initial Episode of CDI | Intervention |
| K. I. L. Suchman, Y. Grinspan, A. M. | 2020 | Fecal Microbiota Transplant for Clostridioides difficile Infection in an Immunocompromised Population | Intervention |
| R. J. S. Pattison, T. Murakami, T. T. | 2020 | Factors Associated with Readmission and Recurrent Diarrhea after Fecal Microbiota Transplant (FMT) in Patients with Clostridioides difficile Infections: A Retrospective Cohort Study | Intervention |
| M. I. Popescu-Hagen, A. Grecu, A. C. Tanasescu, M. Ciolan, G. Postolache, P. | 2020 | CHALLENGES IN THE MANAGEMENT OF PATIENTS WITH TB AND CLOSTRIDIUM DIFFICILE | Intervention |
| Y. P. Namn, A. Desai, D. | 2020 | IT IS NOT COST EFFECTIVE TO USE PROPHYLACTIC ORAL VANCOMYCIN TO PREVENT RECURRENT CLOSTRIDIUM DIFFICILE INFECTION IN HOSPITALIZED PATIENTS | Intervention |
| C. P. Haifer, S. Borody, T. J. Clancy, A. Kingston-Smith, H. Leong, R. W. Kaakoush, N. O. | 2020 | ORALLY ADMINISTERED LYOPHILIZED FECAL MICROBIOTA TRANSPLANTATION IN CLOSTRIDIOIDES DIFFICILE INFECTION - LONG TERM CLINICAL AND MICROBIOLOGICAL OUTCOMES | Intervention |
| A. V. Agarwal, S. Phillips, L. Arrup, D. W. Vinayek, R. Nair, P. Hagan, M. Dutta, S. Maheshwari, A. | 2020 | SUPERIOR EFFICACY OF HIGHER VOLUME FRESH FECES VS. COMMERCIALLY AVAILABLE LOWER VOLUME FROZEN FECES FOR INTESTINAL MICROBIOTA TRANSPLANTATION OF RECURRENT C. DIFFICILE COLITIS | Intervention |
| S. T. Kilakkathi, D. Woodworth, M. H. Kraft, C. Dhere, T. A. | 2020 | OUTCOMES OF INPATIENT FECAL TRANSPLANT ADMINISTRATION FOR CLOSTRIDIODES DIFFICILE INFECTION (CDI) | Intervention |
| S. Z. Gupta, J. Kassam, Z. Kelly, C. R. Fischer, M. Allegretti, J. R. | 2020 | SEQUENTIAL FECAL MICROBIOTA TRANSPLANTATION FOR FULMINANT C. DIFFICILE INFECTION IS MORE COST EFFECTIVE THAN STANDARD ANTIBIOTIC TREATMENT | Intervention |
| C. R. Y. Kelly, E. F. Grinspan, A. Kahn, S. A. Atreja, A. Lewis, J. D. Moore, T. Rubin, D. T. Kim, A. M. Serra, S. M. Nersesova, Y. Fredell, L. Hunsicker, D. McDonald, D. Knight, R. Allegretti, J. R. Pekow, J. R. Absah, I. Hsu, R. Vincent, J. Khanna, S. Tangen, L. Crawford, C. V. Mattar, M. Chen, L. A. Fischer, M. Arsenescu, R. Wu, G. D. Laine, L. | 2020 | 37 FECAL MICROBIOTA TRANSPLANATION IS HIGHLY EFFECTIVE IN REAL-WORLD PRACTICE: INITIAL RESULTS FROM THE AMERICAN GASTROENTEROLOGICAL ASSOCIATION FECAL MICROBIOTA TRANSPLANTATION NATIONAL REGISTRY | Intervention |
| T. O. Umemura, A. Mutoh, Y. Norizuki, C. Mizuno, T. Kozaki, K. Ikeda, Y. Ichihara, T. | 2019 | Efficacy of prolonged tapered and pulsed vancomycin regimen on recurrent Clostridioides difficile infection in the Japanese setting: A case control study | Intervention |
| J. H. C. Shin, A. S. Ann Hays, R. Kolling, G. L. Vance, S. Guerrant, R. L. Archbald-Pannone, L. Warren, C. A. | 2019 | Outcomes of a multidisciplinary clinic in evaluating recurrent Clostridioides difficile infection patients for fecal microbiota transplant: A retrospective cohort analysis | Intervention |
| P. G. Kim, A. Abdul-Baki, H. Mitre, R. Mitre, M. | 2019 | Fecal microbiota transplantation in recurrent Clostridium difficile infection: A retrospective single-center chart review | Intervention |
| R. P. G. Hirten, A. Fu, S. C. Luo, Y. Suarez-Farinas, M. Rowland, J. Contijoch, E. J. Mogno, I. Yang, N. Luong, T. Labrias, P. R. Peter, I. Cho, J. H. Sands, B. E. Colombel, J. F. Faith, J. J. Clemente, J. C. | 2019 | Microbial Engraftment and Efficacy of Fecal Microbiota Transplant for Clostridium Difficile in Patients with and without Inflammatory Bowel Disease | Intervention |
| H. L. C. Tay, A. Ng, T. M. Lye, D. C. | 2019 | Risk factors and treatment outcomes of severe Clostridioides difficile infection in Singapore | Intervention |
| A. M. A. Aldrich, T. Koehler, T. J. Olivero, R. | 2019 | Analysis of Treatment Outcomes for Recurrent Clostridium difficile Infections and Fecal Microbiota Transplantation in a Pediatric Hospital | Intervention |
| H. N. Singh, Z. Walkty, A. Yu, B. N. Lix, L. M. Targownik, L. E. Bernstein, C. N. Witt, J. | 2019 | Direct cost of health care for individuals with community associated Clostridium difficile infections: A population-based cohort study | Intervention |
| R. A. Peri, R. C. Tuffers, K. Erhardt, A. Link, A. Ehlermann, P. Angeli, W. Frank, T. Storr, M. Gluck, T. Sturm, A. Rosien, U. Tacke, F. Bachmann, O. Solbach, P. Stallmach, A. Goeser, F. Vehreschild, M. J. G. T. | 2019 | The impact of technical and clinical factors on fecal microbiota transfer outcomes for the treatment of recurrent Clostridioides difficile infections in Germany | Intervention |
| J. K. Yin, L. K. Same, R. G. Hsu, A. J. Amoah, J. Tamma, P. D. | 2019 | Oral vancomycin may be associated with earlier symptom resolution than metronidazole for hospitalized children with nonsevere clostridiodes difficile infections | Intervention |
| M. H. C. Wilcox, O. A. Guery, B. Longshaw, C. Georgopali, A. Karas, A. Kazeem, G. Palacios-Fabrega, J. A. Vehreschild, M. J. G. T. | 2019 | Microbiological Characterization and Clinical Outcomes after Extended-Pulsed Fidaxomicin Treatment for Clostridioides difficile Infection in the EXTEND Study | Intervention |
| A. A. T. Alghamdi, D. | 2019 | Fecal Microbiota Transplantation after Oral Vancomycin for Recurrent Clostridium difficile Infection | Intervention |
| E. D. Kouhsari, M. Krutova, M. Fakhre Yaseri, H. Talebi, M. Baseri, Z. Moqarabzadeh, V. Sholeh, M. Amirmozafari, N. | 2019 | The emergence of metronidazole and vancomycin reduced susceptibility in Clostridium difficile isolates in Iran | Intervention |
| J. R. F. Allegretti, M. Sagi, S. V. Bohm, M. E. Fadda, H. M. Ranmal, S. R. Budree, S. Basit, A. W. Glettig, D. L. de la Serna, E. L. Gentile, A. Gerardin, Y. Timberlake, S. Sadovsky, R. Smith, M. Kassam, Z. | 2019 | Fecal Microbiota Transplantation Capsules with Targeted Colonic Versus Gastric Delivery in Recurrent Clostridium difficile Infection: A Comparative Cohort Analysis of High and Lose Dose | Intervention |
| C. F. D. Manthey, D. Christner, M. Drolz, A. Kluge, S. Lohse, A. W. Fuhrmann, V. | 2019 | Initial therapy affects duration of diarrhoea in critically ill patients with Clostridioides difficile infection (CDI) | Intervention |
| G. M. Ianiro, R. Sciume, G. D. Impagnatiello, M. Masucci, L. Ford, A. C. Law, G. R. Tilg, H. Sanguinetti, M. Cauda, R. Gasbarrini, A. Fantoni, M. Cammarota, G. | 2019 | Incidence of bloodstream infections, length of hospital stay, and survival in patients with recurrent clostridioides difficile infection treated with fecal microbiota transplantation or antibiotics a prospective cohort study | Intervention |
| E. N. V. Tixier, E. Ungaro, R. C. Grinspan, A. M. | 2019 | Faecal microbiota transplant decreases mortality in severe and fulminant Clostridioides difficile infection in critically ill patients | Intervention |
| S. M. M. Lynch, J. Grady, J. J. Stevens, R. G. Devers, T. J. | 2019 | Fecal Microbiota Transplantation for Clostridium difficile Infection: A One-Center Experience | Intervention |
| H. J. C. Appaneal, A. R. Laplante, K. L. | 2019 | What Is the Role for Metronidazole in the Treatment of Clostridium difficile Infection? Results from a National Cohort Study of Veterans with Initial Mild Disease | Intervention |
| K. W. Imwattana, P. Riley, T. V. | 2019 | High prevalence and diversity of tcdA-negative and tcdB-positive, and non-toxigenic, Clostridium difficile in Thailand | Intervention |
| J. R. K. Allegretti, Z. Fischer, M. Kelly, C. Chan, W. W. | 2019 | Risk Factors for Gastrointestinal Symptoms Following Successful Eradication of Clostridium difficile by Fecal Microbiota Transplantation (FMT) | Intervention |
| S. E. Shah, B. Pontiggia, L. Cawley, M. | 2019 | Impact of Delayed Oral Vancomycin for Severe Clostridium difficile Infection | Intervention |
| M. R. Alimolaei, H. R. Ezatkhah, M. Shamsaddini Bafti, M. Afzali, S. | 2019 | Prevalence, characteristics and antimicrobial susceptibility patterns of Clostridioides difficile isolated from hospitals in Iran | Intervention |
| R. N. W. Lui, S. H. Lau, L. H. S. Chan, T. T. Cheung, K. C. Y. Li, A. Y. L. Chin, M. L. Tang, W. W. Y. Ching, J. Y. L. Lam, K. L. Y. Chan, P. K. S. Wu, J. C. Y. Sung, J. J. Y. Chan, F. K. L. Ng, S. C. | 2019 | Faecal microbiota transplantation for treatment of recurrent or refractory clostridioides difficile infection in Hong Kong | Intervention |
| M. S.-R. Camorlinga, M. Torres, J. Romo-Castillo, M. | 2019 | Phenotypic characterization of non-toxigenic Clostridioides difficile strains isolated from patients in Mexico | Intervention |
| D. A. M. Caroff, J. T. Zhang, Z. Rhee, C. Calderwood, M. S. Kubiak, D. W. Yokoe, D. S. Klompas, M. | 2019 | Oral vancomycin prophylaxis during systemic antibiotic exposure to prevent Clostridiodes difficile infection relapses | Intervention |
| E. H. Dehlholm-Lambertsen, B. K. Jorgensen, S. M. D. Jorgensen, C. W. Jensen, M. E. Larsen, S. Jensen, J. S. Ehlers, L. Dahlerup, J. F. Hvas, C. L. | 2019 | Cost savings following faecal microbiota transplantation for recurrent Clostridium difficile infection | Intervention |
| D. E. Hudhaiah, N. | 2019 | Prevalence and genotypes of nosocomial clostridium difficile infections in the Eastern Province of the Kingdom of Saudi Arabia: A multi-centre prospective study | Intervention |
| B. A. S. Cunha, J. Blum, S. | 2018 | Enhanced efficacy of high dose oral vancomycin therapy in Clostridium difficile diarrhea for hospitalized adults not responsive to conventional oral vancomycin therapy: Antibiotic stewardship implications | Intervention |
| S. A. Y. Greenberg, I. Cohen, N. A. Livovsky, D. M. Strahilevitz, J. Israeli, E. Melzer, E. Paz, K. Fliss-Isakov, N. Maharshak, N. | 2018 | Five years of fecal microbiota transplantation - an update of the Israeli experience | Intervention |
| X. G. Li, X. Hu, H. Xiao, Y. Li, D. Yu, G. Yu, D. Zhang, T. Wang, Y. | 2018 | Clinical efficacy and microbiome changes following fecal microbiota transplantation in children with recurrent Clostridium difficile infection | Intervention |
| M. L. Hocquart, J. C. Cassir, N. Saidani, N. Eldin, C. Kerbaj, J. Delord, M. Valles, C. Brouqui, P. Raoult, D. Million, M. | 2018 | Early fecal microbiota transplantation improves survival in severe clostridium difficile infections | Intervention |
| V. V. Leung, C. Edens, T. J. Miller, M. Manges, A. R. | 2018 | Antimicrobial Resistance Gene Acquisition and Depletion Following Fecal Microbiota Transplantation for Recurrent Clostridium difficile Infection | Intervention |
| D. N. Barnes, K. Smits, S. Sonnenburg, J. Kassam, Z. Park, K. T. | 2018 | Competitively selected donor fecal microbiota transplantation: Butyrate concentration and diversity as measures of donor quality | Intervention |
| S. D. Lee, K. Simons, G. Hepple, A. Karlsson, K. Lowman, W. Gaylard, P. C. McNamara, L. Fabian, J. | 2018 | The 'ins and outs' of faecal microbiota transplant for recurrent Clostridium difficile diarrhoea at wits donald gordon medical centre, Johannesburg, South Africa | Intervention |
| G. M. Ianiro, L. Quaranta, G. Simonelli, C. Lopetuso, L. R. Sanguinetti, M. Gasbarrini, A. Cammarota, G. | 2018 | Randomised clinical trial: faecal microbiota transplantation by colonoscopy plus vancomycin for the treatment of severe refractory Clostridium difficile infection-single versus multiple infusions | Intervention |
| Y. W. Mamo, M. H. Wang, T. Dhere, T. Kraft, C. S. | 2018 | Durability and Long-term Clinical Outcomes of Fecal Microbiota Transplant Treatment in Patients with Recurrent Clostridium difficile Infection | Intervention |
| M. L. Jiang, N. H. Ip, M. You, J. H. S. | 2018 | Cost-effectiveness analysis of ribotype-guided fecal microbiota transplantation in Chinese patients with severe Clostridium difficile infection | Intervention |
| J. R. A. Allegretti, A. S. Phelps, E. Xu, H. Fischer, M. Kassam, Z. | 2018 | Classifying Fecal Microbiota Transplantation Failure: An Observational Study Examining Timing and Characteristics of Fecal Microbiota Transplantation Failures | Intervention |
| A. P. Ponte, R. Mota, M. Silva, J. Vieira, N. Oliveira, R. Rodrigues, J. Sousa, M. Sousa, I. Carvalho, J. | 2018 | Fecal microbiota transplantation in refractory or recurrent clostridium difficile infection: A real-life experience in a non-academic center | Intervention |
| S. W. N. Lam, E. A. Fraser, T. G. Delgado, D. Chalfin, D. B. | 2018 | Cost-effectiveness of three different strategies for the treatment of first recurrent Clostridium difficile infection diagnosed in a community setting | Intervention |
| A. G. Baghani, S. Aliramezani, A. Yaseri, M. Mesdaghinia, A. Douraghi, M. | 2018 | Highly antibiotic-resistant Clostridium difficile isolates from Iranian patients | Intervention |
| A. T.-R. Martinez-Melendez, L. Morfin-Otero, R. Camacho-Ortiz, A. Villarreal-Trevino, L. Sanchez-Alanis, H. Rodriguez-Noriega, E. Baines, S. D. Flores-Trevino, S. Maldonado-Garza, H. J. Garza-Gonzalez, E. | 2018 | Circulation of Highly Drug-Resistant Clostridium difficile Ribotypes 027 and 001 in Two Tertiary-Care Hospitals in Mexico | Intervention |
| M. R. K. Seo, J. Lee, Y. Lim, D. G. Pai, H. | 2018 | Prevalence, genetic relatedness and antibiotic resistance of hospital-acquired clostridium difficile PCR ribotype 018 strains | Intervention |
| M. P. Pirson, J. E. Joubert, S. Van den Bulcke, J. Leclercq, P. Avena, L. Bilge, B. Blanquet, P. Calet, K. Byl, B. | 2018 | Evaluation of the cost and length of hospital stays related to the management of an intestinal clostridium difficile infection | Intervention |
| D. H. R. Kao, B. Silva, M. Beck, P. Rioux, K. P. Madsen, K. Goodman, K. Xu, H. Chang, H. Louie, T. | 2018 | A prospective, non-inferiority, multi-center, randomized trial comparing colonoscopy vs oral capsule delivered fecal microbiota transplantation (Fmt) for recurrent clostridium difficile infection (Rcdi) | Intervention |
| S. S. Fareed, N. Stewart, F. J. Malik, A. Laghaie, E. Khizer, S. Yan, F. Pratte, Z. Lewis, J. Immergluck, L. C. | 2018 | Applying fecal microbiota transplantation (FMT) to treat recurrent Clostridium difficile infections (rCDI) in children | Intervention |
| V. B. Birlutiu, R. M. Rusu, H. M. | 2018 | The influence of the use of metronidazole associated with vancomycin in reducing the mortality rate at 30 days in patients with Clostridium difficile infection | Intervention |
| Y. B. G. Chen, S. L. Shen, P. Lv, T. Fang, Y. H. Tang, L. L. Li, L. J. | 2018 | Molecular epidemiology and antimicrobial susceptibility of Clostridium difficile isolated from hospitals during a 4-year period in China | Intervention |
| C. M. C. Psoinos, C. E. Ayturk, M. D. Anderson, F. A. Santry, H. P. | 2018 | Post-hospitalization Treatment Regimen and Readmission for C. difficile Colitis in Medicare Beneficiaries | Intervention |
| S. S. S. Hota, V. Tomlinson, G. Salpeter, M. J. McGeer, A. Coburn, B. Guttman, D. S. Low, D. E. Poutanen, S. M. | 2017 | Oral vancomycin followed by fecal transplantation versus tapering oral vancomycin treatment for recurrent clostridium difficile infection: An open-label, randomized controlled trial | Intervention |
| L. N. Tkhawkho, O. Pastukh, N. Brodsky, D. Jackson, K. Peretz, A. | 2017 | Antimicrobial susceptibility of Clostridium difficile isolates in Israel | Intervention |
| S. E. B. Bond, C. S. Yeo, W. W. Pratt, W. A. B. Orr, M. E. Miyakis, S. | 2017 | The burden of healthcare-associated Clostridium difficile infection in a non-metropolitan setting | Intervention |
| D. E. D. Z. Brumbaugh, E. F. Pyo-Twist, A. Fidanza, S. Hughes, S. Dolan, S. A. Child, J. Dominguez, S. R. | 2018 | An Intragastric Fecal Microbiota Transplantation Program for Treatment of Recurrent Clostridium difficile in Children is Efficacious, Safe, and Inexpensive | Intervention |
| J. V. Freeman, J. Pilling, S. Morris, K. Nicholson, S. Shearman, S. Longshaw, C. Wilcox, M. H. | 2018 | The ClosER study: results from a three-year pan-European longitudinal surveillance of antibiotic resistance among prevalent Clostridium difficile ribotypes, 2011-2014 | Intervention |
| T. L. Friedman-Korn, D. M. Maharshak, N. Aviv Cohen, N. Paz, K. Bar-Gil Shitrit, A. Goldin, E. Koslowsky, B. | 2018 | Fecal Transplantation for Treatment of Clostridium Difficile Infection in Elderly and Debilitated Patients | Intervention |
| V. W. N. Stevens, R. E. Schwab-Daugherty, E. M. Khader, K. Jones, M. M. Brown, K. A. Greene, T. Croft, L. D. Neuhauser, M. Glassman, P. Bidwell, G. M. Samore, M. H. Rubin, M. A. | 2017 | Comparative effectiveness of vancomycin and metronidazole for the prevention of recurrence and death in patients with clostridium difficile infection | Intervention |
| B. D. S. Sirbu, M. M. Manzo, C. Lum, J. Gerding, D. N. Johnson, S. | 2017 | Vancomycin taper and pulse regimen with careful follow-up for patients with recurrent clostridium difficile infection | Intervention |
| M. D. Watt, A. Le Monnier, A. Tilleul, P. | 2017 | Cost-effectiveness analysis on the use of fidaxomicin and vancomycin to treat Clostridium difficile infection in France | Intervention |
| R. B. Rodrigues, G. E. Ananthakrishnan, A. N. | 2017 | A comprehensive study of costs associated with recurrent clostridium difficile infection | Intervention |
| M. H.-R. Pichenot, R. Le Guern, R. Grandbastien, B. Charlet, C. Wallet, F. Schiettecatte, S. Loeuillet, F. Guery, B. Galperine, T. | 2017 | Fidaxomicin for treatment of Clostridium difficile infection in clinical practice: a prospective cohort study in a French University Hospital | Intervention |
| A. G.-D. Camacho-Ortiz, E. M. Garcia-Mazcorro, J. F. Mendoza-Olazaran, S. Martinez-Melendez, A. Palau-Davila, L. Baines, S. D. Maldonado-Garza, H. Garza-Gonzalez, E. | 2017 | Randomized clinical trial to evaluate the effect of fecal microbiota transplant for initial clostridium difficile infection in intestinal microbiome | Intervention |
| O. S. Akgul, B. Catal, F. Yuksel, P. Caliskan, R. Karasakal, O. F. Uysal, H. K. | 2017 | Is the incidence of clostridium difficile in nosocomial diarrhoea underestimated? | Intervention |
| Z. A. Peng, A. Alrabaa, S. Sun, X. | 2017 | Antibiotic resistance and toxin production of Clostridium difficile isolates from the hospitalized patients in a large hospital in Florida | Intervention |
| G. V. Ianiro, L. Masucci, L. Pecere, S. Bibbo, S. Quaranta, G. Posteraro, B. Curro, D. Sanguinetti, M. Gasbarrini, A. Cammarota, G. | 2017 | Predictors of failure after single faecal microbiota transplantation in patients with recurrent Clostridium difficile infection: results from a 3-year, single-centre cohort study | Intervention |
| Y. H. d. G. van Beurden, P. F. van Nood, E. Nieuwdorp, M. Keller, J. J. Goorhuis, A. | 2017 | Complications, effectiveness, and long term follow-up of fecal microbiota transfer by nasoduodenal tube for treatment of recurrent Clostridium difficile infection | Intervention |
| Z. D. A. Jiang, N. J. Petrosino, J. F. Jun, G. Hanis, C. L. Shah, M. Hochman, L. Ankoma-Sey, V. DuPont, A. W. Wong, M. C. Alexander, A. Ke, S. DuPont, H. L. | 2017 | Randomised clinical trial: faecal microbiota transplantation for recurrent Clostridum difficile infection - fresh, or frozen, or lyophilised microbiota from a small pool of healthy donors delivered by colonoscopy | Intervention |
| E. G. Baro, T. Denies, F. Lannoy, D. Lenne, X. Odou, P. Guery, B. Dervaux, B. | 2017 | Cost-effectiveness analysis of five competing strategies for the management of multiple recurrent community-onset Clostridium difficile infection in France | Intervention |
| J. A. S. Barkin, D. A. Fifadara, N. Barkin, J. S. | 2017 | Clostridium difficile Infection and Patient-Specific Antimicrobial Resistance Testing Reveals a High Metronidazole Resistance Rate | Intervention |
| A. L. Lo Vecchio, L. Tagliabue, C. De Giacomo, C. Garazzino, S. Mainetti, M. Cursi, L. Borali, E. De Vita, M. V. Boccuzzi, E. Castellazzi, L. Esposito, S. Guarino, A. | 2017 | Clostridium difficile infection in children: epidemiology and risk of recurrence in a low-prevalence country | Intervention |
| A. P. Carignan, S. Martin, P. Labbe, A. C. Valiquette, L. Al-Bachari, H. Montpetit, L. P. Pepin, J. | 2016 | Efficacy of Secondary Prophylaxis with Vancomycin for Preventing Recurrent Clostridium difficile Infections | Intervention |
| M. A. Agrawal, O. C. Brandt, L. J. Kelly, C. Freeman, S. Surawicz, C. Broussard, E. Stollman, N. Giovanelli, A. Smith, B. Yen, E. Trivedi, A. Hubble, L. Kao, D. Borody, T. Finlayson, S. Ray, A. Smith, R. | 2016 | The long-term efficacy and safety of fecal microbiota transplant for recurrent, severe, and complicated clostridium difficile infection in 146 elderly individuals | Intervention |
| A. A. Waye, K. Kao, D. | 2016 | Cost averted with timely fecal microbiota transplantation in the management of recurrent clostridium difficile infection in Alberta, Canada | Intervention |
| N. A. L. Cohen, D. M. Yaakobovitch, S. Yehoyada, M. B. Ami, R. B. Adler, A. Guzner-Gur, H. Goldin, E. Santo, M. E. Halpern, Z. Paz, K. Maharshak, N. | 2016 | A retrospective comparison of fecal microbial transplantation methods for recurrent Clostridium difficile infection | Intervention |
| M. G. Girotra, S. Anand, R. Song, Y. Dutta, S. K. | 2016 | Fecal Microbiota Transplantation for Recurrent Clostridium difficile Infection in the Elderly: Long-Term Outcomes and Microbiota Changes | Intervention |
| C. R. K. Kelly, A. Staley, C. Sadowsky, M. J. Abd, M. Alani, M. Bakow, B. Curran, P. McKenney, J. Tisch, A. Reinert, S. E. MacHan, J. T. Brandt, L. J. | 2016 | Effect of fecal microbiota transplantation on recurrence in multiply recurrent clostridium difficile infection a randomized trial | Intervention |
| O. C. B. Aroniadis, L. J. Greenberg, A. Borody, T. Kelly, C. R. Mellow, M. Surawicz, C. Cagle, L. Neshatian, L. Stollman, N. Giovanelli, A. Ray, A. Smith, R. | 2016 | Long-term follow-up study of fecal microbiota transplantation for severe and/or complicated clostridium difficile infection: A multicenter experience | Intervention |
| C. A. C. Sheitoyan-Pesant, C. N. Pepin, J. Marcil-Heguy, A. Nault, V. Valiquette, L. | 2016 | Clinical and Healthcare Burden of Multiple Recurrences of Clostridium difficile Infection | Intervention |
| M. A.-U. Gonzalez-Del Vecchio, A. Marin, M. Alcala, L. Martin, A. Montilla, P. Bouza, E. | 2016 | Clinical significance of Clostridium difficile in children less than 2 years old: A case-control study | Intervention |
| G. V. Samonis, K. Z. Tansarli, G. S. Dimopoulou, D. Papadimitriou, G. Kofteridis, D. P. Maraki, S. Karanika, M. Falagas, M. E. | 2016 | Clostridium difficile in Crete, Greece: Epidemiology, microbiology and clinical disease | Intervention |
| Y. L. Huang, Y. Nie, Y. | 2016 | Clinical characteristics of Clostridium difficile-associated diarrhea among patients in a tertiary care center in China | Intervention |
| T. G. K. Gweon, J. Lim, C. H. Park, J. M. Lee, D. G. Lee, I. S. Cho, Y. S. Kim, S. W. Choi, M. G. | 2016 | Fecal Microbiota Transplantation Using Upper Gastrointestinal Tract for the Treatment of Refractory or Severe Complicated Clostridium difficile Infection in Elderly Patients in Poor Medical Condition: The First Study in an Asian Country | Intervention |
| C. H. S. Lee, T. Petrof, E. O. Smieja, M. Roscoe, D. Nematallah, A. Scott Weese, J. Collins, S. Moayyedi, P. Crowther, M. Ropeleski, M. J. Jayaratne, P. Higgins, D. Li, Y. Rau, N. V. Kim, P. T. | 2016 | Frozen vs fresh fecal microbiota transplantation and clinical resolution of diarrhea in patients with recurrent clostridium difficile infection a randomized clinical trial | Intervention |
| P. C. K. Konturek, J. Dieterich, W. Haziri, D. Wirtz, S. Glowczyk, I. Konturek, K. Neurath, M. F. Zopf, Y. | 2016 | Successful therapy of Clostridium difficile infection with fecal microbiota transplantation | Intervention |
| I. M. Youngster, J. Systrom, H. K. Sauk, J. Khalili, H. Levin, J. Kaplan, J. L. Hohmann, E. L. | 2016 | Oral, frozen fecal microbiota transplant (FMT) capsules for recurrent Clostridium difficile infection | Intervention |
| B. B. Kullin, T. Rajabally, N. Anwar, F. Vedantam, G. Reid, S. Abratt, V. | 2016 | Characterisation of Clostridium difficile strains isolated from Groote Schuur Hospital, Cape Town, South Africa | Intervention |
| C. D. W. Zellmer, T. J. Van Hoof, S. Blakney, R. Safdar, N. | 2016 | Patient Perspectives on Fecal Microbiota Transplantation for Clostridium Difficile Infection | Intervention |
| Z. L. Kurti, B. D. Mandel, M. D. Csima, Z. Golovics, P. A. Csako, B. D. Mohas, A. Gonczi, L. Gecse, K. B. Kiss, L. S. Szathmari, M. Lakatos, P. L. | 2015 | Burden of Clostridium difficile infection between 2010 and 2013: Trends and outcomes from an academic center in Eastern Europe | Intervention |
| S. N. L. Bass, S. W. Bauer, S. R. Neuner, E. A. | 2015 | Comparison of oral vancomycin capsule and solution for treatment of initial episode of severe clostridium difficile infection | Intervention |
| S. M. V. Heimann, J. J. Cornely, O. A. Wisplinghoff, H. Hallek, M. Goldbrunner, R. Bottiger, B. W. Goeser, T. Holscher, A. Baldus, S. Muller, F. Jazmati, N. Wingen, S. Franke, B. Vehreschild, M. J. G. T. | 2015 | Economic burden of Clostridium difficile associated diarrhoea: a cost-of-illness study from a German tertiary care hospital | Intervention |
| S. P. C. Costello, M. A. Vuaran, M. S. Roberts-Thomson, I. C. Andrews, J. M. | 2015 | Faecal microbiota transplant for recurrent Clostridium difficile infection using long-term frozen stool is effective: Clinical efficacy and bacterial viability data | Intervention |
| J. C. D. Lagier, M. Million, M. Parola, P. Stein, A. Brouqui, P. Raoult, D. | 2015 | Dramatic reduction in Clostridium difficile ribotype 027-associated mortality with early fecal transplantation by the nasogastric route: a preliminary report | Intervention |
| N. B. Khanafer, L. Barbut, F. Hirschel, B. Vanhems, P. | 2015 | Treatment of Clostridium difficile infection in a French university hospital | Intervention |
| B. E. S. Hirsch, N. Poeth, K. Schwartz, R. M. Epstein, M. E. Honig, G. | 2015 | Effectiveness of fecal-derived microbiota transfer using orally administered capsules for recurrent Clostridium difficile infection | Intervention |
| K. E. E. J. Rokas, J. W. Beardsley, J. R. Ohl, C. A. Luther, V. P. Williamson, J. C. | 2015 | The Addition of Intravenous Metronidazole to Oral Vancomycin is Associated with Improved Mortality in Critically Ill Patients with Clostridium difficile Infection | Intervention |
| A. K. N. Thabit, D. P. | 2015 | Impact of vancomycin faecal concentrations on clinical and microbiological outcomes in Clostridium difficile infection | Intervention |
| T. L. Scardina, L. Pacheco, S. M. Adams, W. Schreckenberger, P. Johnson, S. | 2015 | Clostridium difficile infection (CDI) severity and outcome among patients infected with the NAP1/BI/027 strain in a non-epidemic setting | Intervention |
| G. M. Cammarota, L. Ianiro, G. Bibbo, S. Dinoi, G. Costamagna, G. Sanguinetti, M. Gasbarrini, A. | 2015 | Randomised clinical trial: Faecal microbiota transplantation by colonoscopy vs. vancomycin for the treatment of recurrent Clostridium difficile infection | Intervention |
| R. U. B. Varier, E. Smith, K. J. Roberts, M. S. Kyle Jensen, M. LaFleur, J. Nelson, R. E. | 2015 | Cost-effectiveness analysis of fecal microbiota transplantation for recurrent Clostridium difficile infection | Intervention |
| I. R. Youngster, G. H. Pindar, C. Ziv-Baran, T. Sauk, J. Hohmann, E. L. | 2014 | Oral, Capsulized, Frozen Fecal Microbiota Transplantation for Relapsing Clostridium difficile Infection | Intervention |
| M. A. S. Khan, A. A. Ahmad, U. Alaradi, O. Khan, A. R. Hammad, T. Pratt, J. Sodeman, T. Sodeman, W. Kamal, S. Nawras, A. | 2014 | Efficacy and safety of, and patient satisfaction with, colonoscopic-administered fecal microbiota transplantation in relapsing and refractory community- and hospital-acquired Clostridium difficile infection | Intervention |
| H. A. D. Horton, S. Berel, D. Hirsch, J. Ippoliti, A. McGovern, D. Kaur, M. Shih, D. Dubinsky, M. Targan, S. R. Fleshner, P. Vasiliauskas, E. A. Grein, J. Murthy, R. Melmed, G. Y. | 2014 | Antibiotics for treatment of Clostridium difficile infection in hospitalized patients with inflammatory bowel disease | Intervention |
| F. C. Emanuelsson, B. E. B. Ljungstrom, L. Tvede, M. Ung, K. A. | 2014 | Faecal microbiota transplantation and bacteriotherapy for recurrent Clostridium difficile infection: A retrospective evaluation of 31 patients | Intervention |
| R. E. Pathak, H. A. Patel, A. Wickremesinghe, P. | 2014 | Treatment of relapsing clostridium difficile infection using fecal microbiota transplantation | Intervention |
| C. R. I. Kelly, C. Fischer, M. Khoruts, A. Surawicz, C. Afzali, A. Aroniadis, O. Barto, A. Borody, T. Giovanelli, A. Gordon, S. Gluck, M. Hohmann, E. L. Kao, D. Kao, J. Y. McQuillen, D. P. Mellow, M. Rank, K. M. Rao, K. Ray, A. Schwartz, M. A. Singh, N. Stollman, N. Suskind, D. L. Vindigni, S. M. Youngster, I. Brandt, L. | 2014 | Fecal microbiota transplant for treatment of clostridium difficile infection in immunocompromised patients | Intervention |
| A. S. Ray, R. Breaux, J. | 2014 | Fecal microbiota transplantation for clostridium difficile infection: The ochsner experience | Intervention |
| C. H. B. Lee, J. E. Kassam, Z. Smieja, M. Higgins, D. Broukhanski, G. Kim, P. T. | 2014 | The outcome and long-term follow-up of 94 patients with recurrent and refractory Clostridium difficile infection using single to multiple fecal microbiota transplantation via retention enema | Intervention |
| S. K. Keshavamurthy, C. G. Fraser, T. G. Gordon, S. M. Houghtaling, P. L. Soltesz, E. G. Blackstone, E. H. Pettersson, G. B. | 2014 | Clostridium difficile infection after cardiac surgery: Prevalence, morbidity, mortality, and resource utilization | Intervention |
| I. S. Youngster, J. Pindar, C. Wilson, R. G. Kaplan, J. L. Smith, M. B. Alm, E. J. Gevers, D. Russell, G. H. Hohmann, E. L. | 2014 | Fecal microbiota transplant for relapsing clostridium difficile infection using a frozen inoculum from unrelated donors: A randomized, open-label, controlled pilot study | Intervention |
| A. P. Gupta, R. Baddour, L. M. Pardi, D. S. Khanna, S. | 2014 | Extraintestinal clostridium difficile infections: A single-center experience | Intervention |
| A. C. R. Buchler, S. K. Stelling, S. Ledergerber, B. Peter, S. Schweiger, A. Ruef, C. Zbinden, R. Speck, R. F. | 2014 | Antibiotic susceptibility of Clostridium difficile is similar worldwide over two decades despite widespread use of broad-spectrum antibiotics: an analysis done at the University Hospital of Zurich | Intervention |
| V. C. Beran, D. Vobejdova, J. Konigova, A. Nemec, J. Tvrdik, J. | 2014 | Sensitivity to antibiotics of Clostridium difficile toxigenic nosocomial strains | Intervention |
| R. U. B. Varier, E. Smith, K. J. Roberts, M. S. Jensen, M. K. LaFleur, J. Nelson, R. E. | 2014 | Cost-effectiveness analysis of treatment strategies for initial Clostridium difficile infection | Intervention |
| E. V. Van Nood, A. Nieuwdorp, M. Fuentes, S. Zoetendal, E. G. De Vos, W. M. Visser, C. E. Kuijper, E. J. Bartelsman, J. F. W. M. Tijssen, J. G. P. Speelman, P. Dijkgraaf, M. G. W. Keller, J. J. | 2013 | Duodenal infusion of donor feces for recurrent clostridium difficile | Intervention |
| A. A. S. Venugopal, S. Sanchez, K. Sessions, R. Johnson, L. B. | 2013 | Assessment of 30-day all-cause mortality in metronidazole-treated patients with clostridium difficile infection | Intervention |
| S. M. U. Bartsch, C. A. Fishman, N. Lee, B. Y. | 2013 | Is fidaxomicin worth the cost? An economic analysis | Intervention |
| M. G. Goudarzi, H. Alebouyeh, M. Azimi Rad, M. Shayegan Mehr, F. S. Zali, M. R. Aslani, M. M. | 2013 | Antimicrobial susceptibility of clostridium difficile clinical isolates in Iran | Intervention |
| S. W. B. Lam, S. N. Neuner, E. A. Bauer, S. R. | 2013 | Effect of vancomycin dose on treatment outcomes in severe Clostridium difficile infection | Intervention |
| T. K. Berdichevski, N. Rahav, G. Bar-Meir, S. Eliakim, R. Ben-Horin, S. | 2013 | The impact of pseudomembrane formation on the outcome of Clostridium difficile-associated disease | Intervention |
| M. D. Ingle, A. Desai, D. Abraham, P. Joshi, A. Gupta, T. Rodrigues, C. | 2013 | Clostridium difficile as a cause of acute diarrhea: A prospective study in a tertiary care center | Intervention |
| P. K. H. Kim, H. C. Cohen, H. W. Feinberg, E. J. Ahmad, S. Coyle, C. Teperman, S. Boothe, H. | 2013 | Intracolonic vancomycin for severe Clostridium difficile colitis | Intervention |
| S. N. B. Bass, S. R. Neuner, E. A. Lam, S. W. | 2013 | Comparison of treatment outcomes with vancomycin alone versus combination therapy in severe Clostridium difficile infection | Intervention |
| E. U.-S. Mattila, R. Wuorela, M. Lehtola, L. Nurmi, H. Ristikankare, M. Moilanen, V. Salminen, K. Seppala, M. Mattila, P. S. Anttila, V. Arkkila, P. | 2012 | Fecal transplantation, through colonoscopy, is effective therapy for recurrent Clostridium difficile infection | Intervention |
| L. J. A. Brandt, O. C. Mellow, M. Kanatzar, A. Kelly, C. Park, T. Stollman, N. Rohlke, F. Surawicz, C. | 2012 | Long-term follow-up of colonoscopic fecal microbiota transplant for recurrent clostridium difficile infection | Intervention |
| F. Y. A.-K. Khan, M. Anand, D. Baager, K. Alaini, A. Siddique, M. A. Mohamed, S. F. Ali, M. I. Al Bedawi, M. M. Naser, M. S. | 2012 | Epidemiological features of Clostridium difficile infection among inpatients at Hamad General Hospital in the state of Qatar, 2006-2009 | Intervention |
| J. M. S. Wenisch, D. Kuo, H. W. Allerberger, F. Michl, V. Tesik, P. Tucek, G. Laferl, H. Wenisch, C. | 2012 | Prospective observational study comparing three different treatment regimes in patients with Clostridium difficile infection | Intervention |
| F. A. Le, V. Shah, D. N. Salazar, M. Palmer, H. R. Garey, K. W. | 2012 | A real-world evaluation of oral vancomycin for severe Clostridium difficile infection: Implications for antibiotic stewardship programs | Intervention |
| C. H. Jorup-Ronstrom, A. Sandell, S. Edvinsson, O. Midtvedt, T. Persson, A. K. Norin, E. | 2012 | Fecal transplant against relapsing Clostridium difficile-associated diarrhea in 32 patients | Intervention |
| A. A. R. Venugopal, K. Patel, S. M. Szpunar, S. Jahamy, H. Valenti, S. Shemes, S. P. Khatib, R. Johnson, L. B. | 2012 | Lack of association of outcomes with treatment duration and microbiologic susceptibility data in Clostridium difficile infections in a non-NAP1/BI/027 setting | Intervention |
| C. L. J. Hvas, S. M. D. Jorgensen, S. P. Storgaard, M. Lemming, L. Erikstrup, C. Dahlerup, J. F. | 2018 | Randomized controlled clinical trial: faecal microbiota transplantation (FMT) is superior to both fidaxomicin and vancomycin monotherapies for recurrent clostridium difficile-associated disease | Intervention |
| H. J. Dupont, Z. D. Alexander, A. Ajami, N. Petrosino, J. F. DuPont, A. W. Ke, S. Jun, G. Hanis, C. | 2017 | Lyophilized fecal microbiota transplantation capsules for recurrent clostridium difficile infection | Intervention |
| P. K. Goldeh, P. Abouanaser, S. Partlow, E. Beckett, P. Onishi, C. Smieja, M. Lee, C. | 2017 | Prospective, open-label trial to evaluate efficacy of lyophilized fecal microbiota transplantation for treatment of recurrent C. difficile infection | Intervention |
| G. M. Ianiro, L. Quaranta, G. Simonelli, C. Rizzatti, G. Lopetuso, L. Mele, M. C. Sanguinetti, M. Gasbarrini, A. Cammarota, G. | 2018 | Randomized clinical trial: single-infusion FMT versus multiple-infusion FMT for the treatment of severe C. difficile infection | Intervention |
| D. H. R. Kao, B. Silva, M. Beck, P. L. Rioux, K. Xu, H. Madsen, K. Clement, F. Kaplan, G. Chang, H. J. Goodman, K. Louie, T. | 2017 | A prospective, non-inferioity, multi-center, randomized trial comparing colonoscop vs oral capsule delivered fecal microbiota transplantation in the treatment of recurrent clostridium difficile infection | Intervention |
| G. G. Merlo, N. Connelly, L. | 2015 | Economic evaluation of fecal microbiota transplantation for the treatment of recurrent clostridium difficile infection in Australia | Intervention |
| F. E. S. Juul, H. Oines, M. N. Wiig, H. Rose, O. Seip, B. Furholm, S. Midtvedt, T. Kalager, M. Loberg, M. Garborg, K. Bretthauer, M. | 2018 | FECAL MICROBIOTA TRANSPLANT VERSUS ANTIBIOTICS FOR PRIMARY CLOSTRIDIUM DIFFICILE INFECTION - A MULTICENTER, RANDOMIZED PROOF-OF-CONCEPT TRIAL | Intervention |
| A. A. C. Rode, M. Heno, K. K. Krogsgaard, L. R. Helms, M. Engberg, J. Schoenning, K. Tvede, M. Andersen, C. O. Jensen, U. S. Petersen, A. M. Bytzer, P. | 2019 | Cure of recurrent Clostridium difficile infection with a mix of 12 gut bacteria , faecal microbiota transplantation or oral vancomycin: results from an open-label multicentre randomised controlled trial | Intervention |
| S. W. P. Johnson, D. H. Brown, S. V. | 2019 | Effectiveness of oral vancomycin for prevention of healthcare facility-onset clostridioides difficile infection in high-risk patients | Intervention |
| A. H. Brumand, L. Akbar, A. Carlson, B. | 2019 | Efficacy of fecal microbial transplant in treatment of recurrent clostridioides difficileinfection in community hospitals in Las Vegas | Intervention |
| C. B. Cicerone, G. Lamonaca, L. D'Abramo, A. Oliva, A. Zingaropoli, M. A. Vullo, V. Trancassini, M. Gagliardi, A. Totino, V. Pantanella, F. Schippa, S. Corazziari, E. S. | 2017 | Fecal microbiota transplantation via enema for recurrent clostridium difficile infection modulates the inflammatory host response and restores intestinal dysbiosis | Intervention |
| D. R. Kao, B. Hotte, N. Silva, M. Madsen, K. Beck, P. Louie, T. | 2016 | A prospective, dual center, randomized trial comparing colonoscopy versus capsule delivered fecal microbiota transplantation (FMT) in the management of recurrent clostridium difficile infection (RCDI) | Intervention |
| S. C. C. W. Ng, S. H. Lui, R. N. Cheung, K. Ching, J. Y. L. Tang, W. Kyaw, M. Tao, Z. Ho, K. T. Ip, M. Chan, P. Chan, F. K. L. Sung, J. J. Y. Wu, J. C. Lam, L. Y. K. | 2017 | Vancomycin followed by fecal microbiota transplantation versus vancomycin for initial clostridium difficile infection: an open-label randomised controlled trial | Intervention |
| J. R. F. Allegretti, M. Papa, E. Elliott, R. J. Klank, J. Mendolia, G. Vo, E. Kassam, Z. Smith, M. | 2016 | Fecal microbiota transplantation delivered via oral capsules achieves microbial engraftment similar to traditional delivery modalities: safety, efficacy and engraftment results from a multi-center cluster randomized dose-finding study | Intervention |
| D. C. Crook, O. Esposito, R. Poirier, A. Somero, M. Weiss, K. Tillotson, G. | 2012 | Clostridium difficile in 7 European countries and North America: fidaxomicin vs vancomycin therapy | Intervention |
| G. M. Ianiro, L. Valerio, L. Nagel, D. D'Aversa, F. Poto, R. Dibitetto, F. Scaldaferri, F. Gesualdo, M. Sanguinetti, M. Gasbarrini, A. Cammarota, G. | 2016 | Fecal microbiota transplantation for recurrent C. Difficile infection: analysis of factors associated with the need for multiple fecal infusions | Intervention |
| A. S. K. Gargis, Maria Paulick, Ashley L. Anderson, Karen F. Adamczyk, Michelle Vlachos, Nicholas Kent, Alyssa G. McAllister, Gillian A. McKay, Susannah L. Halpin, Alison L. Albrecht, Valerie Campbell, Davina Korhonen, Lauren Elkins, Christopher A. Rasheed, J. Kamile Guh, Alice Y. McDonald, L. Clifford Lutgring, Joseph D. Emerging Infections Program, C. difficile Infection Working Group | 2022 | Reference Susceptibility Testing and Genomic Surveillance of Clostridioides difficile, United States, 2012-17 | Intervention |
| K. B. Zhang, Patricia Abouanaser, Salaheddin Smieja, Marek | 2021 | Initial vancomycin versus metronidazole for the treatment of first-episode non-severe Clostridioides difficile infection | Intervention |
| A. H. Nowak, Magnus Ursing, Johan Lidman, Christer Nowak, Piotr | 2019 | Efficacy of Routine Fecal Microbiota Transplantation for Treatment of Recurrent Clostridium difficile Infection: A Retrospective Cohort Study | Intervention |
| C. A. G. Gentry, Stephanie E. Thind, Sharanjeet Kurdgelashvili, George Skrepnek, Grant H. Williams, Riley J., 2nd | 2017 | A Propensity-Matched Analysis Between Standard Versus Tapered Oral Vancomycin Courses for the Management of Recurrent Clostridium difficile Infection | Intervention |
| R. P. Tariq, Darrell S. Bartlett, Mark G. Khanna, Sahil | 2019 | Low Cure Rates in Controlled Trials of Fecal Microbiota Transplantation for Recurrent Clostridium difficile Infection: A Systematic Review and Meta-analysis | Intervention |
| R. O. Razik, Majdi Lieberman, Alexandra Allegretti, Jessica R. Kassam, Zain | 2017 | Faecal microbiota transplantation for Clostridium difficile infection: a multicentre study of non-responders | Intervention |
| E. N. B. Abu-Khader, Eman F. Shehabi, Asem A. | 2017 | Epidemiological Features of Clostridium difficile Colonizing the Intestine of Jordanian Infants | Intervention |
| S. F. Hagel, Anne Ehlermann, Philipp Frank, Thorsten Tueffers, Kester Sturm, Andreas Link, Alexander Demir, Muenevver Siebenhaar, Arno Storr, Martin Glueck, Thomas Siegel, Erhard Solbach, Philip Goeser, Felix Koelbel, Christian B. Lohse, Ansgar Luebbert, Christoph Kandzi, Ulrich Maier, Matthias Schuerle, Stefanie Lerch, Markus M. Tacke, Daniela Cornely, Oliver A. Stallmach, Andreas Vehreschild, Maria German Clinical Microbiome Study, Group | 2016 | Fecal Microbiota Transplant in Patients With Recurrent Clostridium Difficile Infection | Intervention |
| W. Y. R. Jamal, Vincent O. | 2016 | Surveillance of Antibiotic Resistance among Hospital- and Community-Acquired Toxigenic Clostridium difficile Isolates over 5-Year Period in Kuwait | Intervention |
| J. V. Freeman, Jonathan Vickers, Richard Wilcox, Mark H. | 2016 | Susceptibility of Clostridium difficile Isolates of Varying Antimicrobial Resistance Phenotypes to SMT19969 and 11 Comparators | Intervention |
| D. R. M. Snydman, L. A. Jacobus, N. V. Thorpe, C. Stone, S. Jenkins, S. G. Goldstein, E. J. C. Patel, R. Forbes, B. A. Mirrett, S. Johnson, S. Gerding, D. N. | 2015 | U.S.-Based National Sentinel Surveillance Study for the Epidemiology of Clostridium difficile-Associated Diarrheal Isolates and Their Susceptibility to Fidaxomicin | Intervention |
| M. N. Malamood, Eric Ehrlich, Adam C. Friedenberg, Frank K. | 2015 | Vancomycin Enemas as Adjunctive Therapy for Clostridium difficile Infection | Intervention |
| H. B. Zowall, C. Deutsch, A. | 2014 | Cost-Effectiveness of Fecal Microbiota Transplant in Treating Clostridium Difficile Infection in Canada | Intervention |
| J. G. C. Cobo Reinoso, S. Mensa Pueyo, J. Salavert Lleti, M. Toledo, A. Anguita, P. Rubio-Terres, C. Rubio-Rodriguez, D. | 2014 | Economic Evaluation of Fidaxomicin for the Treatment of Clostridium Difficile Infections (CDI) also Known as Clostridium Difficile-Associated Diarrhoea (CDAD) in Spain | Intervention |
| A. L. Van Engen, X. Noren, T. Nordling, S. Norgaard, K. | 2014 | Cost-Effectiveness of Fidaxomicin for the Treatment of Clostridium Difficile Infection (CDI) in Sweden | Intervention |
| S. C. Nordling, E. | 2014 | The Budget Impact of Using Fidaxomicin for Hospitalised Cdi Patients from the Danish Health Care Perspective | Intervention |
| P. W. Petryszyn, A. | 2014 | Cost-Utility Analysis Of Fidaxomicin Compared To Vancomycin In The Management Of Severe Clostridium Difficile Infection In Poland | Intervention |
| O. A. C. Cornely, Derrick W. Esposito, Roberto Poirier, Andre Somero, Michael S. Weiss, Karl Sears, Pamela Gorbach, Sherwood O. P. T. Clinical Study Group | 2012 | Fidaxomicin versus vancomycin for infection with Clostridium difficile in Europe, Canada, and the USA: a double-blind, non-inferiority, randomised controlled trial | Intervention |
| J. A. Z. Karlowsky, George G. Hammond, Greg W. Rubinstein, Ethan Wylie, John Du, Tim Mulvey, Michael R. Alfa, Michelle J. | 2012 | Multidrug-resistant North American pulsotype 2 Clostridium difficile was the predominant toxigenic hospital-acquired strain in the province of Manitoba, Canada, in 2006-2007 | Intervention |
| L. H. B. Hammeken, S. M. D. Dahlerup, J. F. Hvas, C. L. Ehlers, L. H. | 2022 | Health-related quality of life in patients with recurrent Clostridioides difficile infections | Other |
| C. E. H. M. Ferre-Aracil, I. Vera Mendoza, M. I. Ramos Martinez, A. Munez Rubio, E. Fernandez-Cruz, A. Matallana Royo, V. Garcia-Maseda, S. Sanchez Romero, I. Martinez Ruiz, R. Calleja Panero, J. L. | 2022 | Faecal microbiota transplantation is a simple, effective and safe treatment in the management of C. difficile infection in daily clinical practice | Other |
| C. A. G. Arajol, A. Gonzalez-Suarez, B. Casals-Pascual, C. Marti Marti, S. Dominguez Luzon, M. A. Soriano, A. Guardiola Capon, J. | 2021 | Donor selection for faecal microbiota transplantation. Consensus document of the Catalan Society of Gastroenterology and the Catalan Society of Infectious Diseases and Clinical Microbiology | Other |
| J. G. Chen, C. Hitchcock, M. M. Holubar, M. Deresinski, S. Hay, J. W. | 2020 | PIN11 COST-EFFECTIVENESS OF BEZLOTOXUMAB AND FIDAXOMICIN FOR RECURRENT C. DIFFICILE INFECTION | Other |
| Y. O. Jiang, E. N. Sears, P. S. Sarpong, E. | 2020 | PGI13 A BUDGET IMPACT ANALYSIS OF FIDAXOMICIN FOR THE MANAGEMENT OF CLOSTRIDIOIDES DIFFICILE INFECTION IN THE UNITED STATES | Other |
| A. R. Rinaldi, E. E. Jessica, S. Stevenson, K. Coe, K. E. | 2020 | Effectiveness of Fidaxomicin versus Oral Vancomycin in the Treatment of Recurrent Clostridioides difficile | Other |
| X. S. Tan, A. M. Sirbu, B. Danziger, L. H. Gerding, D. N. Johnson, S. | 2020 | Management of Patients with Multiple Clostridioides difficile Infection Recurrences using a Tapered-Pulsed Fidaxomicin Strategy | Other |
| B. M. Guery, F. Anttila, V. J. Adomakoh, N. Aguado, J. M. Bisnauthsing, K. Georgopali, A. Goldenberg, S. D. Karas, A. Kazeem, G. Longshaw, C. Palacios-Fabrega, J. A. Cornely, O. A. Vehreschild, M. J. G. T. | 2018 | Extended-pulsed fidaxomicin versus vancomycin for Clostridium difficile infection in patients 60 years and older (EXTEND): a randomised, controlled, open-label, phase 3b/4 trial | Other |
| A. R. d. S. Lopez-Sanroman, E. Cobo Reinoso, J. del Campo Moreno, R. Foruny Olcina, J. R. Garcia Fernandez, S. Garcia Garcia de Paredes, A. Aguilera Castro, L. Ferre Aracil, C. Albillos Martinez, A. | 2017 | Results of the implementation of a multidisciplinary programme of faecal microbiota transplantation by colonoscopy for the treatment of recurrent Clostridium difficile infection | Other |
| F. Sandner | 2016 | Fidaxomicin for the treatment of Clostridium difficile infections: Significantly fewer recurrences, fewer mortalities | Other |
| C. L. Lee, T. J. Weiss, K. Valiquette, L. Gerson, M. Arnott, W. Gorbach, S. L. | 2016 | Fidaxomicin versus Vancomycin in the Treatment of Clostridium difficile Infection: Canadian Outcomes | Other |
| M. C.-G. Siller-Ruiz, N. Hernandez-Egido, S. Maria-Blazquez, A. de Frutos-Serna, M. Garcia-Sanchez, J. E. | 2014 | Epidemiology of Clostridium difficile-associated disease (CDAD) in Salamanca | Other |
| M. Herrmann | 2014 | Clostridium difficile infection: More patients lastingly cured by fidaxomicin | Other |
| O. A. V. Cornely, Mjgt Adomakoh, N. Georgopali, A. Karas, A. Kazeem, G. Guery, B. | 2017 | Sub-group analyses from the extend study: a randomised, controlled, open-label, phase III/IV study comparing the efficacy of extended-pulsed fidaxomicin with standard vancomycin therapy for sustained clinical cure of clostridium difficile infection in an older population | Other |
| H. U. Okumura, M. Shoji, S. English, M. | 2019 | PIN37 COST-EFFECTIVENESS ANALYSIS OF FIDAXOMICIN FOR CLOSTRIDIUM DIFFICILE INFECTION IN JAPAN | Other |
| J. K. Wolf, K. Fortuny, C. Lazar, S. Bosis, S. Korczowski, B. Petit, A. Bradford, D. Incera, E. Melis, J. Van Maanen, R. | 2018 | Safety and efficacy of fidaxomicin and vancomycin in pediatric patients with clostridium difficile infection: phase III, multicenter, investigator-blind, randomized, parallel group (SUNSHINE) study | Other |
| B. M. Guery, F. Anttila, V. J. Adomakoh, N. Aguado, J. M. Bisnauthsing, K. Georgopali, A. Goldenberg, S. D. Karas, A. Kazeem, G. Longshaw, C. Palacios-Fabrega, J. A. Cornely, O. A. Vehreschild, M. J. G. T. | 2017 | Extended-pulsed fidaxomicin versus vancomycin for Clostridium difficile infection in patients 60 years and older (EXTEND): a randomised, controlled, open-label, phase 3b/4 trial | Other |
| C. B. Cicerone, G. Alessandra, D. Oliva, A. Antonella, G. Valentina, T. Iebba, V. Pantanella, F. Zingaropoli, M. A. Lamonaca, L. Vullo, V. Trancassini, M. Schippa, S. Corazziari, E. S. | 2016 | Fecal microbiota transplantation via enema for recurrent clostridium difficile infection modulates the inflammatory host response and restore intestinal dysbiosis | Other |
| O. A. C. Cornely, D. Esposito, R. Poirier, A. Somero, M. Weiss, K. Sears, P. Gorbach, S. | 2012 | Clostridium difficile infection in seven European countries and North America: fidaxomicin vs. vancomycin therapy | Other |
| B. V. Pipek, Hana Fojtik, Petr Urban, Ondrej | 2022 | Faecal microbiota transplantation in the treatment of recurrent intestinal Clostridioides difficile infection - a ten-year single-center experience | Other |
| V. H. Musil, L. Vrba, M. Braunova, A. Mala, M. Holeckova, P. Krbkova, L. | 2019 | Clinical and microbiological characteristics of Clostridium difficile infection in children hospitalized at the Departement of Paediatric Infectious Diseases in Brno between 2013 and 2017 | Other |
| E. H. S. Young, K. A. Lee, G. C. Carlson, T. J. Koeller, J. M. Reveles, K. R. | 2022 | Clostridioides difficile Infection Treatment and Outcome Disparities in a National Sample of United States Hospitals | Outcomes |
| F. R. U. P. Jannat, A. | 2022 | DELAYED FECAL MICROBIOTA TRANSPLANTATION IS ASSOCIATED WITH REDUCED RATE OF CLOSTRIDIUM DIFFICILE INFECTION RECURRENCE | Outcomes |
| J. I. M. R. Bretones Pedrinaci, A. Herrera Exposito, M. Urda Romacho, J. Castro Vida, M. A. | 2022 | ANALYSIS OF MEDICAL TREATMENT, RISK FACTORS AND RECURRENCE OF CLOSTRIDIOIDES DIFFICILE NOSOCOMIAL DIARRHOEA | Outcomes |
| E. M. Armstrong, D. Pham, S. Gratie, D. Amin, A. | 2022 | Assessing the cost-effectiveness of various treatments for clostridioides difficile infections: a systematic literature review | Outcomes |
| R. M. S. Black, R. Gratie, D. | 2021 | The Burden of Illness Associated with Recurrent Clostridioides difficile Infection: A Claims-based Analysis | Outcomes |
| L. E. McDaniel, N. White, M. Obi, E. N. Chen, Y. Kohinke, R. Lockhart, E. R. | 2021 | Real-World Utilization of C. difficile Drug Treatments and Associated Clinical Outcomes in a US Hospital System | Outcomes |
| L. H. B. Hammeken, S. M. D. Hvas, C. L. Ehlers, L. H. | 2021 | Health economic evaluations comparing faecal microbiota transplantation with antibiotics for treatment of recurrent Clostridioides difficile infection: a systematic review | Outcomes |
| J. D. Worley, M. L. Cummins, C. K. DuBois, A. Klompas, M. Bry, L. | 2021 | Genomic Determination of Relative Risks for Clostridioides difficile Infection From Asymptomatic Carriage in Intensive Care Unit Patients | Outcomes |
| P. A. Geceviciene, D. Mickus, R. Dambrauskiene, A. | 2021 | Changing the treatment for Clostridium difficile infection | Outcomes |
| A. P. C. Sun, I. | 2021 | Management and outcomes of nucleic acid amplification test positive/toxin negative clostridioides difficile patients in a single center | Outcomes |
| W. R. R. Karaoui, L. B. O. Bou Daher, H. Rimmani, H. H. Rasheed, S. S. Matar, G. M. Mahfouz, R. Araj, G. F. Zahreddine, N. Kanj, S. S. Berger, F. K. Gartner, B. El Sabbagh, R. Sharara, A. I. | 2020 | Incidence, outcome, and risk factors for recurrence of nosocomial Clostridioides difficile infection in adults: A prospective cohort study | Outcomes |
| K. Sridharan | 2020 | Antimicrobials for treating Clostridium difficile infections | Outcomes |
| J. R. K. Allegretti, D. Phelps, E. Roach, B. Smith, J. Ganapini, V. C. Kassam, Z. Xu, H. Fischer, M. | 2019 | Risk of Clostridium difficile Infection with Systemic Antimicrobial Therapy Following Successful Fecal Microbiota Transplant: Should We Recommend Anti-Clostridium difficile Antibiotic Prophylaxis? | Outcomes |
| H. C. Abu-Sbeih, K. Tran, C. N. Wang, X. Lum, P. Shuttlesworth, G. Stroehlein, J. R. Okhuysen, P. C. Wang, Y. | 2019 | Recurrent Clostridium difficile infection is associated with treatment failure and prolonged illness in cancer patients | Outcomes |
| J. D. S. Tieu, S. A. Miller, J. L. Kupiec, K. E. Skrepnek, G. H. Liu, C. Smith, W. J. | 2019 | Clostridium difficile treatment in neutropenic patients: Clinical outcomes of metronidazole, vancomycin, combinations, and switch therapy | Outcomes |
| M. J. G. T. T. Vehreschild, S. Goldenberg, S. D. Thalhammer, F. Bouza, E. van Oene, J. Wetherill, G. Georgopali, A. | 2018 | Fidaxomicin for the treatment of Clostridium difficile infection (CDI) in at-risk patients with inflammatory bowel disease, fulminant CDI, renal impairment or hepatic impairment: a retrospective study of routine clinical use (ANEMONE) | Outcomes |
| L. M. Prohaska, Z. Shune, L. Singh, A. Lin, T. Abhyankar, S. Ganguly, S. Grauer, D. McGuirk, J. Clough, L. | 2018 | Retrospective evaluation of fidaxomicin versus oral vancomycin for treatment of Clostridium difficile infections in allogeneic stem cell transplant | Outcomes |
| B. K. M. Sandhu, S. M. McBride | 2018 | Clostridioides difficile | Outcomes |
| M. Y. D. Khan, A. Khurshid, T. Siddiqui, W. J. | 2018 | Comparing fecal microbiota transplantation to standard-of-care treatment for recurrent Clostridium difficile infection: A systematic review and meta-analysis | Outcomes |
| E. O. Reigadas Ramirez, M. Valerio, M. Vazquez-Cuesta, S. Alcala, L. Marin, M. Munoz, P. Bouza Santiago, E. | 2018 | Fecal microbiota transplantation for recurrent clostridium difficile infection: Experience, protocol, and results | Outcomes |
| O. P. Shogbesan, D. R. Victor, S. Jehangir, A. Fadahunsi, O. Shogbesan, G. Donato, A. | 2018 | A Systematic Review of the Efficacy and Safety of Fecal Microbiota Transplant for Clostridium difficile Infection in Immunocompromised Patients | Outcomes |
| S. J. S. Jin, K. H. Wi, Y. M. | 2018 | The effect of concomitant use of systemic antibiotics in patients with Clostridium difficile infection receiving metronidazole therapy | Outcomes |
| C. T. L. Nguyen, J. Anders, S. Garcia-Diaz, J. Staffeld-Coit, C. Hand, J. | 2018 | Comparison of outcomes with vancomycin or metronidazole for mild-to-moderate Clostridium difficile associated diarrhea among solid organ transplant recipients: A retrospective cohort study | Outcomes |
| T. Z. Chen, Q. Zhang, D. Jiang, F. Wu, J. Zhou, J. Y. Zheng, X. Chen, Y. G. | 2018 | Effect of faecal microbiota transplantation for treatment of Clostridium difficile infection in patients with inflammatory bowel disease: A systematic review and meta-analysis of cohort studies | Outcomes |
| T. R.-P. Larrainzar-Coghen, D. Fernandez-Hidalgo, N. Puig-Asensio, M. Pigrau, C. Ferrer, C. Rodriguez, V. Bartolome, R. Campany, D. Almirante, B. | 2018 | Secular trends in the epidemiology of Clostridium difficile infection (CDI) at a tertiary care hospital in Barcelona, 2006-2015: A prospective observational study | Outcomes |
| G. M. Ianiro, M. Burisch, J. Simonelli, C. Hold, G. Ventimiglia, M. Gasbarrini, A. Cammarota, G. | 2018 | Efficacy of different faecal microbiota transplantation protocols for Clostridium difficile infection: A systematic review and meta-analysis | Outcomes |
| N. O. D. Huebner, K. Henck, V. Wegner, C. Kramer, A. | 2016 | Epidemiology of multidrug resistant bacterial organisms and Clostridium difficile in German hospitals in 2014: Results from a nationwide one-day point prevalence of 329 German hospitals | Outcomes |
| G. Y. D. Chang, L. M. Banach, D. B. | 2016 | Epidemiology of Clostridium difficile infection in hospitalized oncology patients | Outcomes |
| S. S. T. Y. Yeung, J. K. Lau, T. T. Y. Forrester, L. A. Steiner, T. S. Bowie, W. R. Bryce, E. A. | 2015 | Evaluation of a Clostridium difficile infection management policy with clinical pharmacy and medical microbiology involvement at a major Canadian teaching hospital | Outcomes |
| S. K. C. Hourigan, L. A. Grigoryan, Z. Laroche, G. Weidner, M. Sears, C. L. Oliva-Hemker, M. | 2015 | Microbiome changes associated with sustained eradication of Clostridium difficile after single faecal microbiota transplantation in children with and without inflammatory bowel disease | Outcomes |
| B. J. A. Zarowitz, C. O'Shea, T. Strauss, M. E. | 2015 | Risk factors, clinical characteristics, and treatment differences between residents with and without nursing home-and non-nursing home-acquired Clostridium difficile infection | Outcomes |
| M. W. W. Causey, A. Cummings, M. Johnson, E. K. Maykel, J. A. Steele, S. | 2014 | Colonic decompression and direct intraluminal medical therapy for Clostridium difficile-associated megacolon using a tube placed endoscopically in the proximal colon | Outcomes |
| M. S. Kazanowski, S. Kinnarney, F. Grzebieniak, Z. | 2014 | Clostridium difficile: Epidemiology, diagnostic and therapeutic possibilities - A systematic review | Outcomes |
| K. K. Matsumoto, N. Shigemi, A. Ikawa, K. Morikawa, N. Koriyama, T. Orita, M. Kawamura, H. Tokuda, K. Nishi, J. Takeda, Y. | 2014 | Factors affecting treatment and recurrence of Clostridium difficile infections | Outcomes |
| C. M. Beauduy, C. | 2013 | Update on management of clostridium difficile infection | Outcomes |
| D. S. D. Clutter, Y. Merl, M. Y. Teperman, L. Press, R. Safdar, A. | 2013 | Fidaxomicin versus conventional antimicrobial therapy in 59 recipients of solid organ and hematopoietic stem cell transplantation with clostridium difficile-associated diarrhea | Outcomes |
| O. A. M. Cornely, M. A. Fantin, B. Mullane, K. Kean, Y. Gorbach, S. | 2013 | Resolution of Clostridium difficile-associated diarrhea in patients with cancer treated with fidaxomicin or vancomycin | Outcomes |
| L. T. D. Erikstrup, T. K. L. Hall, V. Olsen, K. E. P. Kristensen, B. Kahlmeter, G. Fuursted, K. Justesen, U. S. | 2012 | Antimicrobial susceptibility testing of Clostridium difficile using EUCAST epidemiological cut-off values and disk diffusion correlates | Outcomes |
| T. J. C. Louie, K. Byrne, B. Emery, J. Ward, L. Eyben, M. Krulicki, W. | 2012 | Fidaxomicin preserves the intestinal microbiome during and after treatment of clostridium difficile infection (CDI) and reduces both toxin reexpression and recurrence of CDI | Outcomes |
| H. C. Bownik, K. Aberra, F. | 2015 | Treatment for clostridium difficile infection in inflammatory bowel disease patients diagnosed in the outpatient setting: metronidazole vs. vancomycin | Outcomes |
| E. W. Aguilar-Zamora, Bart C. Torres, Roberto C. Gomez-Delgado, Alejandro Ortiz-Olvera, Nayeli Aparicio-Ozores, Gerardo Barbero-Becerra, Varenka J. Torres, Javier Camorlinga-Ponce, Margarita | 2021 | Molecular Epidemiology and Antimicrobial Resistance of Clostridioides difficile in Hospitalized Patients From Mexico | Outcomes |
| M. S. F. Wang, Samad Mangio, Joanna Pham, Kevin Lloyd, Daniel Hatch-Vallier, Brianna Johnson, Ewanah | 2020 | The Face of Clostriodes Difficile Infections in the Outpatient Setting | Outcomes |
| E. A. C. Blumberg, Gary Young, Jo-Anne H. Nguyen, M. Hong Michonneau, David Temesgen, Zelelem Origuen, Julia Barcan, Laura Obeid, Karam M. Belloso, Waldo H. Gras, Julien Corbelli, Giulio Maria Neaton, James D. Lundgren, Jens Snydman, David R. Molina, Jean-Michel Insight Clostridioides difficile Study Group | 2022 | Clostridioides difficile infection in solid organ and hematopoietic stem cell transplant recipients: A prospective multinational study | Outcomes |
| F. P. Alvarez-Lerma, M. Villasboa, A. Amador, J. Almirall, J. Posada, M. P. Catalan, M. Pascual, C. Envin-Uci Study Group | 2014 | Epidemiological study of Clostridium difficile infection in critical patients admitted to the Intensive Care Unit | Outcomes |
| J.-H. L. Chia, Hsin-Chih Su, Lin-Hui Kuo, An-Jing Wu, Tsu-Lan | 2013 | Molecular epidemiology of Clostridium difficile at a medical center in Taiwan: persistence of genetically clustering of A-B+ isolates and increase of A+B+ isolates | Outcomes |
| M. J. K. Nuijten, Josbert J. Visser, Caroline E. Redekop, Ken Claassen, Eric Speelman, Peter Pronk, Marja H. | 2015 | Cost-effectiveness in Clostridium difficile treatment decision-making | Outcomes |
| C. H. Wegner, Nils-Olaf Gleich, Sabine Thalmaier, Ulrike Kruger, Colin M. Kramer, Axel | 2013 | One-day point prevalence of emerging bacterial pathogens in a nationwide sample of 62 German hospitals in 2012 and comparison with the results of the one-day point prevalence of 2010 | Outcomes |
|  | 2018 | Fecal Microbiota Transplantation for C. Difficile Infection in Solid Organ Transplant Recipients | Study design |
| V. M. Aggarwala, I. Li, Z. Yang, C. Britton, G. J. Chen-Liaw, A. Mitcham, J. Bongers, G. Gevers, D. Clemente, J. C. Colombel, J. F. Grinspan, A. Faith, J. | 2021 | Precise quantification of bacterial strains after fecal microbiota transplantation delineates long-term engraftment and explains outcomes | Study design |
| M. B. V. Oliver, B. P. | 2022 | Fidaxomicin Use in the Pediatric Population with Clostridioides difficile | Study design |
| S. G. Johnson, D. N. Li, X. Reda, D. J. Donskey, C. J. Gupta, K. Goetz, M. B. Climo, M. W. Gordin, F. M. Ringer, R. Johnson, N. Johnson, M. Calais, L. A. Goldberg, A. M. Ge, L. Haegerich, T. | 2022 | Defining optimal treatment for recurrent Clostridioides difficile infection (OpTION study): A randomized, double-blind comparison of three antibiotic regimens for patients with a first or second recurrence | Study design |
| F. S. Cold, C. K. Christensen, A. H. Gunther, S. Petersen, A. M. Hansen, L. H. Helms, M. | 2022 | Successful treatment of Clostridioides difficile infection with single-donor faecal microbiota transplantation capsules | Study design |
| A. A. Chauhan, R. van Langenberg, D. Garg, M. | 2021 | Faecal microbiota transplantation for recurrent Clostridioides difficile infection: an Australian experience - effective, safe, yet room for improvement | Study design |
| A. A. A. Hitawala, A. Habash, A. Garg, R. Alomari, M. Sanaka, M. R. | 2020 | Clinical characteristics and outcomes of patients with clostridium difficile of the small bowel: A systematic review and meta-analysis | Study design |
| J. O. O. Alexis, E. Islam, M. Wong, C. Sobieraj, M. Kim, E. J. Hirsch, B. | 2020 | Characteristics of successful fecal microbiota transplantation | Study design |
| A. T. B. W. Abadi, A. Abdulkhakov, S. R. Rizvanov, A. A. | 2018 | Fecal Transplantation Against Recurrent Clostridium difficile Infection | Study design |
| N. D. W. Gundacker, J. B. Rodriguez, J. M. Morrow, C. D. | 2017 | Fecal Microbiota Transplant in Severe/Complicated Clostridium difficile Infection: A Retrospective Case Series | Study design |
| M. A. L. Wu, F. | 2017 | Vancomycin vs Metronidazole for Clostridium difficile infection: focus on recurrence and mortality | Study design |
| M. H. N. Granitto, C. K. | 2016 | Fecal microbiota transplantation in recurrent C. difficile infection | Study design |
| Adis Medical Writers | 2016 | Treat Clostridium difficile infection in the elderly based on disease severity and history of recurrence | Study design |
| A. P. Ponte, R. Mota, M. Silva, J. Vieira, N. Oliveira, R. Pinto-Pais, T. Fernandes, C. Ribeiro, I. Rodrigues, J. Lopes, P. Teixeira, T. Carvalho, J. | 2015 | Initial experience with fecal microbiota transplantation in Clostridium difficile infection - transplant protocol and preliminary results | Study design |
| M. D. V. Stuntz, F. | 2015 | Treating Clostridium difficile infections: Should fecal microbiota transplantation be reclassified from investigational drug to human tissue? | Study design |
| M. P. N. Kronman, H. J. Adler, A. L. Giefer, M. J. Wahbeh, G. Singh, N. Zerr, D. M. Suskind, D. L. | 2015 | Fecal microbiota transplantation via nasogastric tube for recurrent clostridium difficile infection in pediatric patients | Study design |
| A. M. Pierog, A. Reilly, N. R. | 2014 | Fecal microbiota transplantation in children with recurrent clostridium difficile infection | Study design |
| A. T. Esmaily-Fard, F. P. Crowther, D. M. Ghantoji, S. S. Adachi, J. A. Chemaly, R. F. | 2014 | The use of fidaxomicin for treatment of relapsed clostridium difficile infections in patients with cancer | Study design |
| P. M. W. Keller, M. H. | 2014 | Rational therapy of Clostridium difficile infections | Study design |
| L. F. A. Chen, D. J. | 2012 | Efficacy and safety of fidaxomicin compared with oral vancomycin for the treatment of adults with Clostridium difficile-associated diarrhea: Data from the OPT-80-003 and OPT-80-004 studies | Study design |
| Y. E. Golan, L. | 2012 | Safety and efficacy of fidaxomicin in the treatment of Clostridium difficile-associated diarrhea | Study design |
| J. W. Lancaster | 2012 | Economic impact of fidaxomicin on CDI treatment in United States | Study design |
|  | 2013 | Infusion of Donor Feces for Recurrent Clostridium difficile Infection | Study design |
|  | 2012 | Fecal Microbiota Transplant (FMT) for Relapsing C. Difficile Infection in Adults and Children Using a Frozen Inoculum | Study design |
| J. H. H. Shin, Rachel Ann Warren, Cirle Alcantara | 2021 | Hospitalized Older Patients with Clostridioides difficile Infection Refractory to Conventional Antibiotic Therapy Benefit from Fecal Microbiota Transplant | Study design |
| H. I. Peters, Arslan Miller, Emily Khalid, Sana Rahman, Omar | 2022 | Outcomes of Continuous Enteral Vancomycin Infusion in Intensive Care Unit Patients: A Novel Treatment Modality for Severe Clostridium Difficile Colitis | Study design |
| N. K. Geagan, Wei Li Adeline | 2021 | Is There a Future for a Tapered-Pulsed Fidaxomicin Regimen for Pediatric Patients With Recurrent Clostridiodes difficile Infections? | Study design |
| L. C. F. Horton, Joseph D. | 2021 | In recurrent C difficile infection, oral FMT capsules have a pooled cure rate of 82% (low-quality evidence) | Study design |
| A. M. T. Skinner, Xing Sirbu, Benjamin D. Danziger, Larry H. Gerding, Dale N. Johnson, Stuart | 2021 | A Tapered-pulsed Fidaxomicin Regimen Following Treatment in Patients With Multiple Clostridioides difficile Infection Recurrences | Study design |
| E. B. Reigadas, E. Olmedo, M. Vazquez-Cuesta, S. Villar-Gomara, L. Alcala, L. Marin, M. Rodriguez-Fernandez, S. Valerio, M. Munoz, P. | 2020 | Faecal microbiota transplantation for recurrent Clostridioides difficile infection: experience with lyophilized oral capsules | Study design |
| J. A. Oksi, Veli-Jukka Mattila, Eero | 2020 | Treatment of Clostridioides (Clostridium) difficile infection | Study design |
| J. D. Alukal, Sudhir K. Surapaneni, Balarama Krishna Le, Michelle Tabbaa, Obada Phillips, Laila Mattar, Mark C. | 2019 | Safety and efficacy of fecal microbiota transplant in 9 critically ill patients with severe and complicated Clostridium difficile infection with impending colectomy | Study design |
| S. S. P. Hota, Susan M. | 2018 | Fecal microbiota transplantation for recurrent Clostridium difficile infection | Study design |
| S. Mayor | 2016 | Donor faecal transplantation is highly curative in recurrent C difficile infection, trial finds | Study design |
| K. Senior | 2013 | Faecal transplantation for recurrent C difficile diarrhoea | Study design |
| M. T. O. Hecker, Mark E. Cadnum, Jennifer L. Jencson, Annette L. Jain, Alok K. Ho, Edith Donskey, Curtis J. | 2016 | Fecal Microbiota Transplantation by Freeze-Dried Oral Capsules for Recurrent Clostridium difficile Infection | Study design |
| B. H. Guo, C. Louie, T. Veldhuyzen van Zanten, S. Dieleman, L. A. | 2012 | Systematic review: faecal transplantation for the treatment of Clostridium difficile-associated disease | Study design |
| C. R. d. L. Kelly, Lauren Jasutkar, Niren | 2012 | Fecal microbiota transplantation for relapsing Clostridium difficile infection in 26 patients: methodology and results | Study design |
| T. B. Singh, P. Bumrah, K. Gandhi, D. Arora, T. Verma, N. Schleicher, M. Rai, M. P. Garg, R. Verma, B. Sanaka, M. R. | 2022 | Fecal Microbiota Transplantation and Medical Therapy for Clostridium difficile Infection: Meta-analysis of Randomized Controlled Trials | Study design |
| K. Z. Sehgal, I. Tariq, R. Pardi, D. S. Khanna, S. | 2022 | Systematic Review and Meta-Analysis: Efficacy of Vancomycin Taper and Pulse Regimens in Clostridioides difficile Infection | Study design |
| M. M. P. Murphy, E. Gales, M. A. | 2018 | Extended duration vancomycin in recurrent Clostridium difficile infection: a systematic review | Study design |
| F. H. Cheng, Z. Li, Z. Wei, W. | 2022 | Efficacy and safety of fecal microbiota transplant for recurrent Clostridium difficile infection in inflammatory bowel disease: a systematic review and meta-analysis | Study design |
| M. S. M. Bader, D. Hawboldt, J. Farrell, A. Alajmi, A. Maleki-Yazdi, K. | 2022 | High-Dose Vancomycin in the Treatment of Clostridioides difficile Infection: A Systematic Review and Meta-analysis | Study design |
| R. S. Tariq, T. Yadav, D. Prokop, L. J. Singh, S. Loftus, E. V. Pardi, D. S. Khanna, S. | 2021 | Outcomes of Fecal Microbiota Transplantation for C. difficile Infection in Inflammatory Bowel Disease: A Systematic Review and Meta-analysis | Study design |
| J. P. Leung, S. | 2021 | A Systematic Review of Fecal Microbiota Transplantation Versus Vancomycin for Treatment of Recurrent Clostridioides difficile Infection | Study design |
| F. B. Cold, S. M. D. Dahlerup, J. F. Petersen, A. M. Hvas, C. L. Hansen, L. H. | 2021 | Systematic review with meta-analysis: encapsulated faecal microbiota transplantation - evidence for clinical efficacy | Study design |
| R. A. V. Pomares Bascunana, V. Sheth, C. C. | 2021 | Effectiveness of fecal microbiota transplant for the treatment of Clostridioides difficile diarrhea: a systematic review and meta-analysis | Study design |
| F. G. Dembrovszky, N. Szakacs, Z. Hegyi, P. Kiss, S. Farkas, N. Molnar, Z. Imrei, M. Dohos, D. Peterfi, Z. | 2021 | Fecal Microbiota Transplantation May Be the Best Option in Treating Multiple Clostridioides difficile Infection: A Network Meta-Analysis | Study design |
| R. L.-V. Tariq, M. Tahir, M. W. Orenstein, R. Pardi, D. S. Khanna, S. | 2021 | Efficacy of oral vancomycin prophylaxis for prevention of Clostridioides difficile infection: a systematic review and meta-analysis | Study design |
| S. M. D. L. Baunwall, M. M. Eriksen, M. K. Mullish, B. H. Marchesi, J. R. Dahlerup, J. F. Hvas, C. L. | 2020 | Faecal microbiota transplantation for recurrent Clostridioides difficile infection: An updated systematic review and meta-analysis | Study design |
| S. E. K. Babar, B. El Iskandarani, M. Haddad, I. Imam, Z. Alomari, M. Myers, J. Moorman, J. | 2020 | Oral vancomycin prophylaxis for the prevention of Clostridium difficile infection: A systematic review and meta-analysis | Study design |
| C. L. Du, Y. Walsh, S. Grinspan, A. M. | 2020 | Oral Fecal Microbiota Transplantation Capsules Are Effective and Safe for Recurrent Clostridioides difficile Infection: A Systematic Review and Meta-Analysis | Study design |
| F. S. Khademi, A. | 2019 | The prevalence of antibiotic-resistant Clostridium species in Iran: a meta-analysis | Study design |
| A. A. A. Alhifany, A. R. Almangour, T. A. Shahbar, A. N. Abraham, I. Alessa, M. Alnezary, F. S. Cheema, E. | 2019 | Comparing the efficacy and safety of faecal microbiota transplantation with bezlotoxumab in reducing the risk of recurrent Clostridium difficile infections: A systematic review and Bayesian network meta-analysis of randomised controlled trials | Study design |
| T. B. Beinortas, N. E. Wilcox, M. H. Subramanian, V. | 2018 | Comparative efficacy of treatments for Clostridium difficile infection: a systematic review and network meta-analysis | Study design |
| P. N. Le, V. T. Mullen, P. D. Deshpande, A. | 2018 | Cost-Effectiveness of Competing Treatment Strategies for Clostridium difficile Infection: A Systematic Review | Study design |
| Y. T. Igarashi, S. Enoki, Y. Taguchi, K. Matsumoto, K. Ohge, H. Suzuki, H. Nakamura, A. Mori, N. Morinaga, Y. Yamagishi, Y. Yoshizawa, S. Yanagihara, K. Mikamo, H. Kunishima, H. | 2018 | Oral vancomycin versus metronidazole for the treatment of Clostridioides difficile infection: Meta-analysis of randomized controlled trials | Study design |
| U. A. Iqbal, H. Karim, M. A. | 2018 | Safety and efficacy of encapsulated fecal microbiota transplantation for recurrent Clostridium difficile infection: A systematic review | Study design |
| T. L. Yuan, Z. | 2018 | Fecal microbiota transplantation as a treatment for gastrointestinal diseases: A systemic review and meta-analysis | Study design |
| H. E. M. Burton, S. A. Watt, M. | 2017 | A Systematic Literature Review of Economic Evaluations of Antibiotic Treatments for Clostridium difficile Infection | Study design |
| M. N. W. Quraishi, M. Bhala, N. Moore, D. Price, M. Sharma, N. Iqbal, T. H. | 2017 | Systematic review with meta-analysis: the efficacy of faecal microbiota transplantation for the treatment of recurrent and refractory Clostridium difficile infection | Study design |
| L. V. O. McFarland, M. Dinleyici, E. C. Goh, S. | 2016 | Comparison of pediatric and adult antibiotic-associated diarrhea and Clostridium difficile infections | Study design |
| B. C. M. Chapman, H. B. Overbey, D. M. Morton, A. P. Harnke, B. Gerich, M. E. Vogel, J. D. | 2016 | Fecal microbiota transplant in patients with Clostridium difficile infection: A systematic review | Study design |
| Y. T. C. Li, H. F. Wang, Z. H. Xu, J. Fang, J. Y. | 2016 | Systematic review with meta-analysis: Long-term outcomes of faecal microbiota transplantation for Clostridium difficile infection | Study design |
| C. C. Tang, L. Xu, Y. Xie, L. Sun, P. Liu, C. Xia, W. Liu, G. | 2016 | The incidence and drug resistance of Clostridium difficile infection in Mainland China: a systematic review and meta-analysis | Study design |
| X. B. Di, N. Zhang, X. Liu, B. Ni, W. Wang, J. Wang, K. Liang, B. Liu, Y. Wang, R. | 2015 | A meta-analysis of metronidazole and vancomycin for the treatment of Clostridium difficile infection, stratified by disease severity | Study design |
| R. L. Li, L. Lin, Y. Wang, M. Liu, X. | 2015 | Efficacy and safety of metronidazole monotherapy versus vancomycin monotherapy or combination therapy in patients with clostridium difficile infection: A systematic review and meta-analysis | Study design |
| N. R. Bagdasarian, K. Malani, P. N. | 2015 | Diagnosis and treatment of clostridium difficile in adults: A systematic review | Study design |
| J. C. J. O'Horo, K. Kunzer, B. Safdar, N. | 2014 | Treatment of recurrent Clostridium difficile infection: A systematic review | Study design |
| G. I. Cammarota, G. Gasbarrini, A. | 2014 | Fecal microbiota transplantation for the treatment of clostridium difficile infection: A systematic review | Study design |
| S. L. Sha, J. Chen, M. Xu, B. Liang, C. Wei, N. Wu, K. | 2014 | Systematic review: Faecal microbiota transplantation therapy for digestive and nondigestive disorders in adults and children | Study design |
| Z. L. Kassam, C. H. Yuan, Y. Hunt, R. H. | 2013 | Fecal microbiota transplantation for clostridium difficile infection: Systematic review and meta-analysis | Study design |
| E. H. A. Otete, A. S. Jones, H. Bolton, K. J. Jordan, C. W. Boswell, T. C. Wilcox, M. H. Ferguson, N. M. Beck, C. R. Puleston, R. L. | 2013 | Parameters for the mathematical modelling of clostridium difficile acquisition and transmission: A systematic review | Study design |
| D. W. S. W. Crook, A. Kean, Y. Weiss, K. Cornely, O. A. Miller, M. A. Esposito, R. Louie, T. J. Stoesser, N. E. Young, B. C. Angus, B. J. Gorbach, S. L. Peto, T. E. A. | 2012 | Fidaxomicin versus vancomycin for clostridium difficile infection: Meta-analysis of pivotal randomized controlled trials | Study design |
| K. Z. P. Vardakas, K. A. Patouni, K. Rafailidis, P. I. Samonis, G. Falagas, M. E. | 2012 | Treatment failure and recurrence of Clostridium difficile infection following treatment with vancomycin or metronidazole: A systematic review of the evidence | Study design |
| L. L. Yang, Wenrui Zhang, Xianzhuo Tian, Jinhui Ma, Xiaojia Han, Lulu Wei, Huaping Meng, Wenbo | 2022 | The evaluation of different types fecal bacteria products for the treatment of recurrent Clostridium difficile associated diarrhea: A systematic review and network meta-analysis | Study design |
| A. S. M. T. Ramesh, Carlos Jamil, Dawood Tran, Hadrian Hoang-Vu Mansoor, Mafaz Butt, Samia Rauf Satnarine, Travis Ratna, Pranuthi Sarker, Aditi Khan, Safeera | 2022 | Role of Fecal Microbiota Transplantation in Reducing Clostridioides difficile Infection-Associated Morbidity and Mortality: A Systematic Review | Study design |
| K. T. Gupta, Mamatha Nazir, Armaan M. Koganti, Bhavya Memon, Marrium S. Aslam Zahid, Muhammad Bin Shantha Kumar, Vignarth Mostafa, Jihan A. | 2022 | Fecal Microbiota Transplant in Recurrent Clostridium Difficile Infections: A Systematic Review | Study design |
| Y. N. Y. Song, David Yi Veldhuyzen van Zanten, Sander Wong, Karen McArthur, Eric Song, Claire Zhao Ianiro, Gianluca Cammarota, Giovanni Kelly, Colleen Fischer, Monika Russell, Lindsey Kao, Dina | 2022 | Fecal Microbiota Transplantation for Severe or Fulminant Clostridioides difficile Infection: Systematic Review and Meta-analysis | Study design |
| E. N. V. Tixier, Elijah Luo, Yuying Grinspan, Lauren Tal Du, Charles H. Ungaro, Ryan C. Walsh, Samantha Grinspan, Ari M. | 2022 | Systematic Review with Meta-Analysis: Fecal Microbiota Transplantation for Severe or Fulminant Clostridioides difficile | Study design |
| S. K. Saha, Saloni Tariq, Raseen Schuetz, Audrey N. Tosh, Pritish K. Pardi, Darrell S. Khanna, Sahil | 2019 | Increasing antibiotic resistance in Clostridioides difficile: A systematic review and meta-analysis | Study design |
| C.-Y. S. Chiu, Amara Feinstein, Addi Hennessey, Karen | 2019 | Effective Dosage of Oral Vancomycin in Treatment for Initial Episode of Clostridioides difficile Infection: A Systematic Review and Meta-Analysis | Study design |
| L. A. A. Al Momani, Omar Boonpheng, Boonphiphop Gabriel, Joseph Gabriel Young, Mark | 2018 | Fidaxomicin vs Vancomycin for the Treatment of a First Episode of Clostridium Difficile Infection: A Meta-analysis and Systematic Review | Study design |
| D. R. Drekonja, Jon Gezahegn, Selome Greer, Nancy Shaukat, Aasma MacDonald, Roderick Rutks, Indy Wilt, Timothy J. | 2015 | Fecal Microbiota Transplantation for Clostridium difficile Infection: A Systematic Review | Study design |
